# The Yo-Yo Intermittent Tests: A Systematic Review and Structured Compendium of Test Results

**DOI:** 10.3389/fphys.2018.00870

**Published:** 2018-07-05

**Authors:** Boris Schmitz, Carina Pfeifer, Kiana Kreitz, Matthias Borowski, Andreas Faldum, Stefan-Martin Brand

**Affiliations:** ^1^Institute of Sports Medicine, Molecular Genetics of Cardiovascular Disease, University Hospital Muenster, Muenster, Germany; ^2^Institute of Biostatistics and Clinical Research, University of Muenster, Muenster, Germany

**Keywords:** Yo-Yo IR, Yo-Yo IE, Yo-Yo test, performance testing, field test, physical fitness, exercise capacity

## Abstract

**Background:** Although Yo-Yo intermittent tests are frequently used in a variety of sports and research studies to determine physical fitness, no structured reference exists for comparison and rating of test results. This systematic review of the most common Yo-Yo tests aimed to provide reference values for test results by statistical aggregation of published data.

**Methods:** A systematic literature search for articles published until August 2017 was performed in MEDLINE, Web of Science, SPORTDiscus and Google Scholar. Original reports on healthy females and males ≥16 years were eligible for the analysis. Sub-maximal test versions and the Yo-Yo Intermittent Recovery Level 1 Children's test (YYIR1C) were not included.

**Results:** 248 studies with 9,440 participants were included in the structured analysis. The Yo-Yo test types most frequently used were the Yo-Yo Intermittent Recovery Level 1 (YYIR1, 57.7%), the Yo-Yo Intermittent Recovery Level 2 (YYIR2, 28.0%), the Yo-Yo Intermittent Endurance Level 2 (YYIE2, 11.4%), and the Yo-Yo Intermittent Endurance Level 1 (YYIE1, 2.9%) test. For each separate test, reference values (global means and percentiles) for sports at different levels and both genders were calculated.

**Conclusions:** Our analysis provides evidence that Yo-Yo intermittent tests reference values differ with respect to the type and level of sport performed.The presented results may be used by practitioners, trainers and athletes to rate Yo-Yo intermittent test performance levels and monitor training effects.

## Introduction

### Rationale

Since the introduction of the Yo-Yo Intermittent (YYI) test as a field test method in the 1990s, an evolution of the Yo-Yo test family has occurred (Bangsbo, 1994; Krustrup et al., 2003; Bangsbo et al., 2008). Today, Yo-Yo test variants are extensively used to assess physical fitness in different sports and populations.

In comparison to standard laboratory testing, field test methods have been developed to bring exercise testing to a more realistic setting with the additional practical benefit to determine exercise capacity also in groups (Krustrup et al., 2003). Modern wearable devices such as heart rate (HR)/ECG monitors, 3D gyroscopes and spirometers provide additional options for these testing methods. The majority of field tests are performed as exhaustive running tests measuring total distance covered, maximal test duration or maximal speed as easy-to-access outcome parameters. While continuous running tests (Balke, 1963; Léger and Boucher, 1980; Ramsbottom et al., 1987) such as the Cooper test (Cooper, 1968) are typically performed on 200 or 400 m tracks, multi-stage shuttle-run tests consist of repeated short distance runs (Léger and Lambert, 1982; Krustrup et al., 2003; Da Silva et al., 2011). In this regard, shuttle-run tests also include an agility component immanent to sports that involve intermittent exercise such as soccer or basketball (Bangsbo et al., 2008).

The Yo-Yo Intermittent Recovery (YYIR) test has been developed based on the maximal multistage 20-m shuttle-run test (20-MST) (Bangsbo et al., 2008) introduced by Léger and Lambert (Léger and Lambert, 1982) and was modified by an active recovery period by Bangsbo (Bangsbo et al., 2008). The main objective of the YYIR test was to measure the ability to repeatedly perform intense exercise including the potential to rapidly recover from such exercise (Krustrup et al., 2003). During the YYIR test, participants perform repeated 2 × 20-m runs at progressively increasing speed, intermitted by 10-s periods of active recovery (2 × 5 m) (Bangsbo et al., 2008). The test is performed until total exhaustion of the participant is reached (i.e., as maximal performance test). The pace is controlled by an automated acoustic device, indicating start, turn and finish but it is mandatory that the test is supervised by experienced personnel. YYIR performance is defined as the maximal distance covered (m) which is achieved when a participants has failed twice to reach the finishing line in time or discontinuous the test due to perceived exhaustion. The YYIR test can be performed at two different levels, designated as Yo-Yo Intermittent Recovery Level 1 (YYIR1) test and Yo-Yo Intermittent Recovery Level 2 (YYIR2) test. In detail, the YYIR1 test starts at a lower speed level (Castagna et al., 2006b) with 4 running bouts at 10–13 km·h^−1^ (0–160 m) followed by 7 runs at 13.5–14 km·h^−1^ (160–440 m), proceeding with stepwise 0.5 km·h^−1^ speed increments after every 8 running bouts until exhaustion (Krustrup et al., 2003). By contrast, the YYIR2 test starts at a higher speed level and two initial runs of 13 and 15 km·h^−1^, respectively, followed by two runs at 16 km·h^−1^, three runs at 16.5 km·h^−1^, 4 runs at 17.0 km·h^−1^, proceeding with stepwise 0.5 km·h^−1^ speed increments after every 8 running bouts until exhaustion (Krustrup et al., 2006; Bangsbo et al., 2008). Based on this difference, the YYIR1 test has been suggested as a method primarily to test endurance capacity, whereas the YYIR2 test was introduced to determine the ability to repeatedly perform intense exercise with a high anaerobic energy contribution (Bangsbo et al., 2008). Two additional common test modifications exist for the determination of endurance capacity, the Yo-Yo Intermittent Endurance Level 1 (YYIE1) test (starting at 8 km·h^−1^) (Castagna et al., 2006a) and the Yo-Yo Intermittent Endurance Level 2 (YYIE2) test (starting at 11.5 km·h^−1^) (Bradley et al., 2014). In both YYIE tests, the active recovery period is shortened to 5 s (2 × 2.5 m) and the stepwise increase is reduced from 0.5 to 0.25 km·h^−1^ (Reilly et al., 2000).

### Objective

Despite the intensive use of Yo-Yo tests in athletic training, sports sciences and sports medicine, a comprehensive summary of test results is missing from the literature. The aim of this systematic review and analysis was to establish a structured list of reference values for the most common Yo-Yo tests to be used in practical and scientific applications. The current study will therefore provide a basis to interpret physical performance of healthy individuals ranging from sedentary and recreationally active subjects to amateur and elite athletes. In combination with known values for test reproducibility, the generated data will also help to interpret the individual response to specific training interventions.

## Methods

### Study design and participants eligibility criteria

Any original article reporting on performance testing using either of the four Yo-Yo test variants including the YYIR1, YYIR2, YYIE1, and YYIE2 test was considered for the analysis. Sub-maximal test versions, the Yo-Yo Intermittent Recovery Level 1 Children's test (YYIR1C) (Ahler et al., 2012; Bendiksen et al., 2013) or other test modifications were not included in the analysis. The original multistage 20-MST (Léger and Lambert, 1982) without recovery periods, which has occasionally been described as “Yo-Yo test” was not included in this analysis. Only reports on healthy humans (*n* ≥ 5) with no disability were eligible. Data on participants ≥16 years of age were included based on the analysis of age-related Yo-Yo test performance variation by Deprez et al. (2012, 2014, 2015b). Articles had to be original research (not a review, book [chapter] or conference abstract) and be written in English. Gray literature, including theses, reference lists or websites were not included. Articles were excluded if (1) they were not available as full-text (after an attempt to contact the corresponding author), (2) did not report original performance test results (i.e., reporting percent changes, etc.), (3) presented performance data only in figures, (4) reported test results in any other format than maximal distance (m), time (min), speed (m·s^−1^) or stages, or if (5) the type of test performed was not clearly defined. Articles were also excluded if the number of tested subjects or test performance mean or standard deviation (SD)/standard error of the mean (SEM) were missing or not clearly reported in the full-text. The eligibility criteria were selected also in accordance with the quality assessment (see below). This study is part of a larger project on the use of YYI tests to determine exercise capacity in adults and children and as tool to measure effects in randomized controlled trails (RCTs). A specific review protocol will be available upon request.

### Search strategy and data sources

**Figure 1:**
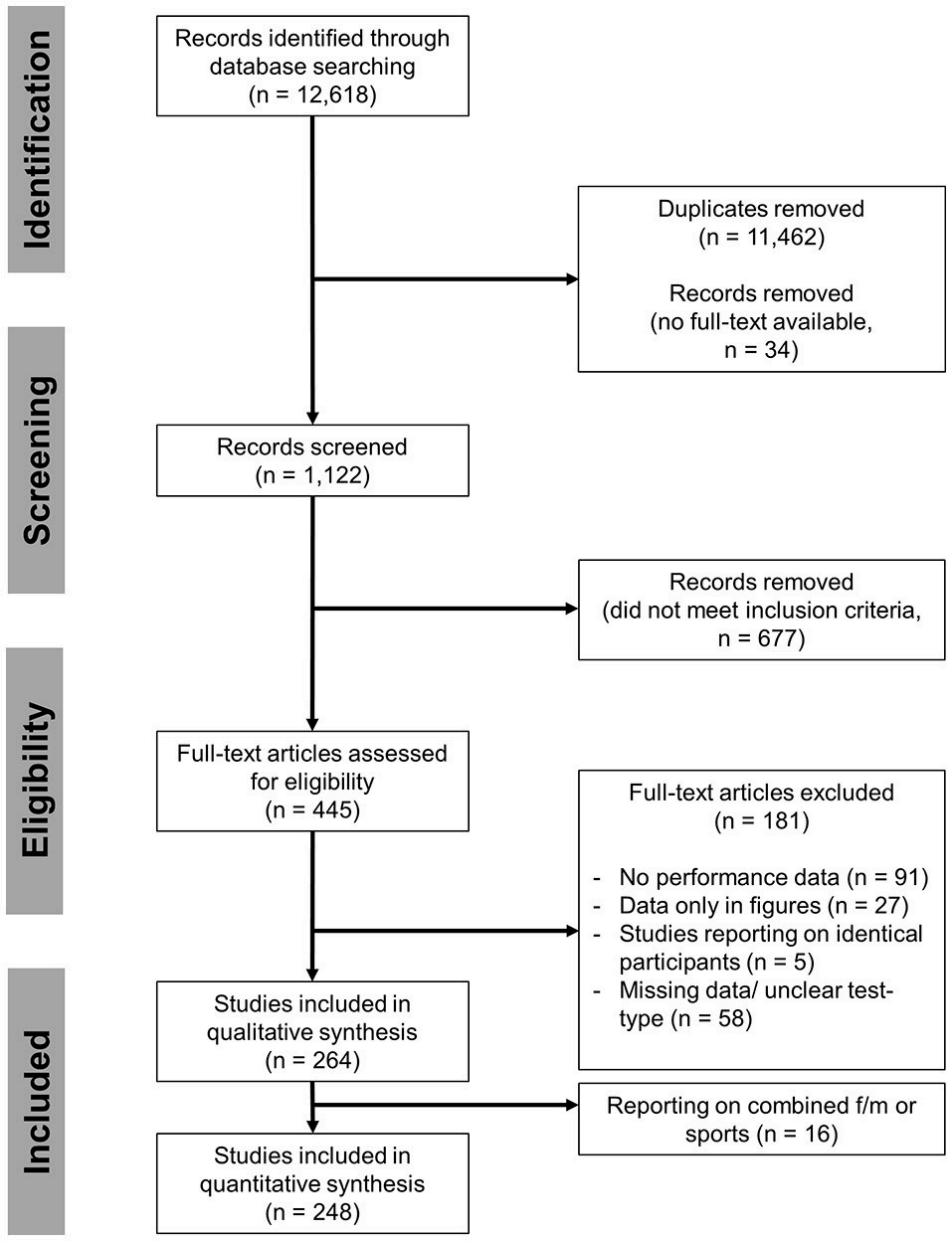
PRISMA flow chart illustrating record identification, screening and inclusion.

A systematic search of the literature was conducted (CP) using PubMed (MEDLINE database), SPORTDiscus with Full Text, Google Scholar and Web of Science for all records published until August 2017. Databases were searched using the following key words: “Yo-Yo intermittent test” or “Yo-Yo intermittent” or “Yo-Yo intermittent recovery test” or “Yo-Yo intermittent recovery” or “Yo-Yo intermittent endurance test” or “Yo-Yo intermittent endurance” or “YYIR1” or “YYIR2” or “YYIR” or “YYIE1” or “YYIE2” or “YYIE” or “Yo-Yo IR” or “Yo-Yo IE” or “Yo-Yo IR1” or “Yo-Yo IE1” or “Yo-Yo IR2” or “Yo-Yo IE2” or “Yo-Yo test” or “YoYo test.” Manual searches were also performed using references from identified articles. Two authors (BS and CP) performed full-text screening on potential relevant reports. The individual steps of report identification, screening and processing are documented according to the PRISMA flow-chart (Figure 1) (Moher et al., 2009) with modifications. Search results and fulfillment of eligibility criteria were discussed if unclear (BS and CP) until consensus was achieved and upon disagreement, a third person was consulted to determine inclusion.

### Study selection and data extraction

Data were extracted by two reviewers (BS and CP) and tables were created including information on first author, year of publication, information on description of participants (total number, anthropometric data, competition level), type of sport, test type and performance data. If articles reported on test results of different age groups, separate data on participants ≥16 years of age were extracted if possible. In case of reporting on general intra- and inter-seasonal Yo-Yo test performance changes, the best test data were extracted. Inter-seasonal changes were analyzed separately as stated below (Bangsbo et al., 2008). In case of redundant data reported in separate publications, only data from the earlier report were extracted. If both females and males or different types of sports were tested in combination and could not be extracted separately, the test results were not included in the analysis and results were reported in online Supplementary Tables 1–4.

### Definitions

Individuals were classified as top-elite (international professional level, where indicated), elite (professional level), sub-elite (national level), amateur (non-professional, regional level), recreational or inactive based on the author's description. Studies presenting data on combined levels were analyzed and reported using the respective level combination as identifier. In case of imprecise, uncommon, unclear/ conflicting or missing descriptions of test participants, full-texts were screened by two reviewers (BS and CP) for additional information including club or union associations, training patterns or other information. Classification by a nearest-neighbor approach according to the documented performance data was performed when no useful information on participants could be identified. Player position-specific data were extracted separately if available. If performance data were reported in investigations involving any type of experimental condition with hypothesized effect on test performance, only pre-intervention data were extracted if available or data of (untreated) control groups were used. If intra-seasonal changes were reported, all data were extracted and the highest reported test result was included in the main analysis.

### Quality assessment

The methodological quality of the studies was assessed using the critical appraisal tool established by Brink and Louw (2012). The tool consists of 13 items assessing the quality of a study. The individual items can be scored as “yes,” “no,” or “not applicable.” For our analysis, we determined (in accordance with the above mentioned inclusion criteria) that the following items (8 out of 13) had to be scored “yes”: a detailed description of the subject sample was available, the qualification or competence of rater(s) performing the test was clarified, the reference standard was explained, the stability of the variable beaning measured was taken into account, the execution of the test was described in sufficient detail to permit replication, the execution of the reference standard was described in sufficient detail, withdrawals from the study were explained, the statistical methods were appropriate. Studies were rated by two reviewers (BS and CP). Disagreements were resolved by discussion if necessary. The researchers were not blinded to study authors, results or publication journal.

### Statistical data analyses

Data for Yo-Yo test performance was analyzed as maximal distance (m). Maximal test duration (min), speed (m·s^−1^) or stages were recalculated for comparison. SD was calculated from SEM using the equation $SD=\;\sqrt{n}\cdot SEM\;$ where n is the number of subjects. For each category (i.e., for each combination of sport, sex, and level) a global mean and global SD were calculated based on the reported means and SDs of the individual studies (or study subgroups) within this category. This was done assuming that the individuals from each study within the same category belong to the same population and that their test results were drawn from the same normal distribution. Each global mean was calculated as weighted mean of the individual reported means, with weights built by the number of subjects per study. Each global SD was calculated using the formula described in online Supplementary File 1. Finally, the global mean and global SD were translated into normal quantiles/ percentiles (20, 40, 60, 80%) for each category. Furthermore, separate forest plots were created for each category showing the individual mean and 95% confidence interval of each study as well as the calculated global mean and global SD. The *I*^2^ statistic was calculated for each category to quantify the heterogeneity within the respective individual study results (Higgins and Thompson, 2002). All forest plots including *I*^2^ statistics are available in the online repository.

## Results

### Study selection and characteristics

**Figure 2:**
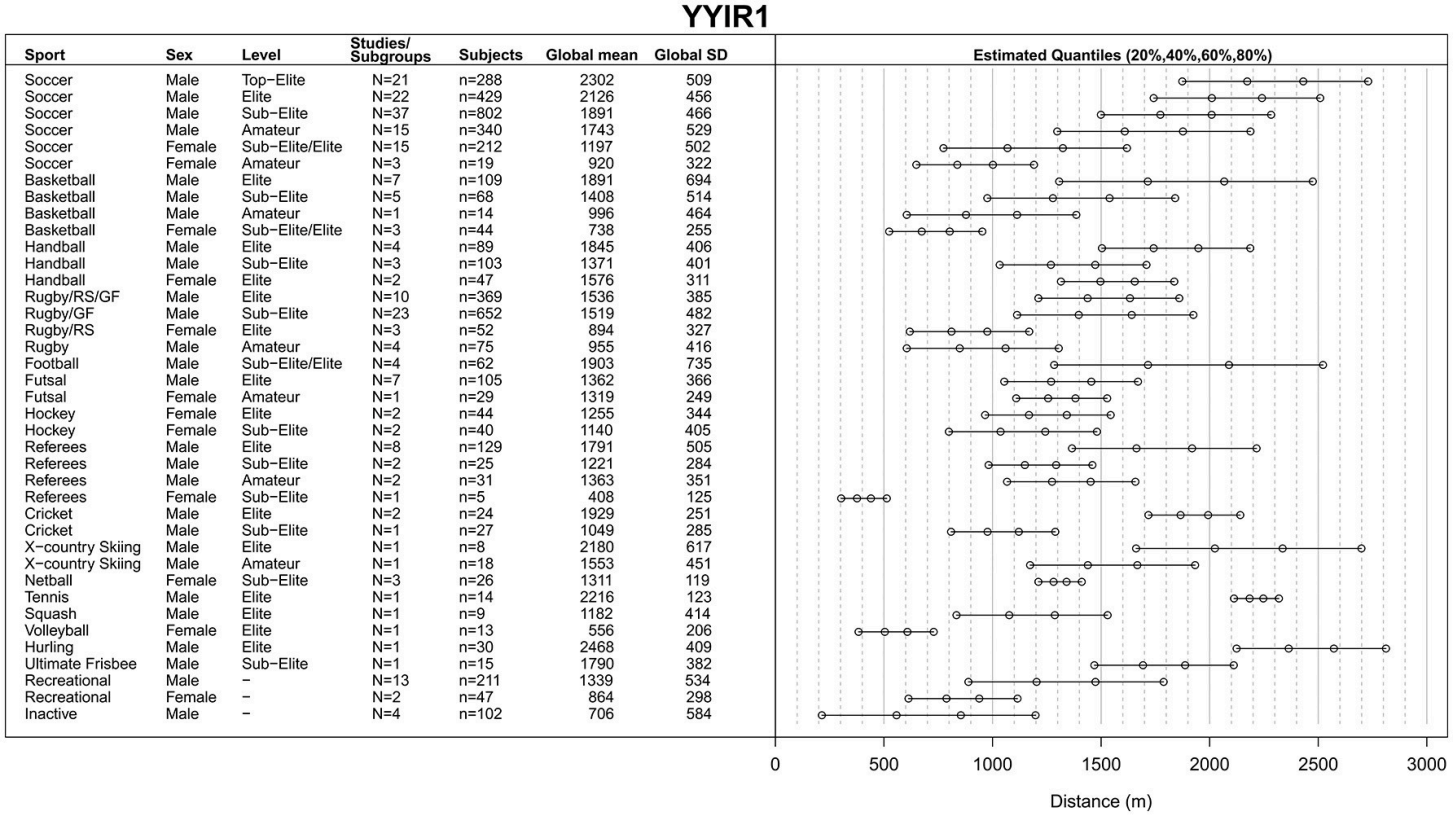
Yo-Yo Intermittent Recovery Level 1 (YYIR1) test results. RS, Rugby sevens; GF, Gaelic football.

**Figure 3:**
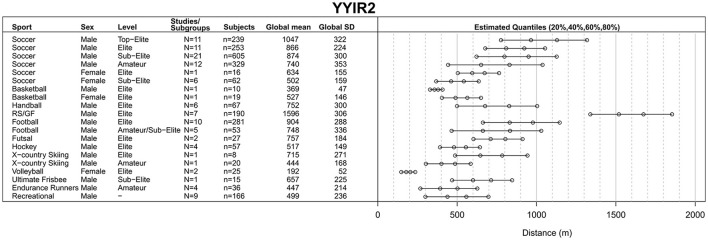
Yo-Yo Intermittent Recovery Level 2 (YYIR2) test results. RS, Rugby sevens; GF, Gaelic football.

**Figure 4:**
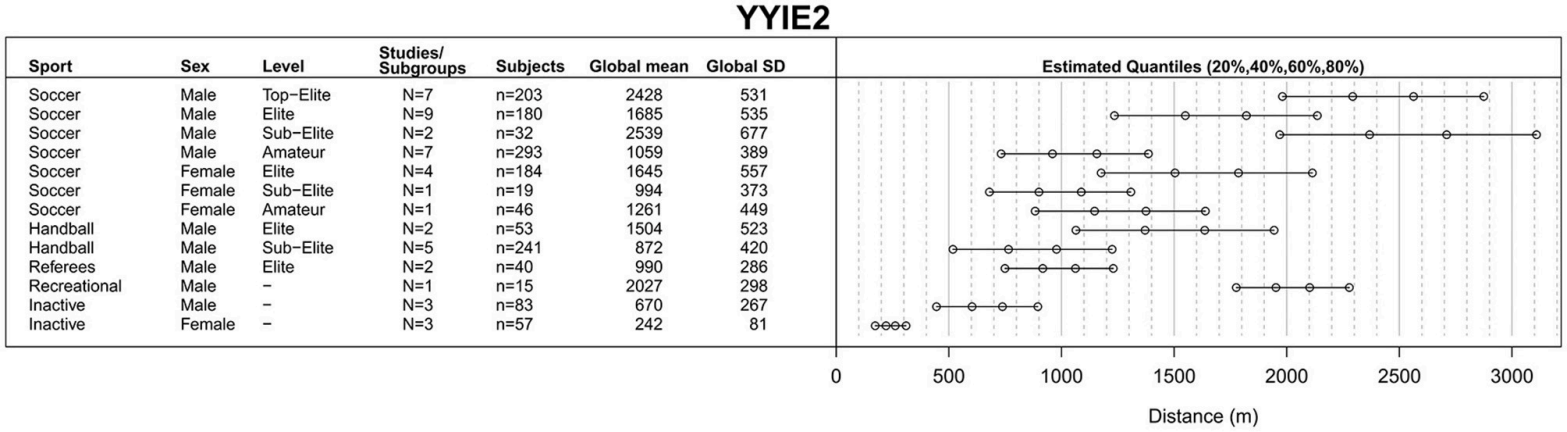
Yo-Yo Intermittent Endurance Level 2 (YYIE2) test results.

**Figure 5:**
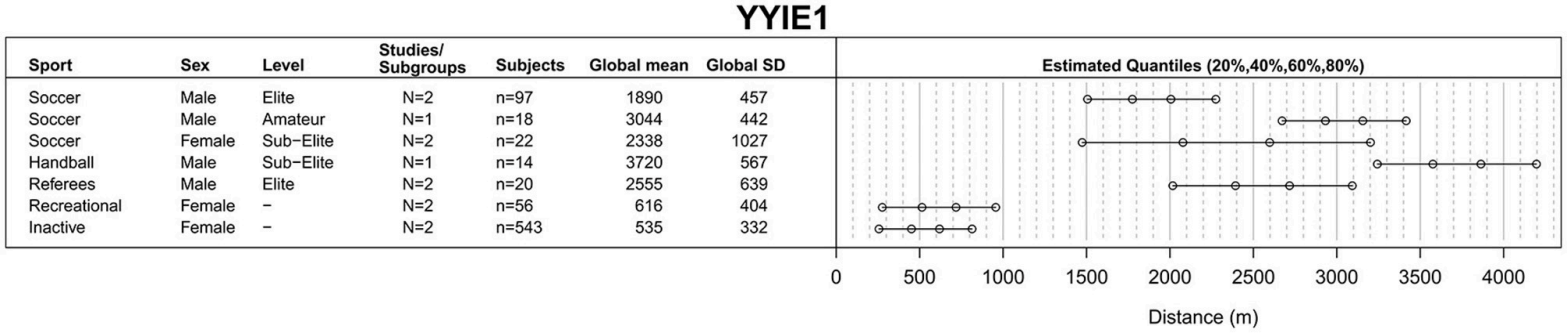
Yo-Yo Intermittent Endurance Level 1 (YYIE1) test results.

The process of study identification, selection and final inclusion is presented in Figure 1. During the screening stage, 677 records were removed for not meeting the inclusion criteria of which 86 studies reported only on children and adolescents <16 years of age (Figure 1). Of 445 full-text articles assessed for eligibility, 264 studies were included in the qualitative synthesis. Each individual included study (or sub-study) with information on author, subgroup or performance level, sex, mean age and test results is presented in online Supplementary Tables 1–4, sorted by the test performed (including 16 studies reporting on combined sex or sports). Of these, 248 studies were included in the quantitative synthesis to obtain a global mean and SD for each category, i.e., for each combination of type of sport, performance level and sex. Global means for the YYIR1 test are presented in Figure 2, for the YYIR2 test in Figure 3, for the YYIE2 test in Figure 4 and for the YYIE1 test in Figure 5.

### Results by Yo-Yo test-type

#### YYIR1 test

For the computation of YYIR1 test global means, SDs and quantiles (Figure 2), 239 studies or subgroups with a total of 4,726 participants (median of reported age means = 21.1, inter quartile range [IQR] of reported age means = 17.8–24.5 years) were used (Krustrup and Bangsbo, 2001; Krustrup et al., 2003; Mohr et al., 2003, 2010, 2016; Weston et al., 2004; Castagna et al., 2005, 2006b, 2008; Atkins, 2006; Thomas et al., 2006; Rampinini et al., 2007, 2008, 2010; Mujika et al., 2009; Ben Abdelkrim et al., 2010; Chaouachi et al., 2010; Dupont et al., 2010; Veale et al., 2010; Wong et al., 2010; Buchheit et al., 2011; Chtourou et al., 2011; Chuman et al., 2011; Cobley et al., 2011; Markovic and Mikulic, 2011; Matthys et al., 2011; Roberts et al., 2011; Spencer et al., 2011; Ueda et al., 2011; Alemdaroglu et al., 2012; Boullosa et al., 2012, 2013a,b; Cihan et al., 2012; Cone et al., 2012; Deprez et al., 2012, 2014, 2015a,b; Heaney, 2012; Ingebrigtsen et al., 2012, 2014; Lim, 2012; Scanlan et al., 2012, 2014; Shalfawi et al., 2012, 2013; Teplan et al., 2012a,b, 2013; Vernillo et al., 2012; Berdejo-del-Fresno and González-Ravé, 2013; Cullen et al., 2013; Faude et al., 2013; Higham et al., 2013; Idrizovic and Raickovic, 2013; Manzi et al., 2013; Oliveira et al., 2013; Román-Quintana et al., 2013; Wylie et al., 2013; Yuki et al., 2013; Casamichana et al., 2014, 2015; Clarke et al., 2014; Fabregat-Andres et al., 2014; Fanchini et al., 2014, 2015a,b; Hammouda et al., 2014; Hermassi et al., 2014, 2015, 2016; Idrizovic, 2014; Karavelioglu, 2014; Martínez-Lagunas and Hartmann, 2014; Michalsik et al., 2014; Mohr and Krustrup, 2014; Raman et al., 2014; Rollo et al., 2014; Santone et al., 2014; Soares-Caldeira et al., 2014; Till et al., 2014, 2015, 2016, 2017; Afyon et al., 2015; Attene et al., 2015; Campos-Vazquez et al., 2015a,b; Campos Vázquez et al., 2017; Darrall-Jones et al., 2015, 2016; Hasegawa and Kuzuhara, 2015; Iacono et al., 2015; Johnston et al., 2015; Köklü et al., 2015; Krustrup and Mohr, 2015; Lopez-Segovia et al., 2015; Macpherson and Weston, 2015; Mohr, 2015; Moss et al., 2015; Nakamura et al., 2015, 2016; Noon et al., 2015; Shultz et al., 2015; Abad et al., 2016; Bizati, 2016; Bruce and Moule, 2016; Castillo et al., 2016, 2017; Coratella et al., 2016; Eaton et al., 2016; Flatt and Esco, 2016; Furlan et al., 2016; Jones et al., 2016; Karsten et al., 2016; Kilding et al., 2016; Lockie et al., 2016d; Matzenbacher et al., 2016; McIntosh et al., 2016; Nyberg et al., 2016; Pareja-Blanco et al., 2016, 2017; Purkhús et al., 2016; Rabbani and Buchheit, 2016; Roe and Malone, 2016; Sánchez-Sánchez et al., 2016; Sant'anna and de Souza Castro, 2016; Schwesig et al., 2016; Sharpe and Macias, 2016; Smith et al., 2016; Taylor et al., 2016; Vescovi, 2016; Aloui et al., 2017; Aoki et al., 2017; Bonato et al., 2017; Dinardi et al., 2017; Dixon et al., 2017; Ferioli et al., 2017; Hamlin et al., 2017; Henrique Borges et al., 2017; Kavaliauskas et al., 2017; Malone et al., 2017; Nyakayiru et al., 2017; Risso et al., 2017; Rowat et al., 2017; Schmitz et al., 2017; Sparks et al., 2017; Veness et al., 2017).

#### YYIR2 test

Computation of YYIR2 test global means, SDs and quantiles (Figure 3) involved 116 studies/subgroups reporting on 2,478 participants (median age = 23.2 years, IQR = 20.7–26.0 years) (Young et al., 2005; De Souza et al., 2006; Krustrup et al., 2006; Thomas et al., 2006; Mohr et al., 2007, 2016; Iaia et al., 2008, 2015, 2017; Morton et al., 2009; Rampinini et al., 2010; Rebelo et al., 2010; Thomassen et al., 2010; Christensen et al., 2011; Chuman et al., 2011; Mooney et al., 2011, 2013a,b; Roberts et al., 2011; Ueda et al., 2011; Gunnarsson et al., 2012; Ingebrigtsen et al., 2012, 2013, 2014; Nakamura et al., 2012; Saunders et al., 2012; Wells et al., 2012, 2014; Bassini et al., 2013; Buchheit et al., 2013; McGawley and Andersson, 2013; Mohr and Krustrup, 2013, 2014, 2016; Owen et al., 2013; Pivovarniček et al., 2013; Yuki et al., 2013; Fanchini et al., 2014; Karavelioglu et al., 2014; Lollo et al., 2014; Miloski et al., 2014; Nunes et al., 2014; Sampaio et al., 2014; Skovgaard et al., 2014; Brocherie et al., 2015a,b; Cholewa et al., 2015; Coelho et al., 2015; Gatterer et al., 2015; Hogarth et al., 2015a,b; Jamurtas et al., 2015; Krustrup and Mohr, 2015; Leme et al., 2015; Mara et al., 2015; Matta et al., 2015; McLean et al., 2015; Michalsik et al., 2015; Mohr, 2015; Rogan, 2015; Stein et al., 2015; Boer and Van Aswegen, 2016; Bouaziz et al., 2016; Chan et al., 2016; Inness et al., 2016; Joo, 2016; Kilit and Arslan, 2016; Lockie et al., 2016a,b,c,d; Nogueira et al., 2016; Purkhús et al., 2016; Stevens et al., 2016; Veugelers et al., 2016; Kelly and Collins, 2017).

#### YYIE2 test

Computation of YYIE2 test global means, SDs and quantiles (Figure 4) included 48 studies/subgroups and 1,466 participants (median age = 23.0 years, IQR = 18.3–26.0 years) (Aziz et al., 2005; Rampinini et al., 2007; Bangsbo et al., 2010; Brito et al., 2010; Krustrup et al., 2010a,b,c, 2015; Randers et al., 2010, 2013; Ascensão et al., 2011; Bradley et al., 2011, 2013, 2014; Rebelo et al., 2011, 2013; Silva et al., 2011, 2013; Gibson et al., 2013; Dixon, 2014; Massuça et al., 2014; Massuca et al., 2015; Matta et al., 2014; Póvoas et al., 2014; Kvorning et al., 2017).

#### YYIE1 test

Computation of YYIE1 test global means, SDs and quantiles (Figure 5) involved 12 studies/subgroups reporting on 770 participants (median age = 21.3 years, IQR = 18.8–44.3 years) (Metaxas et al., 2005; Castagna et al., 2006a; Lategan et al., 2011; Rowan et al., 2012; Deliceoglu, 2013; Mohr et al., 2014; Akashi et al., 2015; Fløtum et al., 2016; Julian et al., 2017; Krustrup et al., 2017; Seidelin et al., 2017).

### Results by player position

Player position data was available for 727 subjects (males and females) who performed the YYIR1 test including soccer, Gaelic football, rugby and netball (online Supplementary Figure 1). For the YYIR2 test, position data of 70 subjects (all male) was available for soccer, handball and Gaelic football (online Supplementary Figure 2). For the YYIE2, player position data of 341 individuals (all male) was analyzed for soccer and handball (online Supplementary Figure 3). No player position data was available for the YYIE1 test.

### Intra-seasonal test results

To analyse the effects of intra-seasonal differences in YYIR test results, data of male soccer players who had performed either the YYIR1 (307 individual data points available, online Supplementary Figure 4) or YYIR2 (847 individual data points available, online Supplementary Figure 5) at a minimum of two different occasions during a single season were extracted. For other test types, sports or females, data were insufficient for an intra-seasonal analysis.

### Risk of bias

The quality of the included studies was assessed using the critical appraisal tool by Brink and Louw (2012), which does not incorporate a quality score and the impact of each item needs to be considered individually. Since the presented analysis was a study of one-point test result examination, the following 5 items were found not applicable: testing of interrater reliability and blinding of raters to the test results, blinding of raters to their own prior findings, variation of the order of examination, evaluation of time period between reference standard and index test, and independence of reference standard and index test. All other items were scored with “yes” by definition of the inclusion criteria. Other criteria for the assessment of bias, such us blinding of test participants or raters to the test results were unfeasible for this type of test.

## Discussion

### Summary of main findings

This systematic review synthesized YYI test results of 248 studies comprising performance data of 9,440 participants. A statistical aggregation of published data resulted in reference lists showing global means, SDs and quantiles for the four most common YYI test variants structured by sport, performance level and sex. Data on sedentary and recreationally active females and males were also included in the analysis.

### Limitations

Some limitations for the presented analysis may exist. First, a high level of heterogeneity was noted in some subgroups, most likely based on the different demands that exist between different national leagues, etc. Inter-study differences including different participant characterization and description as well as definitions and nomenclature may have affected the classification process. Second, and besides methodological quality assessment of the selected studies, selection bias within individual studies may have occurred. For the presentation of Yo-Yo test results selection bias could occur in terms of test termination (i.e., the test is not stopped at the earliest time point violating the test requirements) or data partitioning and reporting of data subsets (i.e., reporting on best test results). Reporting and publication bias may have affected the present analysis since some data/ studies may have remained unreported or were not published because of unexpected/ contradictory, negative or not significant test results. Furthermore, the record search was limited to studies published in English and inclusion of data reported in other languages may have altered the results of subgroups with smaller sample sizes. The necessary pooling and transformation of data may also have affected the presented results to some extent.

### General analysis

The most frequently used YYI test to determine physical fitness in individuals ≥16 years of age was the YYIR1 test (57.7%), followed by the YYIR2 test (28.0%), the YYIE2 test (11.4%), and the YYIE1 test (2.9%). As the YYI test was originally proposed to test aerobic performance of soccer players, the largest dataset of test results was available for female and male soccer players (and referees) followed by different other types of sports marked by high intermittent exercise such as basketball, handball, rugby etc. Our study also documented that YYI variants have been used in other sports including futsal, cross-country skiing, endurance running, recreational team sports and a number of other activities on different levels. With respect to the documented test results and overall tests analyzed in this study, it can be generally stated that men performed better than women, elite athletes performed better than sub-elite or amateur athletes and higher test performance was seen for intermittent sports, which is in line with an earlier analysis by Bangsbo et al. (2008). However, individual performance from athletes of other sports with a high aerobic component such as cross-country skiing was also documented at high levels. Overall, the data also provide evidence that even within the category of sports marked by high intermittent exercise, differences in YYI test results can be detected, which might reflect the multifactorial nature in sports and the need for specific reference values. We have also noted that in individual cases, athletes or teams at lower competitive levels may reach high or very high test performance values, documenting outstanding physical performance. For example, our analysis on YYIE2 test results revealed that sub-elite soccer players may reach test results comparable to top-elite players. In addition, our analysis documented that physical fitness of recreationally active but also inactive subjects can be tested by either of the four Yo-Yo variants.

### Position and seasonal data

Since different demands exist for the individual positions in team sports, we also generated position-specific global means, SDs and quantiles for each test where available. We only detected slight differences between outfield players in general, most likely reflecting the need for high endurance capacity of the entire team for dynamic and successful competition which is achieved by team-specific rather than player position-specific training. During the data extraction and evaluation we noticed that most studies explicitly excluded goalkeepers from their analysis. Our analysis on available data revealed that goalkeepers tended to lower performance over the analyzed test types and sports (soccer, gaelic football, and handball) and their performance should thus indeed be evaluated separately from the outfield players. This example illustrates that individual training modalities such as specific goalkeeper training which besides aerobic and sprint training includes a considerable amount of reflex training, ball stopping, etc. are reflected by YYI test performance.

Intra-seasonal changes were limited to an analysis of male soccer players in the YYIR1 and YYIR2 test as sufficient data for females, other sports or test types was not available. The analysis suggested that players' YYIR test results tend to be generally lower when assessed during preseason and increase with regular seasonal training as would be expected (Bangsbo et al., 2008). However, this data should be interpreted with care as scheduled seasonal testing routines enable participants (players and coaches) to control their test performance toward increasing results to indicate adequate training response.

### Test validity

The YYIR test was initially introduced to measure the ability to repeatedly perform intense exercise including the potential to rapidly recover from such exercise with a particular focus on intermittent sports such as soccer or basketball (Krustrup et al., 2003; Bangsbo et al., 2008). Thus, the validity can be tested by comparison of YYIR test performance and performance during actual competitive games. For the YYIR1 test, the reported significant correlation with high-intensity running (>15 km·h^−1^) during a soccer match was *r* = 0.71 (*n* = 18, elite soccer players) (Krustrup et al., 2003) and the correlation with high-intensity activity and total distance covered during a soccer match was *r* = 0.77 and *r* = 0.65, respectively (*n* = 19, young soccer players, age ~14 years) (Castagna et al., 2009). For the YYIR2 test, the correlation with the time above 85% of HR_max_ during a soccer match was *r* = 0.71 (*n* = 18, young soccer players, age ~14 years), the correlation with high-speed running (>14.4 km·h^−1^) during small-sided game was *r* = 0.70 (*n* = 113, soccer players, age range ~17–24 years) (Stevens et al., 2016), the correlation with load·min^−1^ during football matches was *r* = 0.77 (*n* = 20, elite football players, age ~22 years) (Mooney et al., 2013b) and the correlation with high-speed running (>15 km·h^−1^) during football matches was *r* ≥ 0.62 (*n* = 15, elite football players, age ~22 years) (Mooney et al., 2013a). For the YYIE2 test, the reported correlation with high-intensity running (≥19.8 km·h^−1^) during professional soccer matches was *r* = 0.54 (*n* = 22, Premier League soccer players, age ~26 years) and *r* = 0.64 (*n* = 21, Championship soccer players, age ~25 years) (Bradley et al., 2013). The observed correlations between the different YYI tests and match performance parameters can thus be rated as moderate to strong.

In addition, Yo-Yo test variants have been suggested to determine aerobic performance capacity and thus to estimate maximal oxygen uptake (VO_2max_). For the YYIR1 test, the reported significant correlation with VO_2max_ determined in a laboratory setting was *r* = 0.71 (*n* = 15, elite soccer players) (Krustrup et al., 2003). This finding was confirmed by a report on the significant correlation of YYIR1 test performance and laboratory VO_2max_ in recreationally active subjects (*r* = 0.87, *n* = 19) (Thomas et al., 2006). Castagna et al. (2006b), however, did not detect a significant correlation between YYIR1 test performance and laboratory VO_2max_ (*r* = 0.46, *n* = 24, amateur soccer players, age ~25 years). For the YYIE2 test, the same group reported a significant correlation with laboratory VO_2max_ at *r* = 0.75 (*n* = 24, amateur soccer players, age ~25 years) (Castagna et al., 2006b). For the YYIR2 test, Thomas et al. (2006) reported no significant correlation between test performance and laboratory VO_2max_ (*r* = 0.43, *n* = 19, recreationally active men). For the YYIE1 test, correlation with laboratory VO_2max_ was *r* = 0.63 (*n* = 62, young soccer players, age ~14 years) (Wong et al., 2011) and *r* = 0.65 (*n* = 18, youth soccer players, age ~16 years) (Castagna et al., 2006a). The latter study, however, also measured VO_2max_ directly during the YYIE1 and reported that VO_2max_ was not significantly associated with the distance covered during the test (*r* = 0.53). Interestingly, they also noted that maximal respiratory variables determined in the laboratory setting and YYIE1 test were not significantly different (Castagna et al., 2006a). The authors concluded that YYIE1 performance does not exclusively depend on maximal aerobic power. With respect to these results, future studies on the YYI tests are needed and should involve wearable spirometry devices to gain better insight into the cardio-respiratory responses during the different tests.

### Test reproducibility

It has been suggested that changes in athletes' performance in response to different training strategies may be monitored by the YYI test (Krustrup et al., 2006). To this respect, the level of test reproducibility is important and has been analyzed in a number of different populations during the past two decades. For the YYIR1, the reported coefficients of variation (CV) were 4.9% (*n* = 13) (Krustrup et al., 2003), 7.3% (*n* = 24, young soccer players) (Fanchini et al., 2014), 8.1% (*n* = 28) (Bangsbo et al., 2008) and 8.7% (*n* = 16, recreationally active men) (Thomas et al., 2006). For the YYIR2, the CVs ranged from 7.1% (*n* = 24, young soccer players) (Fanchini et al., 2014) to 9.6% (*n* = 29, normally trained male subjects and elite soccer players) (Krustrup et al., 2006), 10.4% (*n* = 53) (Bangsbo et al., 2008) and 12.7% (*n* = 17, recreationally active men) (Thomas et al., 2006). For the YYIE1, CV of 5.7% was observed in young soccer players (age ~14, *n* = 51) (Wong et al., 2011) and for the YYIE2, CV of 4.5% was observed in domestic female soccer players (*n* = 27) (Bradley et al., 2014). With respect to age (and potentially training experience in intermittent sports) as a major confounding factor for test reproducibility, Póvoas et al. reported on Yo-Yo tests in schoolboys (9–16 years) and observed that the CV decreased with increasing age from 11.1 to 8.5% (Póvoas et al., 2016). Deprez and colleagues reported that the CV for the YYIR1 decreased with increasing age from 17.3 to 7.9% in 78 sub- and non-elite soccer players (age-range 11.3–17.2 years) (Deprez et al., 2014). It is therefore important to note that smaller effects on physical fitness might not be detectable using either of the YYI test variants in any population. With respect to practical implications this would translate into certain minimal detectable changes as follows. Exemplified, for male sub-elite soccer players tested by the YYIR1 test (global mean distance = 1,891 m) and application of the lowest CV (4.9%) the estimated minimal change indicating a suggestive meaningful improvement would be >92 m and thus ≥5 full 20-m shuttles. The application of an estimated mean over the available reported CVs of ~7.3% for the same group would results in an estimated minimal change indicating a likely meaningful improvement of >138 m and thus ≥7 full 20-m YYIR1 test shuttles. For the YYIR2 test (lowest reported CV = 7.1%) and male sub-elite soccer players, the estimated minimal change indicating a suggestive meaningful improvement would be >134 m and thus ≥ 7 full 20-m shuttles. The application of an estimated mean over the available reported CVs of ~9.9% for the same group would results in an estimated minimal change indicating a likely meaningful improvement of >187 m and thus ≥10 full 20-m YYIR1 test shuttles. This might also have consequences for YYI test-based analysis of interventional programs in the field of primary prevention of diseases and underlines the need for controlled trials with adequate group sizes.

### Potential test limitations and practical instructions

Per definition, the YYIR1/2 and YYIE1/2 test are designed as maximal performance tests (Krustrup et al., 2003; Bangsbo et al., 2008). It is thus an essential requirement that the tested participant is willing to perform until total exhaustion. If the participant resists to this concept, for example to achieve underestimation of the training status at the beginning of a training season or to prevent from exhaustive exercise during match preparations, the test result will be of limited value. It is thus important that participants are highly motivated and are familiar with the general concept of the test and its application. Participants also need to understand the test settings and rules including the criteria of termination (i.e., failure to reach the finishing line within time, etc.), which can best be realized by familiarization with the test. To achieve maximal performance, motivation of the participant including verbal encouragement during the test as well as (competitive) group settings might be helpful. With respect to the latter aspect, it is not only mandatory that the test is supervised by experienced personnel but it should be a general standard that the test is supervised and documented by at least two raters. This will allow for the prevention of errors in general such as documentation errors or test procedure errors (i.e., failure of the participant to cover the full running/ recovery distance) and will also limit the effect of observer errors including failure of timely test termination (which will lead to overestimation of the result). Moreover, we suggest that raters which document test results are blinded to the findings of other raters and interrater reliability is reported (Brink and Louw, 2012) together with other important test parameters (including location, number of supervisors/ assistants, group size, etc.). Finally, modern devices such as wearable HR monitors, 3D gyroscopes and (in track) timing gates might further increase the value of Yo-Yo test variants.

## Conclusions

The most frequently used Yo-Yo intermittent test to determine physical fitness in individuals ≥16 years of age was the YYIR1 test (57.7%), followed by the YYIR2 test (28.0%), the YYIE2 test (11.4%) and the YYIE1 test (2.9%). Our analysis provides evidence that YYI tests reference values differ depending on gender as well as type and level of sport performed. In general, higher test performance was seen for intermittent sports but YYI test variants may also be used to determine physical fitness in other sports as well as recreationally active and inactive subjects. The presented results may be used by practitioners, trainers and athletes to rate Yo-Yo intermittent test performance and monitor training effects. With regard to varying reproducibility values, caution is warranted when using YYI tests to determine performance changes in response to different training strategies.

## Author contributions

BS, S-MB, AF, MB, and CP contributed to the conception and design of the study. CP performed the systematic search. CP and BS screened records and edited the data. BS, MB, CP, and KK performed the data analysis. BS and CP wrote the manuscript. All authors contributed to the drafting and revision of the manuscript. All authors approved the final version of the manuscript.

## Conflict of interest statement

The authors declare that the research was conducted in the absence of any commercial or financial relationships that could be construed as a potential conflict of interest.
