# Supplementary material for: The Yo-Yo Intermittent Tests: A Systematic Review and Structured Compendium of Test Results
Source: Front Physiol. 2018 Jul 5;9:870. doi: 10.3389/fphys.2018.00870 (PMC6041409; doi:10.3389/fphys.2018.00870)
Supplement: Supplementary file 1 [file Table_1.PDF]

Supplemental table 1: Yo-Yo Intermittent Recovery Level 1 test (YYIR1)

Study	Subgroup/ level	Sex	Age, y (±SD)	Result, m (±SD)
Noon, et al., 2015	Soccer, academy players with category-two status, UK	m	17.0 ± 1.0	3150.0 ± 269.0
Mohr, et al., 2010	Soccer, highly-trained professionals, second and third division, Spain	m	19.3 ± 1.34	2950.0 ± 424.85
Campos-Vazquez, et al., 2015	Soccer, professionals, La Liga, second division, Spain	m	26.7 ± 4.5	2880.0 ± 374.0
Nyberg, et al., 2016	Soccer, highly-trained semiprofessionals, first division, second league, Denmark	m	23.5 ± 4.0	2803.0 ± 330.0
Campos-Vázquez, et al., 2017	Soccer, professionals, Liga Adelante, second division, Spain	m	26.2 ± 3.7	2782.2 ± 411.8
Spencer, et al., 2011	Soccer, highly-trained	m	U18	2715.0 ± 547.0
Ben Abdelkrim, et al., 2010	Basketball, national team, Tunisia	m	25.4 ± 3.0	2619.0 ± 731.0
Boullosa, et al., 2013	Soccer, elite, first division, Spain	m	24.0 ± 4.0	2600.0 ± 786.0
Lategan, et al., 2011	Referees, soccer, national level, South Africa	m	-	2595.39 ± 33.13
Deprez, et al., 2015	Soccer, high level professionals, Belgium	m	18.1 ± 0.4	2546.67 ± 31.67
Buchheit, et al., 2011	Soccer, well-trained, first division, Faroe Island, second division, Denmark	m	26.2 ± 5.0	2527.0 ± 321.0
Bizati, et al., 2016	Soccer, professional, Super League, first division, wingers, Turkey	m	22.5 ± 3.7	2515.0 ± 191.49
Martin, et al., 2017	Team sport athletes (australian football, basketball, hockey, netball), state level, Australia	m	16.87 ± 1.2	2507.0 ± 413.0
Lategan, et al., 2011	Referees, soccer, national level, South Africa	m	-	2480.0 ± 454.9
Bizati, et al., 2016	Soccer, professional, Super League, first division, full backs, Turkey	m	21.75 ± 2.97	2475.0 ± 138.92
Malone, et al., 2017	Hurling, first division, Ireland	m	26.5 ± 3.2	2468.0 ± 409.0
Bizati, et al., 2016	Soccer, professional, Super League, first division, midfielders, Turkey	m	23.71 ± 4.31	2465.71 ± 62.77
Casamichana, et al., 2015	Soccer, amateur, regional level	m	23.4 ± 4.5	2438.5 ± 540.3
Mujika, et al., 2009	Soccer, Primera División, first division, Spain	m	23.8 ± 3.4	2414.0 ± 456.0
Deprez, et al., 2015	Soccer, professionals, U17 soccer club, Belgium	m	16.2 ± 0.6	2404.0 ± 346.33
Román-Quintana, et al., 2013	Soccer, amateur, regional level	m	24.4 ± 4.7	2394.0 ± 621.1
Fanchini, et al., 2015	Soccer, semiprofessionals, high-level, fifth division, Italy	m	24.0 ± 6.0	2385.0 ± 412.0
Ueda, et al., 2011	Soccer, elite college-level, Japan	m	20.8 ± 0.4	2369.0 ± 319.2
Manzi, et al., 2013	Soccer, elite professionals, Seria A, first	m	28.4 ± 3.2	2366.0 ± 409.0

	division, Italy			
Roe, et al., 2016	Gaelic football, sub-elite, first division, Ireland	m	24.5 ± 6.5	2365.4 ± 489.6
Chuman, et al., 2011	Soccer, professionals, first division, Japan	m	U17	2365.0 ± 251.0
Casamichana, et al., 2014	Soccer, semiprofessionals	m	22.7 ± 4.3	2360.0 ± 638.4
Deprez, et al., 2015	Soccer, high level, defender	m	17.6 ± 0.6	2353.0 ± 391.0
Spencer, et al., 2011	Soccer, highly-trained	m	U17	2340.0 ± 401.0
Deprez, et al., 2015	Soccer, high level, midfielder	m	17.7 ± 0.6	2332.0 ± 458.0
Weston, et al., 2004	Referees, soccer, FIFA international, Belgium	m	37.8 ± 4.12	2330.0 ± 268.0
Deprez, et al., 2015	Soccer, high level, attacker	m	17.6 ± 0.6	2316.0 ± 540.0
Ueda, et al., 2011	Soccer, non-elite college-level, Japan	m	20.1 ± 0.6	2315.2 ± 346.3
Bizati, et al., 2016	Soccer, professional, Super League, first division, forwards, Turkey	m	24.14 ± 3.72	2305.71 ± 56.23
Campos-Vazques, et al., 2015	Soccer, top U19 category, Spain	m	18.0 ± 0.9	2297.0 ± 302.0
Chaouachi, et al., 2010	Soccer, elite, national level, Tunisia	m	19.0 ± 1.0	2289.0 ± 406.0
Cone, et al., 2012	Soccer, highly-trained, National Collegiate Athletic Association Division 1, USA	m	-	2283.3 ± 393.9
Mohr, et al., 2003	Soccer, top-class professionals, European elite teams, Italian league, European Championsleague	m	26.4 ± 3.82	2260.0 ± 339.41
Higham, et al., 2013	Rugby sevens, national squad, international level, Australia	m	21.9 ± 2.0	2256.0 ± 268.0
Mohr, et al., 2016	Soccer, elite semiprofessionals	m	26.2 ± 5.67	2236.0 ± 248.46
Rampinini, et al., 2010	Soccer, professionals	m	25.0 ± 4.0	2231.0 ± 294.0
Kilit, et al., 2016	Tennis, professionals, International Tennis Numbers ranging from 1 to 2	m	23.0 ± 1.9	2215.7 ± 123.3
Ferioli, et al., 2017	Basketball, semiprofessionals, Serie B, second division, Italy	m	23.3 ± 4.7	2205.0 ± 397.0
Bizati, et al., 2016	Soccer, professional, Super League, first division, center backs, Turkey	m	24.5 ± 3.11	2195.0 ± 161.97
Chaabene, et al., 2015	Karatekas, national and international level, Tunisia	both	24.2 ± 5.7	2186.0 ± 643.0
Coelho, et al., 2015	Soccer, professionals, second division, Brazil	m	21.2 ± 3.7	2182.6 ± 184.3
Yuki, et al., 2013	Cross-country skiing, elite, high-school level, Japan	m	16.9 ± 0.3	2180.0 ± 617.0
Deprez, et al., 2012	Soccer, elite, first division, Belgium	m	17.4 ± 0.53	2179.18 ± 50.53

Ferioli, et al., 2017	Basketball, professionals, Serie A and Serie A2, first and second division, Italy	m	25.6 ± 6.0	2154.0 ± 362.0
Deprez, et al., 2014	Soccer, elite, U17 first Division	m	16.6 ± 0.6	2151.0 ± 373.0
Rampinini, et al., 2008	Soccer, professionals	m	17.6 ± 0.5	2150.0 ± 327.0
Campos-Vazques, et al., 2015	Soccer, top U19 category, Spain	m	18.2 ± 0.7	2145.0 ± 461.5
Castagna, et al., 2006	Soccer, amateur	m	25.6 ± 5.1	2138.0 ± 364.0
Rampinini, et al., 2007	Soccer, amateur	m	24.5 ± 4.1	2132.0 ± 380.0
Markovic, et al., 2011	Soccer, first division, Croatia	m	U19	2128.0 ± 326.0
Faude, et al., 2013	Soccer, third division, Switzerland	m	22.6 ± 2.4	2120.0 ± 533.0
Rollo, et al., 2014	Soccer, sub-elite, first division, UK	m	-	2105.0 ± 222.0
Schmitz, et al., 2017	Adults, moderatly-trained	m	22.38 ± 2.31	2095.24 ± 40.74
Mujika, et al., 2009	Soccer, La Tercera Division, fourth division, Spain	m	18.4 ± 0.9	2092.0 ± 260.0
Fanchini, et al., 2015	Soccer, second division, Switzerland	m	16.0 ± 1.0	2083.0 ± 349.0
Lockie, et al., 2016	Soccer, college-level, first division, USA	f	21.6 ± 0.89	2056.0 ± 394.56
Bonato, et al., 2017	Soccer, college-level, Italy	m	23.0 ± 3.0	2045.0 ± 312.0
Mohr, et al., 2003	Soccer, professional league, Denmark	m	26.5 ± 4.9	2040.0 ± 293.94
Hermassi, et al., 2016	Handball, top level, first division, Germany	m	25.2 ± 5.1	2038.0 ± 537.0
Dupont, et al., 2010	Soccer, amateur	m	23.2 ± 3.5	2034.0 ± 367.0
Ingebrigsten, et al., 2012	Soccer, elite, first division, Norway	m	26.0 ± 7.0	2033.0 ± 416.0
Haugen, et al., 2014	Soccer, highest junior division, Norway	both	17.0 ± 1.0	2030.0 ± 599.0
Mohr, et al., 2014	Soccer, semiprofessionals, top league, external midfielders, Faroe Islands	m	25.8 ± 4.1	2012.0 ± 447.0
Fabregat-Andres, et al., 2014	Soccer, professionals, second division, Spain	m	20.1 ± 1.8	2006.3 ± 282.9
Teplan, et al., 2012	Soccer, elite, U17 category, Czech republic	m	16.4 ± 0.3	2002.5 ± 331.9
Ben Abdelkrim, et al., 2010	Basketball, elite, U20 national team, Tunisia	m	19.5 ± 4.0	2000 ± 642.0
Cihan, et al., 2012	Soccer, prof, first division, midfielder Turkey	m	25.8 ± 1.64	1987.0 ± 260.3
Bonato, et al., 2017	Soccer, college-level, Italy	m	21.0 ± 3.0	1987.0 ± 302.0

Weston, et al., 2004	Referees, soccer, elite, first and second division, Belgium	m	39.28 ± 4.21	1985.0 ± 279.0
Bishop, et al., 2017	Cricket, elite, UK	f	26.2 ± 5.3	1956.0 ± 147.31
Teplan, et al., 2012	Soccer, U17 first division, Czech Republic	m	16.4 ± 0.3	1952.94 ± 80.85
Rollo, et al., 2014	Soccer, sub-elite, first division, UK	m	-	1948.0 ± 250.0
Hammouda, et al., 2014	Soccer, professioanls, first division, Tunesia	m	17.52 ± 0.2	1947.64 ± 459.0
Teplan, et al., 2012	Soccer, U17 national level, Czech Republic	m	16.5 ± 0.3	1940.0 ± 360.43
Castillo, et al., 2017	Referees, soccer, elite, national level, field referees, Spain	m	29.6 ± 7.8	1936 ± 469,61
Attene, et al., 2015	Basketball, U17 national championship, Italy	m	16.0 ± 1.0	1933.0 ± 560.0
Cihan, et al., 2012	Soccer, prof, first division, defender players Turkey	m	27.1 ± 2.69	1925.0 ± 168.9
Rabbani, et al., 2016	Soccer, U19 first division, Iran	m	17.8 ± 4.0	1920.0 ± 264.0
Mohr, et al., 2014	Soccer, semiprofessionals, top league, central midfielders, Faroe Islands	m	25.8 ± 4.1	1913.0 ± 148.0
Veale, et al., 2010	Australian football, elite, Australia	m	16.6 ± 0.5	1910.0 ± 230.0
Chtourou, et al., 2011	Soccer, football squads, Tunesia	m	17.6 ± 0.6	1903.64 ± 16.64
Veness, et al., 2017	Cricket, elite, professional club, UK	m	21.0 ± 8.0	1892.0 ± 357.0
Castagna, et al., 2005	Referees, soccer, top level, Series A and B, Italy	m	37.5 ± 4.5	1874.0 ± 431.0
Krustrup, et al., 2003	Adults	m	25.0-36.0	1873.5 ± 290.25
Mohr, et al., 2014	Soccer, semiprofessionals, top league, attackers, Faroe Islands	m	25.8 ± 4.1	1865.0 ± 336.0
Mohr, et al., 2014	Soccer, semiprofessionals, top league, fullbacks, Faroe Islands	m	25.8 ± 4.1	1841.0 ± 385.0
Matthys, et al., 2011	Handball, elite, Belgium	m	16.8 ± 0.5	1840.0 ± 270.0
Taylor, et al., 2016	Soccer, semiprofessionals	m	24.0 ± 4.0	1830.0 ± 274.0
Rampinini, et al., 2010	Soccer, amateur	m	25.0 ± 5.0	1827.0 ± 292.0
Macpherson, et al., 2015	Soccer, semiprofessionals, ninth tier, UK	m	-	1817.0 ± 513.0
Bizati, et al., 2016	Soccer, professional, Super League, first division, goalkeeper, Turkey	m	22.67 ± 2.52	1813.33 ± 20.55
Markovic, et al., 2011	Soccer, first division, Croatia	m	U18	1800.0 ± 415.0
McIntosh, et al., 2016	Rugby, elite rugby union players, New	m	20.3 ± 1.2	1800.0 ± 212.0

	Zealand			
Krustrup, et al., 2001	Referees, soccer, top-class, Superliga and first division, Denmark	m	-	1792.0 ± 406.67
Kustrup, et al., 2015	Ultimate frisbee, club-level, UK	m	20.5 ± 0.8	1790.0 ± 382.0
Hermassi, et al., 2015	Handball, national level youth, Tunisia	m	17.2 ± 0.7	1772.0 ± 343.0
Lopez-Segovia, et al., 2015	Soccer, U20 national level, Spain	m	18.4 ± 0.8	1760.0 ± 329.0
Teplan, et al., 2013	Soccer, elite, highest U18 category, Czech Republic	m	17.6 ± 0.3	1760.0 ± 314.64
Macpherson, et al., 2015	Soccer, semiprofessionals, ninth tier, UK	m	-	1754.0 ± 672.0
Hermassi, et al., 2014	Handball, elite, national level	m	17.7 ± 0.3	1745.45 ± 04.08
McIntosh, et al., 2016	Rugby, elite rugby union players, New Zealand	m	21.9 ± 2.8	1745.0 ± 529.0
Cihan, et al., 2012	Soccer, prof, first division, forwards Turkey	m	28.0 ± 1.87	1744.0 ± 360.6
Castillo, et al., 2016	Referees, soccer, national level, Spain	m	30.0 ± 7.1	1742.86 ± 95.93
Ingebrigsten, et al., 2014	Soccer, third-highest division, Norway	m	22.0 ± 5.0	1736.0 ± 443.0
Koklu, et al., 2015	Soccer, academy league youth	m	16.5 ± 1.5	1735.0 ± 336.1
Santone, et al., 2014	Soccer, elite, Serie C, third division, Italy	m	23.0 ± 3.0	1731.0 ± 380.0
Johnston, et al., 2015	Rugby, first division, Australia	m	16.6 ± 0.6	1700.0 ± 119.0
Teplan, et al., 2013	Soccer, elite, Czech Republic	m	18.3 ± 0.2	1700.0 ± 227.77
Aloui, et al., 2017	Soccer, amateur	m	22.9 ± 1.3	1696.0 ± 85.0
Taylor, et al., 2016	Soccer, semiprofessionals	m	25.0 ± 8.0	1691.0 ± 600.0
Risso, et al., 2017	Soccer, college-level, division 1, USA	f	20.1 ± 1.2	1690.0 ± 498.0
Aloui, et al., 2017	Soccer, amateur	m	22.9 ± 1.3	1688.0 ± 84.7
Sánchez-Sánchez, et al., 2016	Soccer, amateur	m	21.65 ± 3.1	1686.32 ± 306.16
Aloui, et al., 2017	Soccer, amateur	m	22.9 ± 1.3	1686.0 ± 87.5
Matzenbacher, et al., 2016	Futsal, U18 state level	m	17.2 ± 0.4	1684.0 ± 390.0
Castagna, et al., 2008	Basketball, regional level, Italy	m	16.8 ± 2.0	1678.0 ± 97.0
Till, et al., 2016	Rugby, academy league, UK	m	U19	1674.0 ± 455.0
Fanchini, et al., 2014	Soccer, professionals, fourth division,	m	17.0 ± 1.0	1668.0 ± 256.0

	Italy			
Lockie, et al., 2016	Soccer, college-level, first division, USA	f	20.2 ± 1.2	1666.7 ± 473.0
Moss, et al., 2015	Handball, top-elite, Europe	f	17.1 ± 1.1	1663.0 ± 327.0
Till, et al., 2014	Rugby, professionals, academy-level, UK	m	U20	1662.0 ± 397.0
Atkins, et al., 2006	Rugby, elite, English rugby league squad, first team players,UK	m	22.1 ± 5.0	1656.0 ± 403.0
Till, et al., 2016	Rugby, professional club academy, UK	m	17.1 ± 0.6	1650.0 ± 304.0
Till, et al., 2016	Rugby, professional club academy, UK	m	17.9 ± 0.2	1646.0 ± 476.0
Deprez, et al., 2014	Soccer, sub-elite, U17 second and fourth division	m	16.2 ± 0.6	1640.0 ± 475.0
Wylie, et al., 2013	Adults, recreational actice	m	22.0 ± 2.0	1636.0 ± 288.0
Ingebrigsten, et al., 2012	Soccer, sub-elite, third division, Norway	m	20.0 ± 3.0	1633.0 ± 476.0
Mohr, et al., 2014	Soccer, semiprofessionals, top league, central defenders, Faroe Islands	m	25.8 ± 4.1	1628.0 ± 367.0
Risso, et al., 2017	Soccer, college-level, first division, USA	f	20.4 ± 1.3	1628.0 ± 481.0
Sparks, et al., 2017	Soccer, university-level, South Africa	m	22.6 ± 2.5	1618.0 ± 429.0
Pareia-Blanco, et al., 2016	Soccer, highly-trained, professional soccer club	m	-	1611.0 ± 422.0
Sharpe, et al., 2016	Soccer, elite, international level, Serie League, Italy	m	22.47 ± 2.14	1606.0 ± 129.0
Lockie, et al., 2016	Soccer, college-level, first division, USA	f	19.63 ± 0.52	1600.0 ± 489.43
Afyon, et al., 2015	Soccer, professionals, second devision, Turkey	m	23.33 ± 3.84	1600.0 ± 280.84
Roe, et al., 2016	Gaelic football, sub-elite, first division, Ireland	m	20.5 ± 1.5	1585.4 ± 445.3
Attene, et al., 2015	Basketball, U17 national championship, Italy	m	16.0 ± 1.0	1583.0 ± 696.0
Haugen, et al., 2014	Soccer, highest junior division level, Norway	both	17.0 ± 1.0	1583.0 ± 669.0
Markovic, et al., 2011	Soccer, first division, Croatia	m	U17	1581.0 ± 390.0
Deprez, et al., 2015	Soccer, high level, goalkeeper	m	17.7 ± 0.6	1575.0 ± 213.0
Nyakayiru, et al., 2017	Soccer, amateur, second and third division, The Netherlands	m	23.0 ± 5.66	1574.0 ± 265.87
Atkins, et al., 2006	Rugby, semiprofessionals, second team, alliance players, UK	m	21.1 ± 4.7	1564.0 ± 415.0
Till, et al., 2016	Rugby, academy league, UK	m	U19	1560.0 ± 190.0
Pareja-Blanco, et al.,	Soccer, highly-trained, Morocco	m	24.3 ± 4.6	1558.0 ± 362.0

2016				
Till, et al., 2016	Rugby, U17 academy league, UK	m	U17	1553.0 ± 287.0
Yuki, et al., 2013	Cross-country skiing, moderatly-trained, high-school level, Japan	m	16.1 ± 0.9	1553.0 ± 451.0
Karsten, et al., 2016	Soccer, third division, Turkey	m	18.0 ± 1.0	1547.7 ± 315.1
Wong, et al., 2010	Soccer, professionals, highest level, Hong Kong	m	21.0 ± 4.36	1541.0 ± 226.66
Till, et al., 2016	Rugby, academy league, UK	m	U18	1535.0 ± 322.0
Cinarli, et al., 2016	Adults, inactive	both	-	1532.6 ± 770.7
Castillo, et al., 2017	Referees, soccer, elite, national level, assistance referees, Spain	m	29.6 ± 7.8	1531.43 ± 518.02
Macpherson, et al., 2015	Soccer, semiprofessionals, ninth tier, UK	m	25.0 ± 4.0	1523.0 ± 493.0
Macpherson, et al., 2015	Soccer, semiprofessionals, ninth tier, UK	m	27.0 ± 6.0	1520.0 ± 593.0
Shalfawi, et al., 2012	Soccer, well-trained, elite	m	16.3 ± 0.5	1520.0 ± 70.29
Till, et al., 2016	Rugby, academy league, UK	m	U20	1512.0 ± 299.0
Wong, et al., 2010	Soccer, professionals, highest division, Hong Kong	m	24.6 ± 6.71	1510.0 ± 335.41
Nakamura, et al., 2016	Futsal, professionals, top level, Brazil	m	27.94 ± 5.94	1506.7 ± 287.1
Lockie, et al., 2016	Soccer, college-level, first division, USA	f	19.6 ± 1.34	1504.0 ± 535.61
Cullen, et al., 2013	Gaelic Football, U18 A-level national championship, midfielders, Ireland	m	16.96 ± 0.7	1502.67 ± 327.45
Cullen, et al., 2013	Gaelic Football, U18 A-level national championship, defenders, Ireland	m	16.96 ± 0.7	1498.41 ± 356.05
Oliveira, et al., 2013	Futsal, high-level, national level, Brazil	m	24.3 ± 2.9	1491.0 ± 396.0
Till, et al., 2014	Rugby, professionals, Super League, first division, UK	m	U18	1490.0 ± 413.0
Teplan, et al., 2012	Soccer, worst U17 division, Czech Republic	m	16.6 ± 0.4	1488.57 ± 369.01
Vescovi, et al., 2016	Field hockey, national team, Canada	f	-	1480.0 ± 332.0
Coratella, et al., 2016	Soccer, recreational	m	20.1 ± 2.4	1476.0 ± 344.0
Till, et al., 2015	Rugby, U17 academy league, UK	m	16.74 ± 0.23	1475.0 ± 327.0
Till, et al., 2016	Rugby, U19 academy league, UK	m	U19	1475.0 ± 443.0
Kavaliauskas, et al., 2017	Soccer, semiprofessionals, Junior Football Association, UK	m	22.0 ± 8.0	1468.0 ± 409.0
Cobley, et al., 2011	Adults, recreational actice	m	24.7 ± 4.2	1466.7 ± 395.1

Darrall-Jones, et al., 2016	Rugby, U18 professional regional academy, backs, UK	m	16.9 ± 0.6	1466.6 ± 450.9
Cullen, et al., 2013	Gaelic Football, U18 A-level national championship, forwards, Ireland	m	16.96 ± 0.7	1466.42 ± 369.93
Till, et al., 2016	Rugby, U18 academy league, UK	m	U18	1464.0 ± 354.0
Nedrehagen, et al., 2015	Soccer, amateur, local level (male) and semiprofessional, national level (female)	both	20.3 ± 3.0	1455.0 ± 188.0
Till, et al., 2016	Rugby, U19 academy league, UK	m	U19	1443.0 ± 259.0
Shalfawi, et al., 2012	Soccer, well-trained, elite	m	16.3 ± 0.5	1440.0 ± 117.89
Furlan, et al., 2016	Rugby or australian Football	m	20.0 ± 2.0	1440.0 ± 4.17
Veale, et al., 2010	Australian Football, subelite, Australia	m	16.6 ± 0.5	1438.0 ± 335.0
Castillo, et al., 2016	Referees, soccer, regional level, Spain	m	29.1 ± 9.0	1437.65 ± 424.85
Michalsik, et al., 2014	Handball, elite, Premier League, first division, Denmark	f	25.9 ± 3.8	1436.0 ± 222.0
Till, et al., 2016	Rugby, U17 academy league, UK	m	U17	1436.0 ± 336.0
Invernizzi, et al., 2015	Karate, international level	both	18.0 ± 3.0	1428.0 ± 416.0
Karsten, et al., 2016	Soccer, third division, Turkey	m	19.0 ± 1.0	1427.7 ± 368.2
Matthys, et al., 2011	Handball, sub-elite, Belgium	m	16.6 ± 0.6	1426.0 ± 434.0
Karavelioglu, et al., 2014	Futsal, not described further	f	20.53 ± 1.7	1421.33 ± 287.59
Johnston, et al., 2015	Rugby, first division, Australia	m	16.6 ± 0.5	1420.0 ± 337.0
Vernillo, et al., 2012	Basketball, elite, U17 national team, Italy	m	U17	1412.0 ± 245.0
Smith, et al., 2016	Soccer, recreational	m	24.0 ± 0.4	1410.0 ± 354.0
Nedrehagen, et al., 2015	Soccer, amateur, local level (male) and semiprofessional, national level (female)	both	21.8 ± 2.6	1409.0 ± 336.0
Till, et al., 2016	Rugby, U18 academy league, UK	m	17.69 ± 0.26	1408.0 ± 281.0
Lockie, et al., 2016	Soccer, college-level, first division, USA	f	20.33 ± 10.3	1408.0 ± 342.23
Coratella, et al., 2016	Runners, basketball, futsal, triathlon athletes, tennis, recreational level	m	20.3 ± 1.9	1406.0 ± 486.0
Bruce, et al., 2016)	Netball, sub-elite, centre court, Australia	f	-	1400.0 ± 91.67
Shultz, et al., 2015	Recreational active adults (basketball, soccer, lacrosse, ultimate frisbee, tennis, volleyball, football, rugby)	m	20.3 ± 2.0	1393.0 ± 425.0

Till, et al., 2016	Rugby, professional club academy, UK	m	17.3 ± 0.7	1391.0 ± 223.0
Pareija-Blanco, et al., 2016	Soccer, highly-trained, professional soccer club	m	-	1390.0 ± 417.0
Darrall-Jones, et al., 2016	Rugby, professional regional academy, UK	m	19.3 ± 1.2	1384.0 ± 249.2
Thompson, et al., 2016	Team sport athletes (soccer, rugby, hockey), local teams	m	24.0 ± 4.0	1369.0 ± 505.0
Cinarli, et al., 2016	Adults, inactive	both	-	1364.8 ± 669.9
Iacono, et al., 2015	Handball, elite, national level, Israel	m	-	1364.4 ± 397.0
Castagna, et al., 2005	Referees, soccer, medium level, third division, Italy	m	27.8 ± 3.2	1360.0 ± 172.0
Ben Abdelkrim, et al., 2010	Basketball, elite, U18 national teams, Tunesia	m	17.5 ± 3.0	1355.0 ± 609.0
Till, et al., 2015	Rugby, academy league, UK	m	18.72 ± 0.2	1353.0 ± 352.0
Krustrup, et al., 2001	Referees, soccer, top-class, Superliga and first division, Denmark	m	-	1345.0 ± 175.36
Mohr, et al., 2015	Soccer, university-level	m	21.0 ± 3.16	1324.0 ± 122.0
Heaney, et al., 2012	Hockey, state level, Australia	f	22.3 ± 2.3	1320.0 ± 353.0
Eaton, et al., 2016	Football, subelite	m	22.0 ± 2.0	1320.0 ± 528.0
Serpiello, et al., 2011	Team sport, recreational	both	22.3 ± 4.1	1305.0 ± 709.0
Iacono, et al., 2015	Handball, elite, national level, Israel	m	-	1297.8 ± 300.0
Brophy-Williams, et al., 2011	Team sport athletes (australian football and hockey), well-trained, highest amateur level, Australia	m	20.9 ± 1.2	1296.0 ± 200.0
Soares-Caldeira, et al., 2014	Futsal, first division, Brazil	m	20.46 ± 4.66	1291.43 ± 363.48
Sant'anna, et al., 2016	Rugby, amateur, backs, Brazil	m	-	1283.3 ± 312.5
Scanlan, et al., 2012	Basketball, semiprofessionals	m	22.7 ± 6.1	1282.9 ± 361.9
Soares-Caldeira, et al., 2014	Futsal, first division, Brazil	m	25.07 ± 8.32	1280.0 ± 363.1
Cone, et al., 2012	Soccer, highly-trained, National Collegiate Athletic Association Division I, USA	f	-	1276.7 ± 306.3
Castagna, et al., 2005	Referees, soccer, low level, fourth division, Italy	m	24.8 ± 1.2	1272.0 ± 215.0
Cihan, et al., 2012	Soccer, prof, first division, goalkeeper, Turkey	m	26.0 ± 2.64	1266.0 ± 83.2
Nakamura, et al., 2016	Futsal, professionals, top U20 level, Brazil	m	19.13 ± 0.74	1264.0 ± 397.9

Nakamura,, et al., 2015	Futsal, elite, Paraná State Championship, Brazil	m	22.9 ± 4.2	1262.0 ± 330.6
Till, et al., 2016	Rugby, U19 academy league, UK	m	U19	1252.0 ± 262.0
Nicks, et al., 2009	Soccer, college-level, National Collegiate Athletic Association Division 1, USA	both	19.8 ± 0.9	1250.0 ± 351.2
Darrall-Jones, et al., 2015	Rugby, U21 professional regional academy, UK	m	19.0 ± 1.1	1243.0 ± 326.1
Johnston, et al., 2015	Rugby, first division, Australia	m	16.5 ± 0.6	1233.0 ± 304.0
Attene, et al., 2015	Basketball, U17 national championship, Italy	m	16.0 ± 1.0	1233.0 ± 663.0
Boullosa, et al., 2013	Futsal, professional, first division, Brazil	m	25.9 ± 5.1	1226.0 ± 282.0
Kilding, et al., 2016	Basketball, elite, national level and subelite and regional level, New Zealand	m	23.2 ± 5.6	1225.0 ± 470.0
Darrall-Jones, et al., 2015	Rugby, regional academy-level, UK	m	16.9 ± 0.5	1225.0 ± 373.8
Mujika, et al., 2009	Soccer, Super Liga, first division, Spain	f	23.1 ± 2.9	1224.0 ± 255.0
Alemdaroğlu, et al., 2012	Soccer, amateur	m	21.9 ± 2.5	1222.9 ± 287.0
Karavelioğlu, et al., 2014	Futsal, not described further	f	21.0 ± 1.46	1222.85 ± 212.65
Hermassi, et al., 2016	Handball, elite, highest national and international level	m	17.1 ± 0.3	1213.12 ± 71.83
Roe, et al., 2016	Gaelic football, sub-elite, first division, Ireland	m	17.0-19.0	1206.8 ± 327.3
Hamlin, et al., 2017	Rugby, well-trained non-professionals, New Zealand	m	20.3 ± 2.1	1200.0 ± 384.0
Clarke, et al., 2014	Rugby sevens, national team, Australia	f	25.0 ± 5.0	1200.0 ± 73.62
Raman, et al., 2014	Squash, national and international level, New Zealand	m	24.0 ± 8.0	1182.0 ± 414.0
Kavaliauskas, et al., 2017	Soccer, semiprofessionals, Junior Football Association, UK	m	23.0 ± 7.0	1164.0 ± 438.0
Darrall-Jones, et al., 2016	Rugby, regional academy-level, UK	m	18.9 ± 0.9	1142.9 ± 353.9
Aoki, et al., 2017	Basketball, professionals, national level, Brazil	m	27.8 ± 6.4	1120.0 ± 412.5
Nicks, et al., 2009	Soccer, college-level, National Collegiate Athletic Association Division 1, USA	both	19.9 ± 1.3	1116.9 ± 379.6
Dixon, et al., 2017	Adults, recreational actice	m	26.0 ± 4.0	1115.56 ± 519.66
Kilding, et al., 2016	Basketball, elite and subelite	m	21.2 ± 3.3	1114.0 ± 370.0
Roberts, et al., 2011	Recreational active adults	m	23.0 ± 2.0	1114.0 ± 327.0
Hermassi, et al., 2016	Handball, elite, highest national and international level	m	17.3 ± 0.5	1109.44 ± 42.19

Hamlin, et al., 2017	Rugby, well-trained non-professionals	m	22.0 ± 4.1	1100.0 ± 426.0
Attene, et al., 2016	Basketball, U17 international championship, Italy	both	16.1 ± 0.9	1098.0 ± 274.0
Martin, et al., 2017	Team sport athletes (australian football, basketball, hockey, netball), state level, Australia	f	16.4 ± 1.1	1097.0 ± 441.0
Hasegawa, et al., 2015	Soccer, Aichi Division 1, regional level, defenders, Japan	f	19.5 ± 1.1	1095.0 ± 324.2
Martin, et al., 2017	Team sport athletes (australian football, basketball, hockey, netball), state level, Australia	f	16.4 ± 1.1	1093.0 ± 299.0
Attene, et al., 2016	Basketball, U17 international championship, Italy	both	16.2 ± 0.9	1092.0 ± 238.0
Johnston, et al., 2015	Rugby, third division, Australia	m	16.3 ± 0.4	1089.0 ± 188.0
Darrall-Jones, et al., 2016	Rugby, U18 regional academy-level, forwards, UK	m	16.9 ± 0.5	1080.0 ± 240.0
Vernillo, et al., 2012	Basketball, subelite, regional U17 team, Italy	m	U17	1078.0 ± 565.0
Cullen, et al., 2013	Gaelic Football, U18 A-level national championship, goalkeepers, Ireland	m	16.96 ± 0.7	1070.77 ± 401.71
Vescovi, et al., 2016	Field hockey, U17 national team, Canada	f	U17	1068.0 ± 220.0
Flatt, et al., 2016	Soccer, college-level, National Association for Intercollegiate Athletics, USA	f	22.0 ± 2.3	1066.6 ± 243.8
Martínez-Lagunas, et al., 2014	Soccer, second division, Germany	f	21.5 ± 3.4	1051.0 ± 399.0
Thomas, et al., 2006	Cricket, state-level	m	16.7 ± 1.2	1049.0 ± 285.0
Boullosa, et al., 2012	Referees, soccer, sub-elite, regional level, third division, Spain	m	26.0 ± 5.0	1044.0 ± 304.0
Bruce, et al., 2016	Netball, sub-elite, defenders, Australia	f	-	1040.0 ± 72.87
Shalfawi, et al., 2013	Soccer, well-trained elite, upper league, Norway	f	-	1025.0 ± 274.0
Thomas, et al., 2006	Adults, recreational actice	m	24.4 ± 6.0	1010.0 ± 419.0
Scanlan, et al., 2014	Basketball, regional and state level, Australia	m	24.9 ± 5.5	995.7 ± 464.3
Rowat, et al., 2017	Soccer, academy-level, Singapore	m	17.4 ± 0.9	972.7 ± 478.4
Bruce, et al., 2016	Netball, sub-elite, goalers, Australia	f	-	960.0 ± 55.74
Sirotic, et al., 2007	Team sport athletes (touch football, soccer, netball, hockey), moderately-trained, regional level	m	20.9 ± 1.8	958.0 ± 368.0
Roberts, et al., 2011	Adults, recreational actice	m	21.0 ± 3.0	950.0 ± 432.0

Johnston, et al., 2015	Rugby, third division, Australia	m	16.5 ± 0.6	922.0 ± 227.0
Shalfawi, et al., 2013	Soccer, well-trained elite, upper league, Norway	f	-	920.0 ± 293.0
Schmitz, et al., 2017	Adults, moderately-trained	f	23.11 ± 2.56	891.11 ± 279.43
Shultz, et al., 2015	Recreational active adults (basketball, soccer, ultimate frisbee, tennis, volleyball, rugby)	f	20.5 ± 2.3	847.0 ± 313.0
Thomas, et al., 2006	Hockey, state-level	f	19.4 ± 0.7	840.0 ± 280.0
Idrizović, et al., 2013	Soccer, national team, Montenegro	f	21.87 ± 1.98	836.66 ± 255.96
Borges, et al., 2017	Soccer, recreational	m	16.3 ± 0.6	836.0 ± 192.71
Mujika, et al., 2009	Soccer, Primera National, second division, Spain	f	17.3 ± 1.6	826.0 ± 160.0
Hasegawa, et al., 2015	Soccer, Aichi Division 1, regional level, midfielders, Japan	f	19.5 ± 1.0	820.0 ± 316.7
Vernillo, et al., 2012	Basketball, nonathletic	m	U17	815.0 ± 277.0
Cinarli, et al., 2016	Adults, inactive	both	-	804.1 ± 425.1
Idrizovic, et al., 2014	Soccer, national team, Montenegro	f	18.0-26.0	801.42 ± 253.09
Sant'anna, et al., 2016	Rugby, amateur, forwards, Brazil	m	-	792.0 ± 277.6
Berdejo-del-Fresno, et al., 2013	Basketball, U20 national team, UK	f	18.47 ± 0.47	790.77 ± 289.44
Johnston, et al., 2015	Rugby, third division, Australia	m	16.7 ± 0.6	785.0 ± 155.0
Veale, et al., 2010	Adults, inactive	m	16.6 ± 0.5	774.0 ± 358.0
Hasegawa, et al., 2015	Soccer, Aichi Division 1, regional level, forwards, Japan	f	19.2 ± 0.8	760.0 ± 215.4
Jones, et al., 2016	Rugby, elite, international-level, backs, UK	f	23.5 ± 4.1	728.0 ± 154.0
Cal Abad, et al., 2016	Basketball	f	16.92 ± 1.07	720.0 ± 212.94
Berdejo-del-Fresno, et al., 2013	Basketball, elite, first division, UK	f	20.5 ± 2.31	701.54 ± 265.14
Zhang, et al., 2013	Recreational active adults	both	25.0 ± 4.0	654.0 ± 311.0
Scanlan, et al., 2012	Basketball, recreational	m	26.6 ± 4.0	635.6 ± 296.9
Jones, et al., 2016	Rugby elite, international-level, forwards, UK	f	26.3 ± 6.4	610.0 ± 292.0
Hasegawa, et al., 2015	Soccer, Aichi Division 1, regional level, goalkeepers, Japan	f	19.0 ± 0.0	600.0 ± 169.7
Purkhus, et al., 2016	Volleyball, elite, top level, Faroe Islands	f	18.0 ± 4.0	556.0 ± 206.0

Dinardi, et al., 2017	Rugby, amateur, Brazil	m	26.5 ± 4.7	528.0 ± 159.4
Boullosa, et al., 2012	Referees, soccer, sub-elite, regional level, third division, Spain	f	22.0 ± 3.0	408.0 ± 125.0
Lim, et al., 2012	Adults, inactive	m	16.72 ± 1.24	400.0 ± 24.15

Supplemental table 2: Yo-Yo Intermittent Recovery Level 2 test (YYIR2)

Study	Subgroup/ level	Sex	Age, y (±SD)	Result, m (±SD)
Kelly, et al., 2017	Gaelic football, elite, first division, half-forward	m	26.6 ± 6.0	1840.0 ± 335.0
Kelly, et al., 2017	Gaelic football, elite, first division, midfielder	m	26.6 ± 6.0	1792.0 ± 175.0
Bouaziz, et al., 2016	Rugby sevens, national team, Tunisia	m	23.8 ± 0.8	1730.0 ± 314.8
Shovlin, et al., 2017	Gaelic football, elite, Ireland	m	26.6 ± 6.0	1587.0 ± 298.0
Kelly, et al., 2017	Gaelic football, elite, first division, half-back	m	26.6 ± 6.0	1580.0 ± 305.0
Born, et al., 2017	Team sport athletes, amateur	m	22.5 ± 3.4	1469.88 ± 299.78
Kelly, et al., 2017	Gaelic football, elite, first division, full-back	m	26.6 ± 6.0	1424.0 ± 209.0
Bassini, et al., 2013	Soccer, elite professionals, major league team, Brazil	m	-	1367.0 ± 933.38
Pivovarniček, et al., 2013	Soccer, elite, U21 national team, defenders, Slovakia	m	U22	1345.0 ± 126.0
Pivovarniček, et al., 2013	Soccer, elite, U21 national team, midfielders, Slovakia	m	U23	1343.0 ± 352.0
Jamurtas, et al., 2015	Soccer, sub-elite	m	22.2 ± 1.3	1341.7 ± 193.4
Pivovarniček, et al., 2013	Soccer, elite, U21 national team, forwards, Slovakia	m	U24	1304.0 ± 288.0
Stevens, et al., 2016	Soccer, professionals, first division, second division, Championsleague, The Netherlands	m	21.0 ± 3.0	1300.0 ± 210.0
Lockie, et al., 2016	Soccer, college-level, first division, USA	m	21.2 ± 1.32	1210.0 ± 379.39
Kelly, et al., 2017	Gaelic football, elite, first division, full-forward	m	26.6 ± 6.0	1200.0 ± 202.0
Cholewa, et al., 2015	Soccer, college-level, third division, USA	m	18.0-22.0	1200.0 ± 513.0
Saunders, et al., 2012	Soccer, amateur, lower division, UK	m	22.0 ± 4.0	1185.0 ± 216.0
Saunders, et al., 2012	Soccer, amateur, lower division, England	m	22.0 ± 4.0	1163.29 ± 180.0
Stevens, et al., 2016	Soccer, professional, U17/U19 teams, The Netherlands	m	17.0 ± 1.0	1147.0 ± 244.0
Veugelers, et al., 2016	Australian rules football, elite, Australia	m	23.0 ± 4.0	1141.0 ± 318.0
Karavelioğlu, et al., 2014	Soccer, U19 amateur league	m	17.58 ± 0.66	1123.33 ± 222.68
Hogarth, et al., 2015	Tag Football, highest-standard, inside players, Australia	m	26.0 ± 3.0	1115.0 ± 406.0
Gatterer, et al., 2015	Soccer, amateur	m	-	1109.0 ± 209.0
Mohr, et al., 2016	Soccer, elite semiprofessionals	m	26.2 ± 5.67	1106.0 ± 257.18

Saunders, et al., 2012	Soccer, amateur, lower division, UK	m	-	1093.0 ± 148.0
Mooney, et al., 2011	Australian football, elite	m	22.3 ± 3.3	1060.0 ± 176.0
Krustrup, et al., 2006	Soccer, elite, international level, Scandinavia	m	17.0-35.0	1059.0 ± 207.06
Lockie, et al., 2016	Soccer, college-level, first division, USA	m	20.53 ± 1.5	1048.78 ± 365.91
Joo, et al., 2016	Soccer, well-trained, university-level, South Korea	m	22.0 ± 2.0	1040.0 ± 291.8
McLean, et al., 2015	Australian football, amateur	m	-	1040.0 ± 312.0
Ueda, et al., 2011	Soccer, elite, college-level, Japan	m	20.8 ± 0.4	1035.0 ± 147.5
Mohr, et al., 2014	Soccer, semiprofessionals, top league, Faroe Islands	m	25.8 ± 4.1	1034.0 ± 211.0
Krustrup, et al., 2006	Soccer, first division, Scandinavia	m	17.0-37.0	1033.0 ± 174.28
Mohr, et al., 2013	Soccer, elite, top league, Scandinavia	m	26.7 ± 4.36	1032.0 ± 183.07
Gatterer, et al., 2015	Soccer, amateur	m	-	1029.0 ± 273.0
Mooney, et al., 2013	Australian Football, elite	m	22.6 ± 3.2	1029.0 ± 185.0
Mooney, et al., 2013	Australian Football, elite professionals	m	22.3 ± 3.3	1028.0 ± 190.0
Hogarth, et al., 2015	Tag football, highest standard, Australia	m	23.4 ± 3.3	1023.0 ± 113.0
Iaia, et al., 2017	Soccer, subelite	m	17.0 ± 1.0	1016.0 ± 217.0
Iaia, et al., 2017	Soccer, subelite	m	17.0 ± 1.0	1000.0 ± 169.0
Iaia, et al., 2015	Soccer, professionals	m	18.5 ± 1.0	989.0 ± 226.0
Ingebrigsten, et al., 2012	Soccer, elite, first division, Denmark	m	25.0± 5.0	977.0 ± 205.0
Michalsik, et al., 2015	Handball, elite, first division, Denmark and Champions League and national teams, Europe, wing players	m	26.4 ± 3.1	975.0 ± 123.0
McLean, et al., 2015	Australian Football, amateur	m	-	973.0 ± 185.0
Wells, et al., 2012	Soccer, professionals, UK	m	23.2 ± 2.4	966.0 ± 153.0
Rampinini, et al., 2010	Soccer, professionals	m	25.0 ± 4.0	958.0 ± 99.0
Ingebrigsten, et al., 2012	Soccer, elite, first division, Norway	m	26.0 ± 7.0	941.0 ± 278.0
Christensen, et al., 2011	Soccer, elite, second division (third best league), Denmark	m	23.4 ± 3.5	937.0 ± 56.0

Thomassen, et al., 2010	Soccer, elite, highly trained	m	-	937.0 ± 148.16
Hogarth, et al., 2015	Tag Football, highest-standard, outside players, Australia	m	22.0 ± 2.0	930.0 ± 269.0
Iaia, et al., 2015	Soccer, professionals	m	18.5 ± 1.0	927.0 ± 185.0
Ueda, et al., 2011	Soccer, non-elite, college-level	m	20.1 ± 0.6	919.0 ± 119.3
Pivovarniček, et al., 2013	Soccer, elite, U21 national team, goalkeepers, Slovakia	m	U21	900.0 ± 85.0
Michalsik, et al., 2015	Handball, elite, first division, Denmark and Champions League and national teams, Europe, backcourt players	m	26.4 ± 3.1	897.0 ± 108.0
Wells, et al., 2014	Soccer, professionals, well trained	m	-	896.0 ± 37.0
Lockie, et al., 2016	Soccer, college-level, first division, USA	m	19.29 ± 1.1	893.33 ± 250.01
Wells, et al., 2014	Soccer, professionals, well trained	m	-	891.0 ± 46.0
Thomassen, et al., 2010	Soccer, elite, highly trained	m	23.4 ± 3.39	880.78 ± 154.91
Stein, et al., 2015	Australian football, recreational, first division, Australia	m	24.9 ± 4.9	880.0 ± 260.0
OliveiraMatta, et al., 2015	Soccer, regional-level, Brazil	m	16.22 ± 0.14	874.8 ± 573.61
Gunnarsson, et al., 2012	Soccer, subelite, second division, Denmark	m	23.9 ± 0.1	862.0 ± 227.15
Nakamura, et al., 2012	Soccer, semiprofessionals, third division and college-level, regional league, Japan	m	22.7 ± 2.4	860.0 ± 120.0
Nakamura, et al., 2012	Soccer, semiprofessionals, third division and college-level, regional league, Japan	m	23.3 ± 2.8	855.4 ± 221.8
Stevens, et al., 2016	Soccer, amateur, the fifth and sixth amateur level, The Netherlands	m	26.0 ± 4.0	849.0 ± 264.0
Christensen, et al., 2011	Soccer, elite, second division (third best league), Denmark	m	-	845.0 ± 160.0
Thomassen, et al., 2010	Soccer, elite, highly trained	m	-	845.0 ± 159.2.0
Chuman, et al., 2011	Soccer, first division, Japan	m	U17	843.0 ± 106.0
Chan, et al., 2016	Soccer, professionals, first division, Hong Kong	m	25.6 ± 5.0	840.5 ± 263.0
Wells, et al., 2012	Soccer, amateur, local amateur league, UK	m	21.1 ± 1.6	840.0 ± 156.0
Michalsik, et al., 2015	Handball, elite, first division, Denmark and Champions League and national teams, Europe, pivots	m	26.4 ± 3.1	827.0 ± 264.0
Michalsik, et al., 2015	Handball, elite, first division, Denmark and Champions League and national teams, Europe, goalkeeper	m	26.4 ± 3.1	807.0 ± 205.0
Kvorning, et al., 2017	Handball, professionals, national team,	m	29.5 ± 4.0	797.0 ± 175.0

	Denmark			
Hogarth, et al., 2015	Tag football, highest standard, Australia	m	24.3 ± 1.9	775.0 ± 137.0
Krustrup, et al., 2006	Soccer, second division, Scandinavia	m	17.0-36.0	771.0 ± 100.7
Ingebrigsten, et al., 2012	Soccer, subelite, third division, Denmark	m	24.0 ± 5.0	769.0 ± 199.0
McGawley, et al., 2013	Soccer, professionals and semiprofessionals, first division, Sweden	m	23.0 ± 5.0	769.0 ± 105.0
Nogueira, et al., 2016	Futsal, high level, Brazil	m	28.4 ± 6.6	762.67 ± 211.37
Miloski, et al., 2014	Futsal, professionals, high-level, national league, Brazil	m	26.3 ± 4.9	750.0 ± 150.6
Young, et al., 2005	Australian rules football, elite, Australia	m	22.7 ± 3.4	747.0 ± 128.0
McGawley, et al., 2013	Soccer, professionals and semiprofessionals, first division, Sweden	m	23.0 ± 4.0	729.0 ± 202.0
Nakamura, et al., 2012	Soccer, semiprofessionals, third division and college-level, regional league, Japan	m	22.0 ± 0	720.0 ± 203.9
Yuki, et al., 2013	Cross-country skiing, elite, high-school level, Japan	m	16.9 ± 0.3	715.0 ± 271.0
Buchheit, et al., 2013	Australian football, professionals, Australia	m	-	711.0 ± 109.0
Thomas, et al., 2006	Australian football, elite	m	23.5 ± 3.5	708.0 ± 157.0
Buchheit, et al., 2013	Australian football, professionals, Australia	m	-	705.0 ± 105.0
Marriott, et al., 2015	Team sport athletes, subelite	m	20.8 ± 1.4	696.0 ± 185.0
Mohr, et al., 2016	Soccer, university-level	m	19.0 ± 4.24	693.0 ± 156.0
Mohr, et al., 2016	Soccer, university-level	m	19.0 ± 4.24	686.5 ± 254.56
Krustrup, et al., 2006	Adults, habitually active	m	-	682.0 ± 250.41
Mohr, et al., 2016	Soccer, university-level	m	19.0 ± 4.24	680.0 ± 204.0
Rebelo, et al., 2010	Soccer, professionals, first division, South Africa	m	25.0 ± 4.0	664.0 ± 28.87
Krustrup, et al., 2015	Ultimate frisbee, club-level, UK	m	20.5 ± 0.8	657.0 ± 225.0
Lockie, et al., 2016	Soccer, college-level, first division, USA	f	21.6 ± 0.89	648.0 ± 181.99
Kustrup, et al., 2015	Trained athletes (middle distance running, team sports, triathlon)	m	23.0 ± 3.61	646.0 ± 165.86
Fanchini, et al., 2014	Soccer, professionals, fourth division, Italy	m	17.0 ± 1.0	645.0 ± 144.0
Stevens, et al., 2016	Soccer, professionals, BeNe League, first division, The Netherlands	f	24.0 ± 4.0	634.0 ± 155.0

Ingebrigsten, et al., 2014	Soccer, high-level, first division, second division, third division, Norway	m	22.0 ± 5.0	613.0 ± 174.0
Rampinini, et al., 2010	Soccer, amateur	m	25.0 ± 5.0	613.0 ± 125.0
Owen, et al., 2013	Soccer, semiprofessionals	m	22.2 ± 3.1	612.0 ± 55.0
Owen, et al., 2013	Soccer, semiprofessionals	m	22.2 ± 3.1	609.0 ± 47.0
Owen, et al., 2013	Soccer, semiprofessionals	m	22.2 ± 3.1	606.0 ± 59.0
Krustrup, et al., 2006	Adults, habitually active	m	22.0-30.0	591.0 ± 155.04
Ingebrigsten, et al., 2012	Soccer, subelite, third division, Norway	m	20.0 ± 3.0	571.0 ± 155.0
Rebelo, et al., 2010	Soccer, Vodacom cup, South Africa	m	19.0 ± 1.9	568.0 ± 19.49
Ingebrigtsen, et al., 2013	Soccer, elite, high-level	m	16.9 ± 0.4	559.0 ± 133.0
Young, et al., 2005	Australian football, elite, Australia	m	22.6 ± 2.9	547.0 ± 61.0
Brocherie, et al., 2015	Field hockey, elite, first division, Belgium, Spain, The Netherlands	m	26.5 ± 4.4	543.0 ± 159.0
Brocherie, et al., 2015	Field hockey, elite, first division, Belgium, Spain, The Netherlands	m	25.3 ± 4.2	540.0 ± 126.0
Lockie, et al., 2016	Soccer, college-level, first division, USA	f	20.2 ± 1.2	533.3 ± 164.1
Nunes, et al., 2014	Basketball, elite, national team, Brazil	f	26.0 ± 5.0	527.0 ± 146.0
Mohr, et al., 2015	Soccer, university-level	m	21.0 ± 3.16	524.0 ± 52.0
Brocherie, et al., 2015	Field hockey, elite, first division, Belgium, Spain, The Netherlands	m	27.6 ± 4.8	520.0 ± 165.0
Mohr, et al., 2007	Adults, normally active	m	26.7 ± 4.16	520.0 ± 132.27
Lockie, et al., 2016	Soccer, college-level, first division, USA	f	19.6 ± 1.34	520.0 ± 117.76
Lockie, et al., 2016	Soccer, college-level, first division, USA	f	19.63 ± 0.52	508.57 ± 159.52
Lollo, et al., 2014	Soccer, professionals, first division, Brazil	m	18.0 ± 0.8	496.0 ± 46.9
Skovgaard, et al., 2014	Endurance runners, moderatly-trained	m	-	491.0 ± 225.17
Mohr, et al., 2007	Adults, normally active	m	24.6 ± 1.59	483.0 ± 161.39
Stein, et al., 2015	Australian football, recreational, second division, Australia	m	27.3 ± 5.2	476.0 ± 83.0
Lockie, et al., 2016	Soccer, college-level, first division, USA	f	20.33 ± 10.3	464.0 ± 171.11
Inness, et al., 2016	Australian football, semiprofessionals, regional league, Australia	m	-	454.0 ± 113.0

Ingebrigtsen, et al., 2013	Soccer, elite, high-level	m	16.9 ± 0.8	444.0 ± 121.0
Yuki, et al., 2013	Cross-country skiing, moderatly-trained, high-school level, Japan	m	16.1 ± 0.9	444.0 ± 168.0
Iaia, et al., 2008	Endurance runners, moderatly-trained	m	33.4 ± 5.03	440.0 ± 164.05
Rogan, et al., 2015	Soccer, amateur, Germany	m	25.1 ± 4.4	438.33 ± 149.66
Boer, et al., 2016	Soccer, sub-elite Football South Africa	m	22.0 ± 1.3	435.3 ± 174.9
De Souza, et al., 2006	Handball, Londrina country Paraná, Brazil	m	20.0-32.0	429.1 ± 130.3
Skovgaard, et al., 2014	Endurance runners, moderatly-trained	m	-	429.0 ± 279.0
Mara, et al., 2015	Soccer, elite, national-level	f	-	425.0 ± 122.0
Leme, et al., 2015	Handball, elite, national-level and international-level, Brazil	m	26.0 ± 4.6	418.0 ± 120.0
Boer, et al., 2016	Soccer, sub-elite Football South Africa	m	22.2 ± 2.6	415.4 ± 160.4
Brocherie, et al., 2015	Field hockey, elite, first division, Belgium, Spain, The Netherlands	m	22.3 ± 4.6	413.0 ± 89.0
Inness, et al., 2016	Australian football, semiprofessionals, regional league, Australia	m	-	412.0 ± 136.0
Morton, et al., 2009	Adults, recreationally active	m	21.0 ± 1.0	411.0 ± 130.0
Stein, et al., 2015	Australian football, recreational, third division, Australia	m	27.4 ± 3.6	408.0 ± 128.0
Iaia, et al., 2008	Endurance runners, moderatly-trained	m	-	405.0 ± 177.27
Morton, et al., 2009	Adults, recreationally active	m	20.0 ± 1.0	40.00 ± 95.0
Boer, et al., 2016	Soccer, sub-elite Football South Africa	m	22.1 ± 1.6	399.4 ± 104.8
Morton, et al., 2009	Adults, recreationally active	m	20.0 ± 1.0	393.0 ± 132.0
Rebelo, et al., 2010	Soccer, amateur, university-level, South Africa	m	21.0 ± 2.6	376.0 ± 17.27
Roberts, et al., 2011	Adults, recreationally active	m	21.0 ± 3.0	375.0 ± 102.0
Sampaio, et al., 2014	Basketball, national level, Portugal	m	17.4 ± 1.1	368.6 ± 46.6
Rogan, et al., 2015	Soccer, amateur, Germany	m	25.3 ± 4.6	358.33 ± 131.61
Roberts, et al., 2011	Adults, recreationally active	m	23.0 ± 2.0	337.0 ± 107.0
Thomas, et al., 2006	Adults, recreationally active	m	24.4 ± 6.0	322.0 ± 110.0
Purkhús, et al., 2016	Volleyball, elite, first division, Faroe Islands	f	20.0 ± 7.0	193.0 ± 62.0

Purkhús, et al., 2016	Volleyball, elite, first division, Faroe Islands	f	18.0 ± 4.0	191.0 ± 43.0

Supplemental table 3: Yo-Yo Intermittent Endurance Level 1 test (YYIE1)

Study	Subgroup/ level	Sex	Age, y (±SD)	Result, m (±SD)
Akashi, et al., 2014	Handball, university-level	m	19.4 ± 0.8	3720.0 ± 566.77
Julian, et al., 2017	Soccer, high level, sub-elite, regional-level and second division, Germany	f	19.0 ± 4.0	3289.0 ± 801.0
Castagna, et al., 2006	Soccer, regional-level, Italy	m	16.6 ± 0.8	3044.0 ± 442.0
Deliceoglu, et al., 2013	Soccer, elite, professionals, Turkey	m	23.02 ± 4.49	2040.2 ± 458.66
Rowan, et al., 2012	Soccer, university-level, third division, USA	f	19.5 ± 0.93	1857.0 ± 423.0
Metaxas, et al., 2005	Soccer, elite, national team, Greece	m	18.1 ± 1.0	1625.2 ± 297.47
Rowan, et al., 2012	Soccer, university-level, third division; USA	f	19.5 ± 0.93	1473.0 ± 494.0
Seidelin, et al., 2017	Adults, inactive	f	48.0 ± 3.0	1098.0 ± 702.0
Seidelin, et al., 2017	Adults, inactive	f	52.0 ± 4.0	713.0 ± 356.0
Flotum, et al., 2016	Adults, inactive	f	41.7 ± 7.49	544.0 ± 337.23
Flotum, et al., 2016	Adults, inactive	f	43.6 ± 8.6	540.0 ± 331.78
Mohr, et al., 2014	Adults, inactive	f	43.0 ± 13.42	458.0 ± 192.3
Krustrup, et al., 2016	Adults, inactive	f	45.0 ± 4.0	447.0 ± 151.0
Mohr, et al., 2014	Adults, inactive	f	45.0 ± 13.75	420.0 ± 206.22
Krustrup, et al., 2016	Adults, inactive	f	45.0 ± 6.0	413.0 ± 187.0

Supplemental table 4: Yo-Yo Intermittent Endurance Level 2 test (YYIE2)

Study	Subgroup/ level	Sex	Age, y (±SD)	Result, m (±SD)
Gibson, et al., 2013	Soccer, elite, academy-level	m	18.5 ± 0.7	2892.0 ± 484.0
Bradley, et al., 2011	Soccer, elite, Denmark	m	-	2766.0 ± 333.0
Bradley, et al., 2011	Soccer, elite, Denmark	m	-	2760.0 ± 416.0
Bradley, et al., 2011	Soccer, elite, UK	m	U19	2564.0 ± 554.0
Bradley, et al., 2011	Soccer, elite, UK	m	U19	2560.0 ± 574.0
Bradley, et al., 2011	Soccer, elite, UK	m	-	2501.0 ± 427.0
Bradley, et al., 2013	Soccer, Premier League, first division, UK	m	26.0 ± 5.0	2364.0 ± 478.0
Bradley, et al., 2013	Soccer, English Football League Championship, second division, UK	m	25.0 ± 5.0	2268.0 ± 567.0
Silva, et al., 2011	Soccer, elite professionals, Portugal	m	25.7 ± 4.6	2250.0 ± 296.4.0
Bradley, et al., 2013	Soccer English Football League One, third division, UK	m	27.0 ± 6.0	2226.0 ± 432.0
Gibson, et al., 2013	Soccer, elite, academy-level	m	16.4 ± 0.5	2139.0 ± 650.0
Krustrup, et al., 2015	Soccer, recreational	m	25.0 ± 7.0	2027.0 ± 298.0
Silva, et al., 2013	Soccer, elite professionals, Portugal	m	25.7 ± 4.6	1776.0 ± 358.0
Bradley, et al., 2014	Soccer, elite, Championsleague, Europe	f	23.0 ± 2.0	1774.0 ± 523.0
Randers, et al., 2013	Soccer, elite, national team, Denmark	f	24.4 ± 4.0	1772.0 ± 508.0
Povoas, et al., 2014	Handball, elite, top-level, Portugal	m	26.0 ± 3.0	1686.0 ± 580.0
Aziz, et al., 2005	Soccer, U18 national team	m	17.7 ± 0.4	1676.0 ± 314.0
Rampinini, et al., 2007	Soccer, amateur	m	24.5 ± 4.1	1606.0 ± 281.0
Bradley, et al., 2014	Soccer elite, U20 national team, Europe	f	19.0 ± 1.0	1490.0 ± 447.0
Rebelo, et al., 2013	Soccer, elite, U19 first division, midfielder, Portugal	m	18.3 ± 0.6	1464.0 ± 392.0
Rebelo, et al., 2013	Soccer, elite, U19 first division, fullback, Portugal	m	18.3 ± 0.6	1433.0 ± 546.0
Massuca, et al., 2014	Handball, elite, first division, Portugal	m	25.2 ± 4.8	1410.29 ± 469.93
Rebelo, et al., 2013	Soccer, elite, U19 first division, central defender, Portugal	m	18.3 ± 0.6	1354.0 ± 331.0
Rebelo, et al., 2013	Soccer, elite, U19 first division, forward, Portugal	m	18.3 ± 0.6	1328.0 ± 415.0

Brito, et al., 2010	Soccer	m	18.3 ± 0.5	1280.0 ± 358.0
Krustrup, et al., 2010	Soccer, elite, first division, Denmark	m	23.0	1265.0 ± 637.85
Bradley, et al., 2014	Soccer, highest tier, Europe	f	22.0 ± 3.0	1261.0 ± 449.0
Ascensão, et al., 2011	Soccer, national league	m	18.3 ± 0.8	1217.1 ± 409.1
Brito, et al., 2010	Soccer	m	16.6 ± 0.4	1132.0 ± 367.0
Rebelo, et al., 2013	Soccer, non-elite, regional-level, central defender, Portugal	m	18.1 ± 0.6	1091.0 ± 396.0
Rebelo, et al., 2013	Soccer, non-elite, regional-level, fullback, Portugal	m	17.9 ± 0.6	1076.0 ± 298.0
Rebelo, et al., 2013	Soccer, non-elite, regional-level, midfielder, Portugal	m	18.1 ± 0.6	1043.0 ± 346.0
Rebelo, et al., 2013	Soccer, non-elite, regional-level, forward, Portugal	m	18.1 ± 0.5	1022.0 ± 353.0
Massuca, et al., 2015	Handball top elite and moderate elite and sub-elite and moderatly-trained, back left/right, Portugal	m	-	1013.75 ± 428.36
Dixon, et al., 2014	Referees, Futsal, National and Super Futsal League	m	36.9 ± 9.9	1002.0 ± 325.1
Bradley, et al., 2014	Soccer, lowest tier, Europe	f	23.0 ± 4.0	994.0 ± 373.0
Rebelo, et al., 2013	Soccer, elite, U19 first division, goalkeeper, Portugal	m	18.2 ± 0.6	992.0 ± 214.0
Rebelo, et al., 2011	Referees, futsal, elite, Portugal	m	33.0 ± 5.1	975.0 ± 237.0
Massuca, et al., 2015	Handball top elite and moderate elite and sub-elite and moderatly-trained, wing, Portugal	m	-	964.88 ± 424.47
Massuca, et al., 2015	Handball top elite and moderate elite and sub-elite and moderatly-trained, back center, Portugal	m	-	958.4 ± 486.28
Dixon, et al., 2014	Referees, Futsal, second division	both	32.0 ± 11.5	927.0 ± 304.0
Massuca, et al., 2015	Handball top elite and moderate elite and sub-elite and moderatly-trained, goalkeeper, Portugal	m	-	842.86 ± 607.43
Matta, et al., 2014	Soccer, Brazil	m	16.1 ± 0.6	819.5 ± 336.4
Massuca, et al., 2015	Handball top elite and moderate elite and sub-elite and moderatly-trained, pivot, Portugal	m	-	790.0 ± 477.34
Randers, et al., 2010	Adults, inactive	m	20.0-43.0	778.0 ± 351.01
Massuca, et al., 2014	Handball, subelite, second or third division, Portugal	m	26.2 ± 4.9	770.19 ± 353.72
Randers, et al., 2010	Adults, inactive	m	20.0-43.0	726.0 ± 325.43

Krustrup, et al., 2015	Adults, inactive	m	30.0 ± 6.0	665.0 ± 271.0
Krustrup, et al., 2010	Adults, inactive	m	20.0-43.0	662.0 ± 252.98
Krustrup, et al., 2010	Adults, inactive	m	20.0-43.0	655.0 ± 128.17
Rebelo, et al., 2013	Soccer, non-elite, regional-level, goalkeeper, Portugal	m	17.9 ± 0.4	647.0 ± 247.0
Krustrup, et al., 2010	Adults, inactive	m	20.0-43.0	569.0 ± 269.79
Bangsbo, et al., 2010	Adults, inactive	f	19.0-47.0	274.0 ± 119.73
Bangsbo, et al., 2010	Adults, inactive	f	19.0-47.0	246.83
Randers, et al., 2013	Adults, inactive	f	29.3 ± 5.7	234.0 ± 66.0
Krustrup, et al., 2010	Adults, inactive	f	40.0 ± 3.0	234.0 ± 52.92
Randers, et al., 2013	Adults, inactive	f	27.0 ± 6.5	229.0 ± 60.0
Krustrup, et al., 2010	Adults, inactive	f	40.0 ± 2.0	225.0 ± 67.88
Bangsbo, et al., 2010	Adults, inactive	f	19.0-47.0	193.43

SUBLEMENTAL REFERENCES

Attene,G., Nikolaidis,P. T., Bragazzi,N. L., Dello Iacono,A., Pizzolato,F., Zagatto,A. M., et al., (2016).Repeated Sprint Ability in Young Basketball Players (Part 2): The Chronic Effects of Multidirection and of One Change of Direction Are Comparable in Terms of Physiological and Performance Responses. *Front. Physiol.* 7, 262.

Born,D. P., Kunz,P. and Sperlich,B. (2017).Reliability and validity of an agility-like incremental exercise test with multidirectional change-of-direction movements in response to a visual stimulus. *Physiol. Rep.* 5.

Brophy-Williams,N., Landers,G. and Wallman,K. (2011).Effect of immediate and delayed cold water immersion after a high intensity exercise session on subsequent run performance. *J. Sports Sci. Med. 10,* 665-670.

Chaabene,H., Hachana,Y., Franchini,E., Tabben,M., Mkaouer,B., Negra,Y., et al., (2015).Criterion Related Validity of Karate Specific Aerobic Test (KSAT) . *Asian J. Sports Med.* 6, e23807.

Cinarli,F. S., Kafkas,A., Eken,Ö. and Kafkas,M. E. (2016).The Effect of Somatotype Component Differences on Biomotor and Cognitive Abilities. Journal of Sport and Social Sciences. 3, 1-14.

Haugen,T., Tonnessen,E., Leirstein,S., Hem,E. and Seiler,S. (2014).Not quite so fast: effect of training at 90% sprint speed on maximal and repeated-sprint ability in soccer players. *J. Sports Sci.* 32, 1979-1986.

Invernizzi,P. L., Longo,S., Bizzi,M., Benedini,S., Merati,G. and Bosio,A. (2015).Interpretation and Perception of Two Different Kumite Fighting Intensities through an Integrated Approach Training in International Level Karatekas: an Exploratory Study. *Percept. Mot. Skills.* 121, 333-349.

Krustrup,P., Ermidis,G. and Mohr,M. (2015).Sodium bicarbonate intake improves high-intensity intermittent exercise performance in trained young men. *J. Int. Soc. Sports Nutr.* 12.

Marriott,M., Krustrup,P. and Mohr,M. (2015).Ergogenic effects of caffeine and sodium bicarbonate supplementation on intermittent exercise performance preceded by intense arm cranking exercise. *J. Int. Soc. Sports Nutr. 12.*

Martin,A. C., Heazlewood,I. T., Kitic,C. M., Lys,I. and Johnson,L. (2017).Possible Hormone Predictors Of Physical Performance In Adolescent Team Sport Athletes. *J. Strength Cond. Res.*

Nedrehagen,E. S. and Saeterbakken,A. H. (2015).The Effects of in-Season Repeated Sprint Training Compared to Regular Soccer Training. *J. Hum. Kinet.* 49, 237-244.

Nicks,C. R., Morgan,D. W., Fuller,D. K. and Caputo,J. L. (2009).The influence of respiratory muscle training upon intermittent exercise performance. *Int. J. Sports Med. 30,* 16-21.

Serpiello,F. R., McKenna,M. J., Stepto,N. K., Bishop,D. J. and Aughey,R. J. (2011).Performance and physiological responses to repeated-sprint exercise: a novel multiple-set approach. *Eur. J. Appl. Physiol. 111,* 669-678.

Sirotic,A. C. and Coutts,A. J. (2007).Physiological and performance test correlates of prolonged, high-intensity, intermittent running performance in moderately trained women team sport athletes. *J. Strength Cond. Res. 21,* 138-144.

Thompson,C., Vanhatalo,A., Jell,H., Fulford,J., Carter,J., Nyman,L., et al., (2016).Dietary nitrate supplementation improves sprint and high-intensity intermittent running performance. *Nitric Oxide. 61,* 55-61.

Zhang,Y., Nepocatych,S., Katica,C. P., Collins,A. B., Casaru,C., Balilionis,G., et al., (2014).Effect of Half Time Cooling on Thermoregulatory Responses and Soccer-Specific Performance Tests. Montenegrin Journal of Sports Science and Medicine. 3, 17-22.
