# Supplementary material for: The Yo-Yo Intermittent Tests: A Systematic Review and Structured Compendium of Test Results
Source: Front Physiol. 2018 Jul 5;9:870. doi: 10.3389/fphys.2018.00870 (PMC6041409; doi:10.3389/fphys.2018.00870)
Supplement: Supplementary file 2 [file Data_Sheet_1.PDF]

Supplementary File 1

Formula for the calculation of global means and standard deviations

Let i denote the study, $i = 1, \dots, N$ with N being the number of studies, which are supposed to be samples from an identical population.
Let x_{ij} denote the result of the test of the j-th subject in study i, $j = 1, \dots, n_i$ with n_i being the number of subjects analyzed in study i. The x_{ij} are not given. The cumulative number of subjects is $n = \sum_{i=1}^{N} n_i$.
The only information given from each study are the respective empirical local mean $\bar{x}_i = \sum_{j=1}^{n_i} x_{ij}$ and the empirical local standard deviation $s_i = \sqrt{\frac{1}{n_i-1} \sum_{j=1}^{n_i} \left(x_{ij} - \bar{x}_i\right)^2}$.

The (empirical) global mean is

$$\bar{x} = \sum_{i=1}^{N} \sum_{j=1}^{n_i} x_{ij} = \sum_{i=1}^{N} \bar{x}_i$$

and the (empirical) global variance is

$$s^2 = \frac{1}{n-1} \sum_{i=1}^{g} \sum_{j=1}^{n_i} \left(x_{ij} - \bar{x}\right)^2 = \frac{1}{n-1} \sum_{i=1}^{g} \sum_{j=1}^{n_i} \left(x_{ij} - \bar{x}_i + \bar{x}_i - \bar{x}\right)^2$$

$$= \frac{1}{n-1} \left(\sum_i \sum_j \left(x_{ij} - \bar{x}_i\right)^2 + \sum_i \sum_j 2\left(x_{ij} - \bar{x}_i\right)(\bar{x}_i - \bar{x}) + \sum_i \sum_j (\bar{x}_i - \bar{x})^2 \right)$$

$$= \frac{1}{n-1} \left(\sum_i (n_i - 1) s_i^2 + 2 \sum_i (\bar{x}_i - \bar{x}) \sum_j \left(x_{ij} - \bar{x}_i\right) + \sum_i n_i (\bar{x}_i - \bar{x})^2 \right)$$

$$= \frac{1}{n-1} \left(\sum_i (n_i - 1) s_i^2 + \sum_i n_i (\bar{x}_i - \bar{x})^2 \right)$$

resulting in the (empirical) global standard deviation

$$s = \sqrt{\frac{1}{n-1} \left(\sum_{i=1}^{N} (n_i - 1) s_i^2 + \sum_{i=1}^{N} n_i (\bar{x}_i - \bar{x})^2 \right)}$$
