# Supplementary figures and images for: The Yo-Yo Intermittent Tests: A Systematic Review and Structured Compendium of Test Results
Source: Front Physiol. 2018 Jul 5;9:870. doi: 10.3389/fphys.2018.00870 (PMC6041409; doi:10.3389/fphys.2018.00870)

## Forestplot: YYIE1 Handball Male Sub-Elite

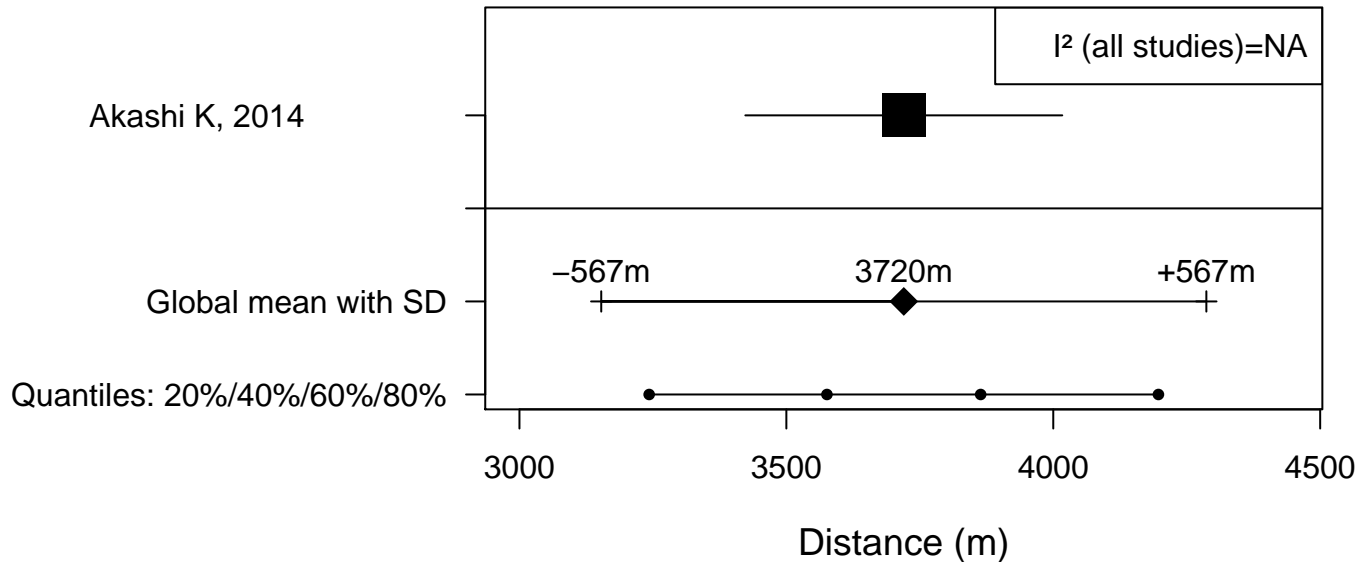

Supplement: Supplementary file 3 [file Data_Sheet_2.ZIP › Forestplots/Forestplot_YYIE1_Handball_Male_Sub-Elite.pdf]

## Forestplot: YYIE1 Inactive Female

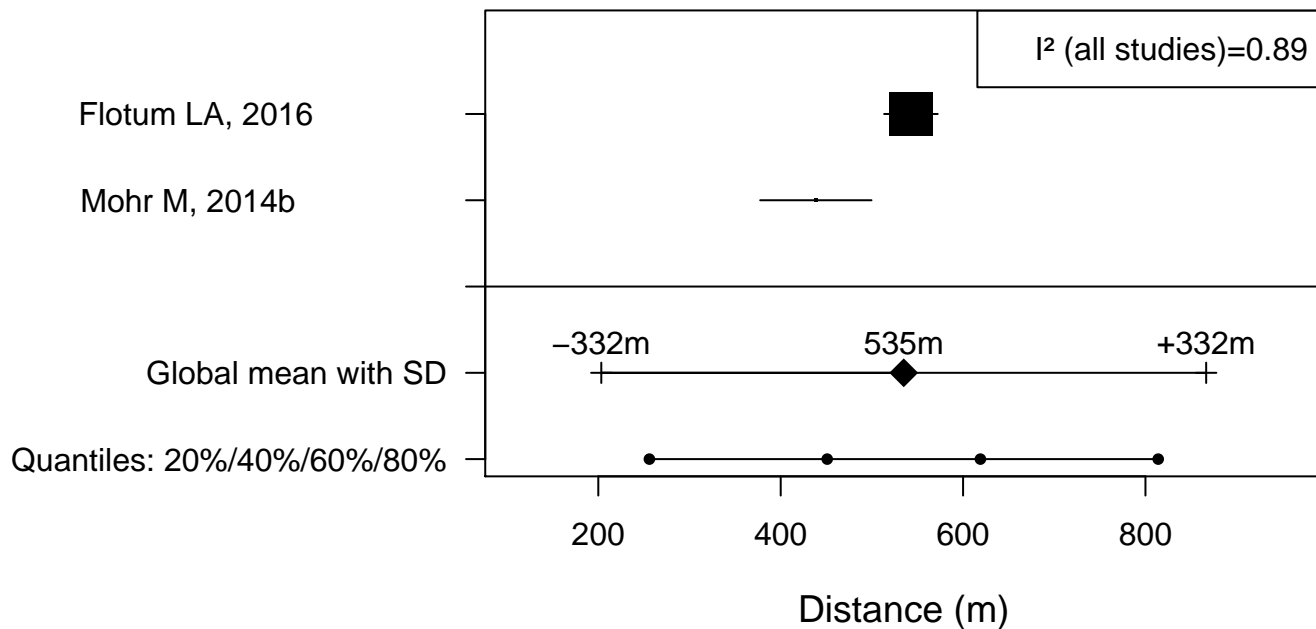

Supplement: Supplementary file 3 [file Data_Sheet_2.ZIP › Forestplots/Forestplot_YYIE1_Inactive_Female_.pdf]

## Forestplot: YYIE1 Recreational Female

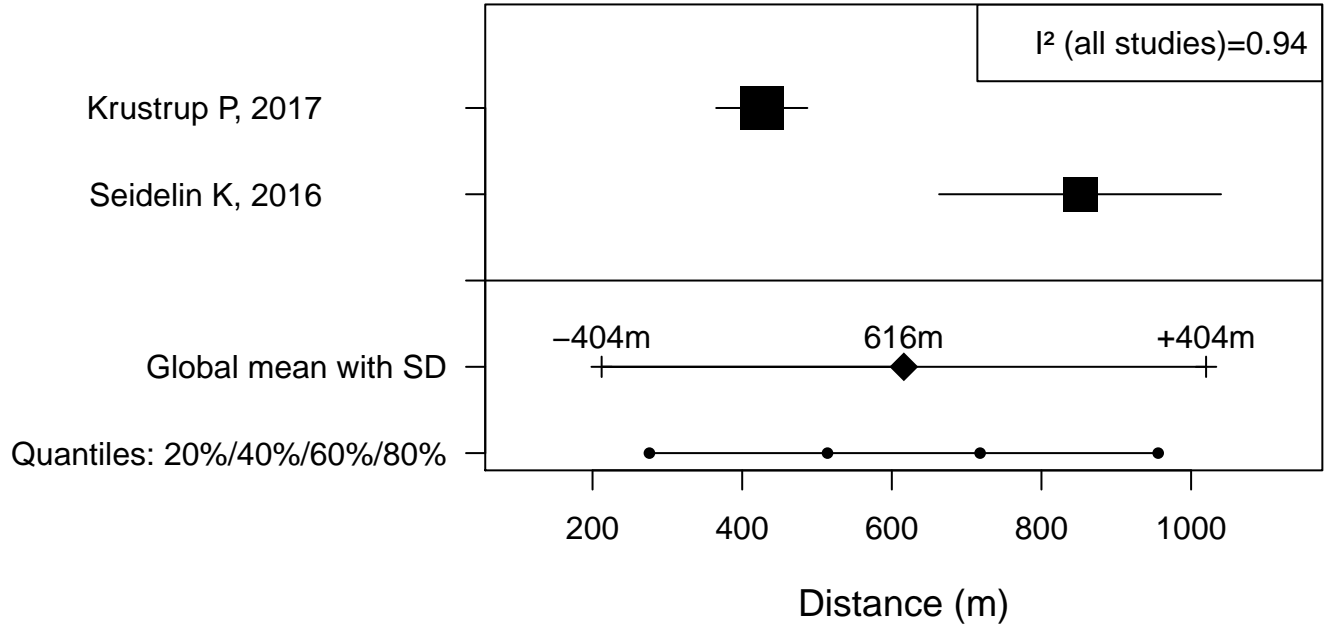

Supplement: Supplementary file 3 [file Data_Sheet_2.ZIP › Forestplots/Forestplot_YYIE1_Recreational_Female_.pdf]

## Forestplot: YYIE1 Referees Male Elite

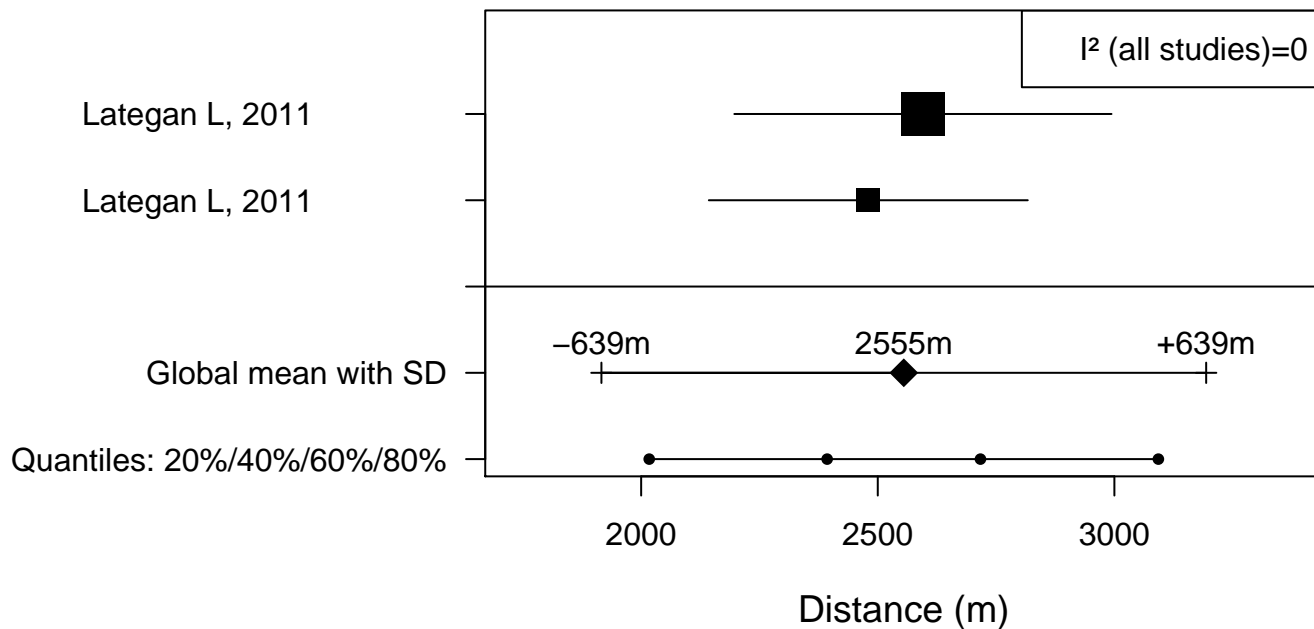

Supplement: Supplementary file 3 [file Data_Sheet_2.ZIP › Forestplots/Forestplot_YYIE1_Referees_Male_Elite.pdf]

## Forestplot: YYIE1 Soccer Female Sub-Elite

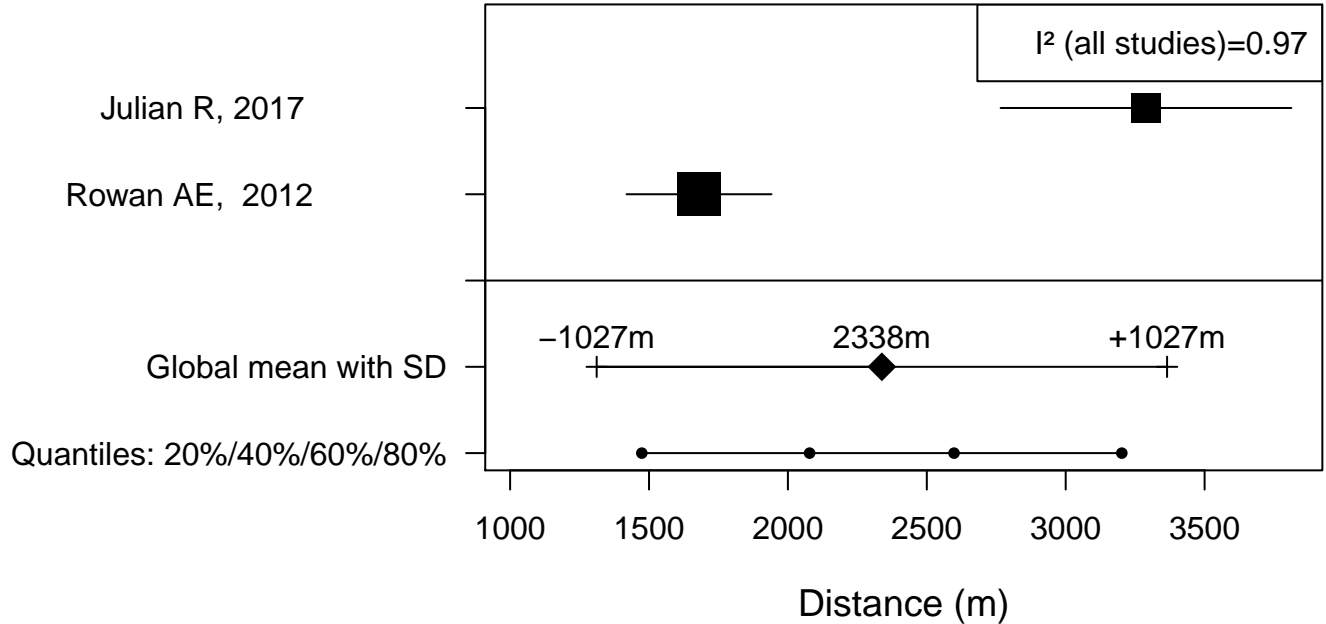

Supplement: Supplementary file 3 [file Data_Sheet_2.ZIP › Forestplots/Forestplot_YYIE1_Soccer_Female_Sub-Elite.pdf]

## Forestplot: YYIE1 Soccer Male Amateur

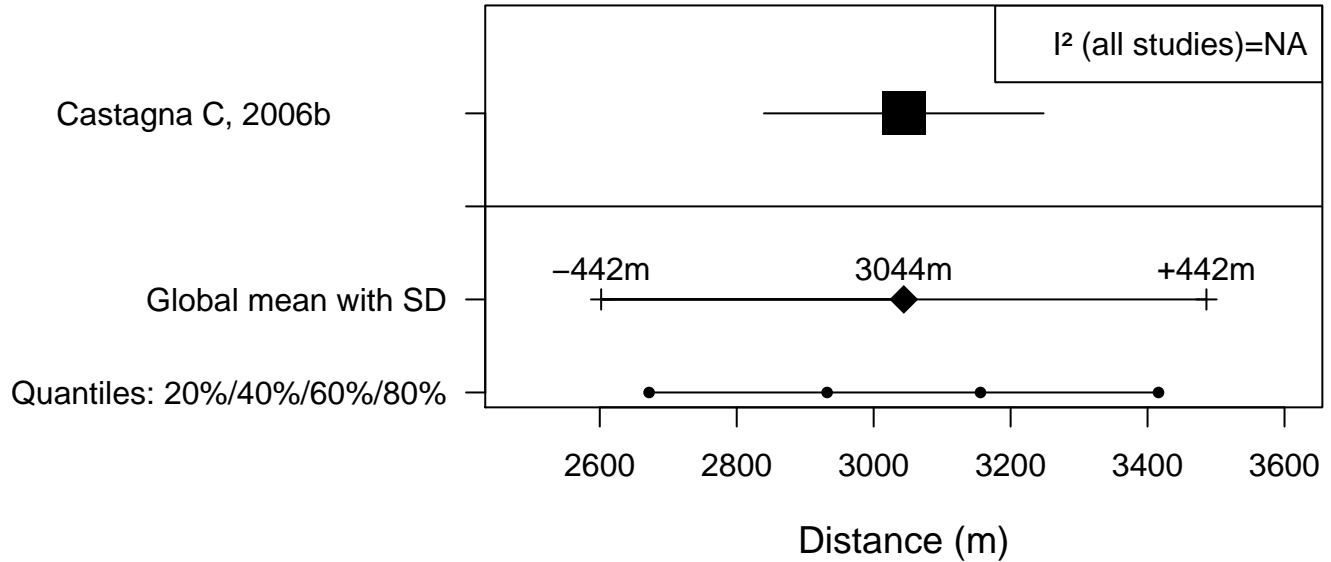

Supplement: Supplementary file 3 [file Data_Sheet_2.ZIP › Forestplots/Forestplot_YYIE1_Soccer_Male_Amateur.pdf]

## Forestplot: YYIE1 Soccer Male Elite

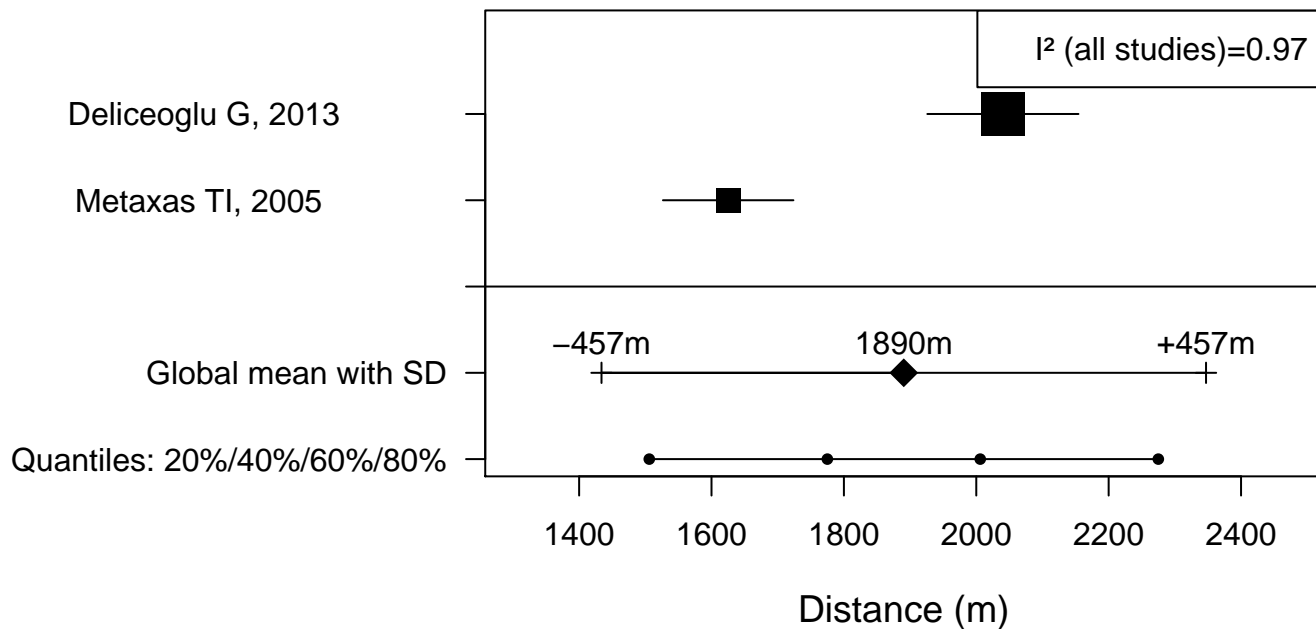

Supplement: Supplementary file 3 [file Data_Sheet_2.ZIP › Forestplots/Forestplot_YYIE1_Soccer_Male_Elite.pdf]

## Forestplot: YYIE2 Handball Male Elite

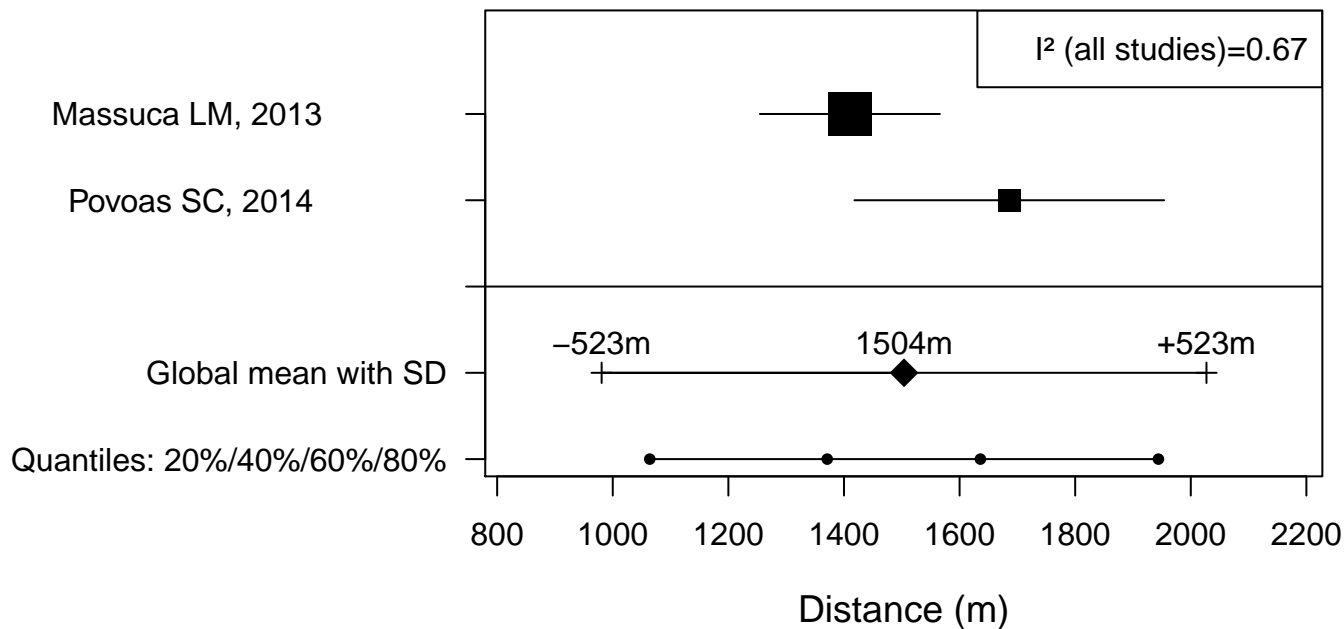

Supplement: Supplementary file 3 [file Data_Sheet_2.ZIP › Forestplots/Forestplot_YYIE2_Handball_Male_Elite.pdf]

## Forestplot: YYIE2 Handball Male Sub-Elite

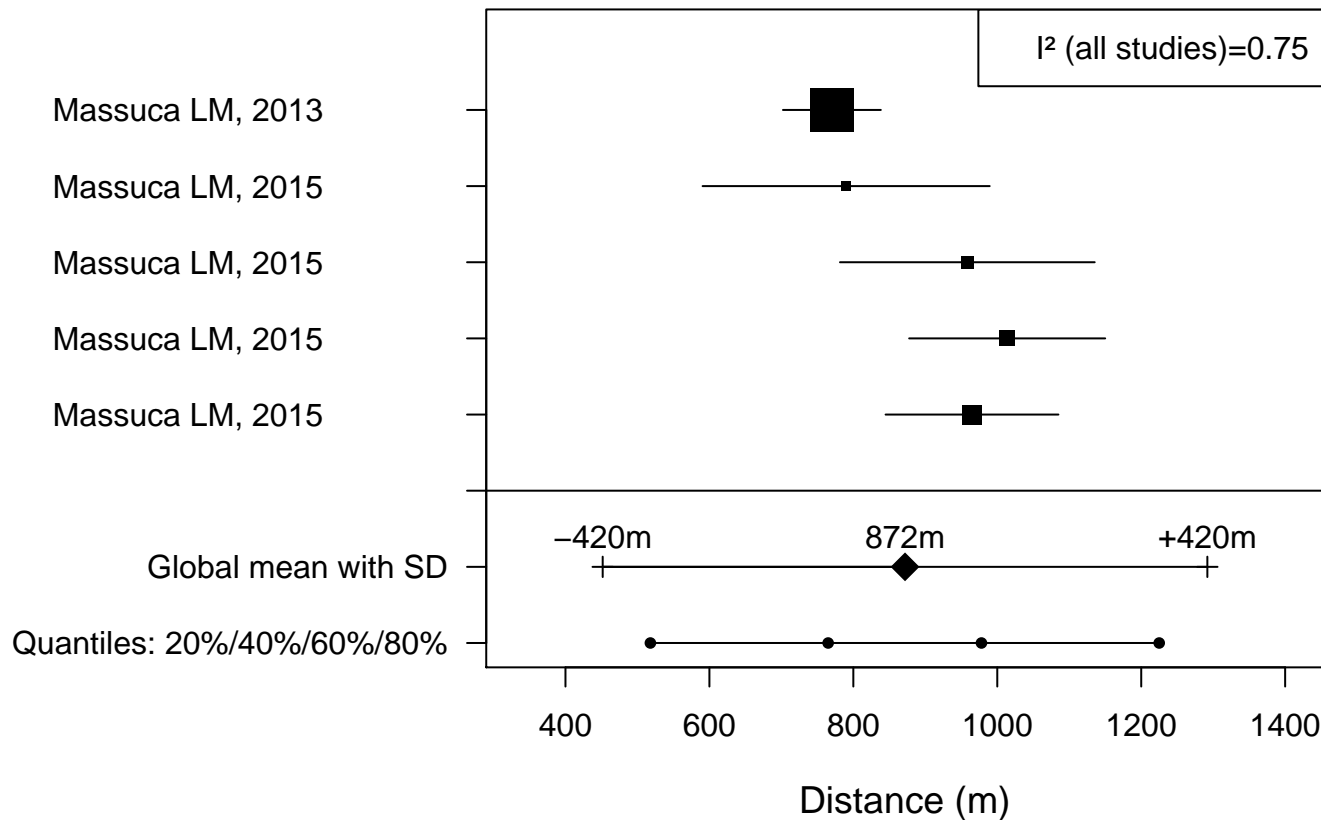

Supplement: Supplementary file 3 [file Data_Sheet_2.ZIP › Forestplots/Forestplot_YYIE2_Handball_Male_Sub-Elite.pdf]

## Forestplot: YYIE2 Inactive Female

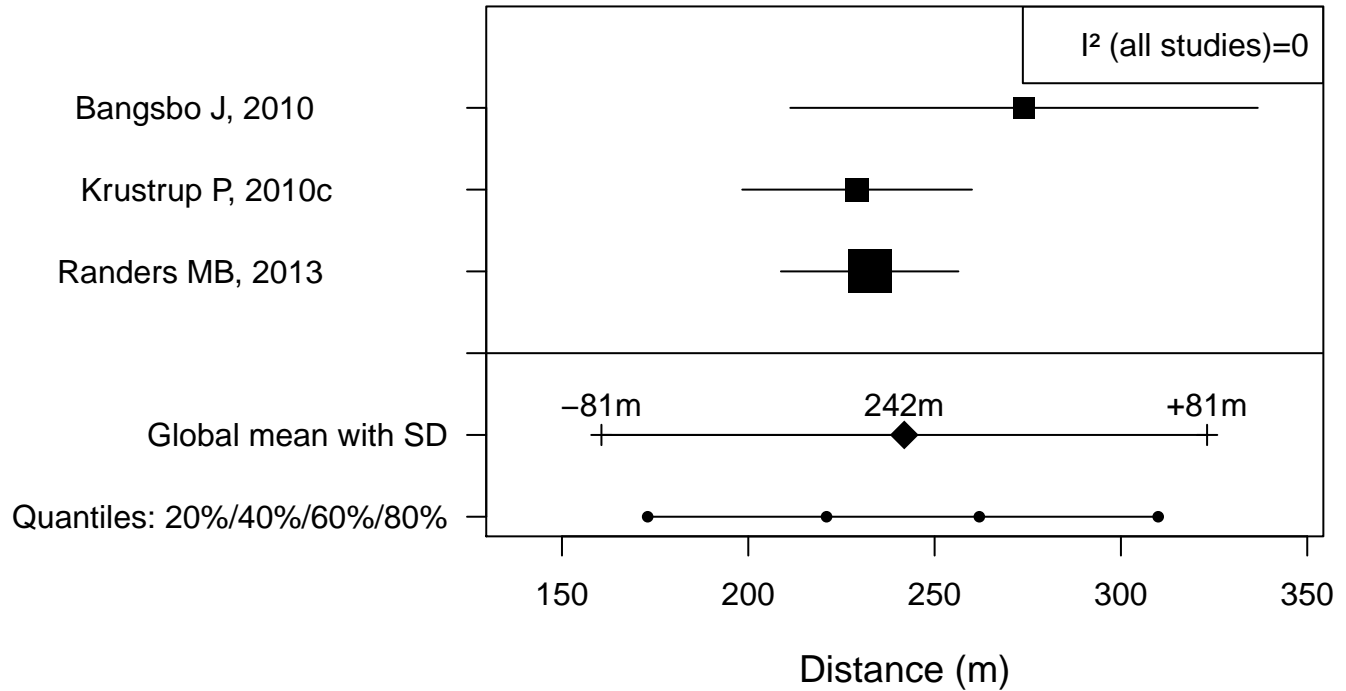

Supplement: Supplementary file 3 [file Data_Sheet_2.ZIP › Forestplots/Forestplot_YYIE2_Inactive_Female_.pdf]

## Forestplot: YYIE2 Inactive Male

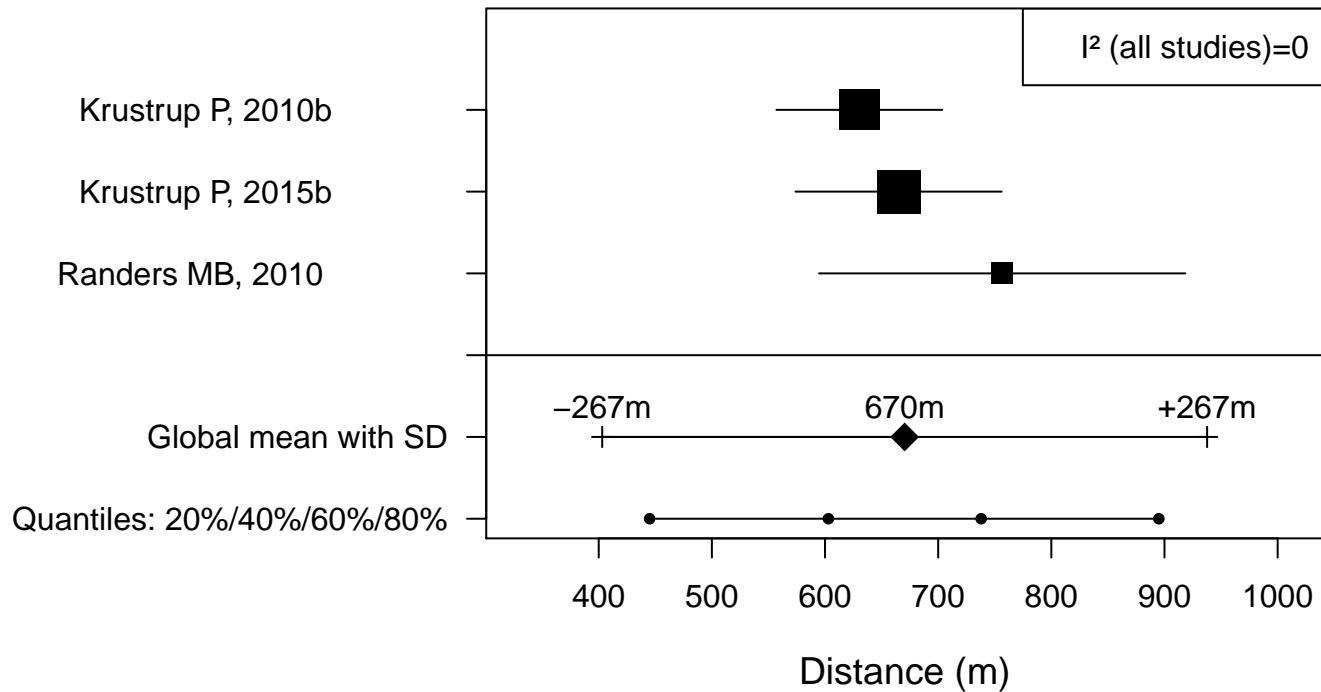

Supplement: Supplementary file 3 [file Data_Sheet_2.ZIP › Forestplots/Forestplot_YYIE2_Inactive_Male_.pdf]

## Forestplot: YYIE2 Recreational Male

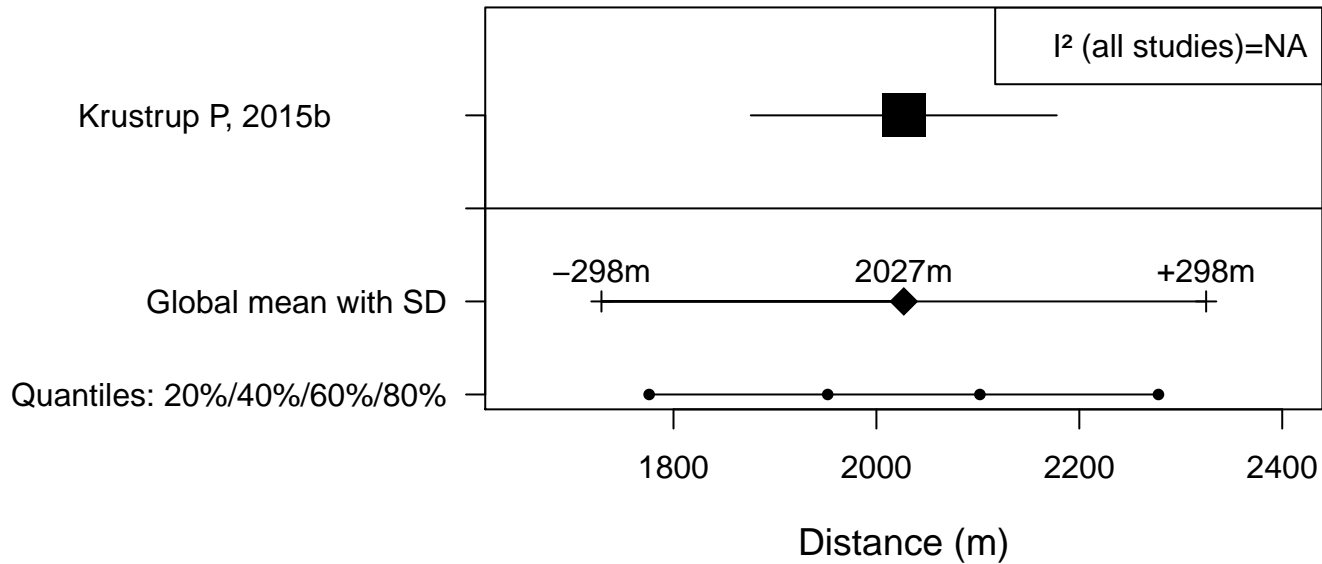

Supplement: Supplementary file 3 [file Data_Sheet_2.ZIP › Forestplots/Forestplot_YYIE2_Recreational_Male_.pdf]

## Forestplot: YYIE2 Referees Male Elite

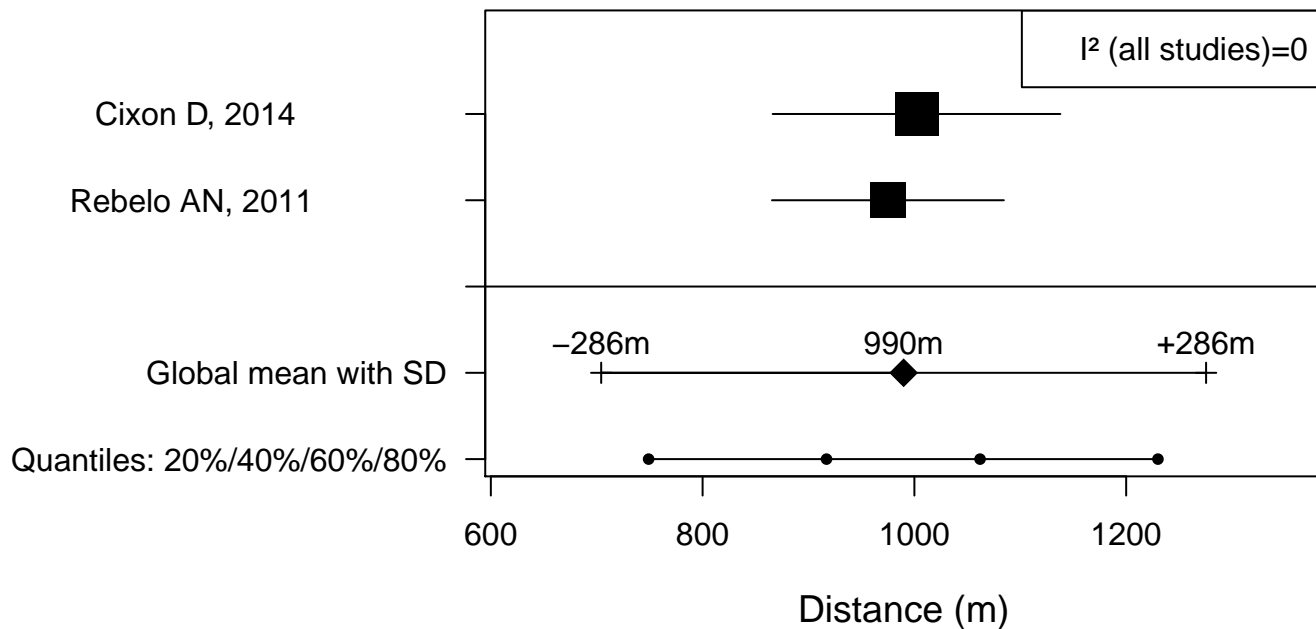

Supplement: Supplementary file 3 [file Data_Sheet_2.ZIP › Forestplots/Forestplot_YYIE2_Referees_Male_Elite.pdf]

## Forestplot: YYIE2 Soccer Female Amateur

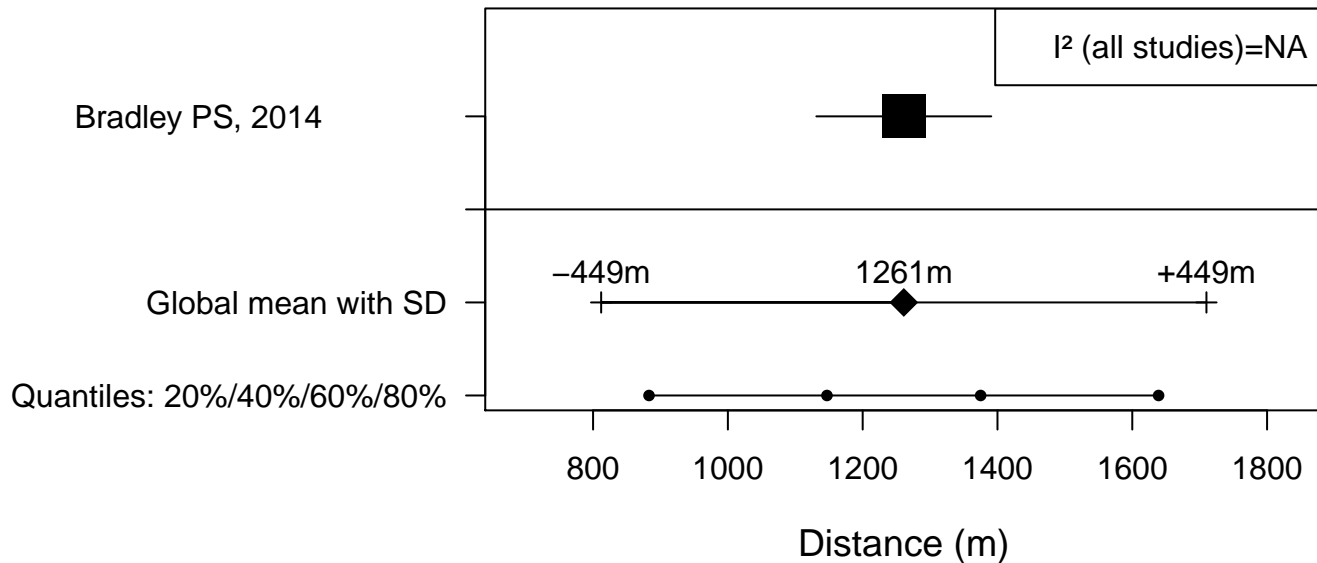

Supplement: Supplementary file 3 [file Data_Sheet_2.ZIP › Forestplots/Forestplot_YYIE2_Soccer_Female_Amateur.pdf]

## Forestplot: YYIE2 Soccer Female Elite

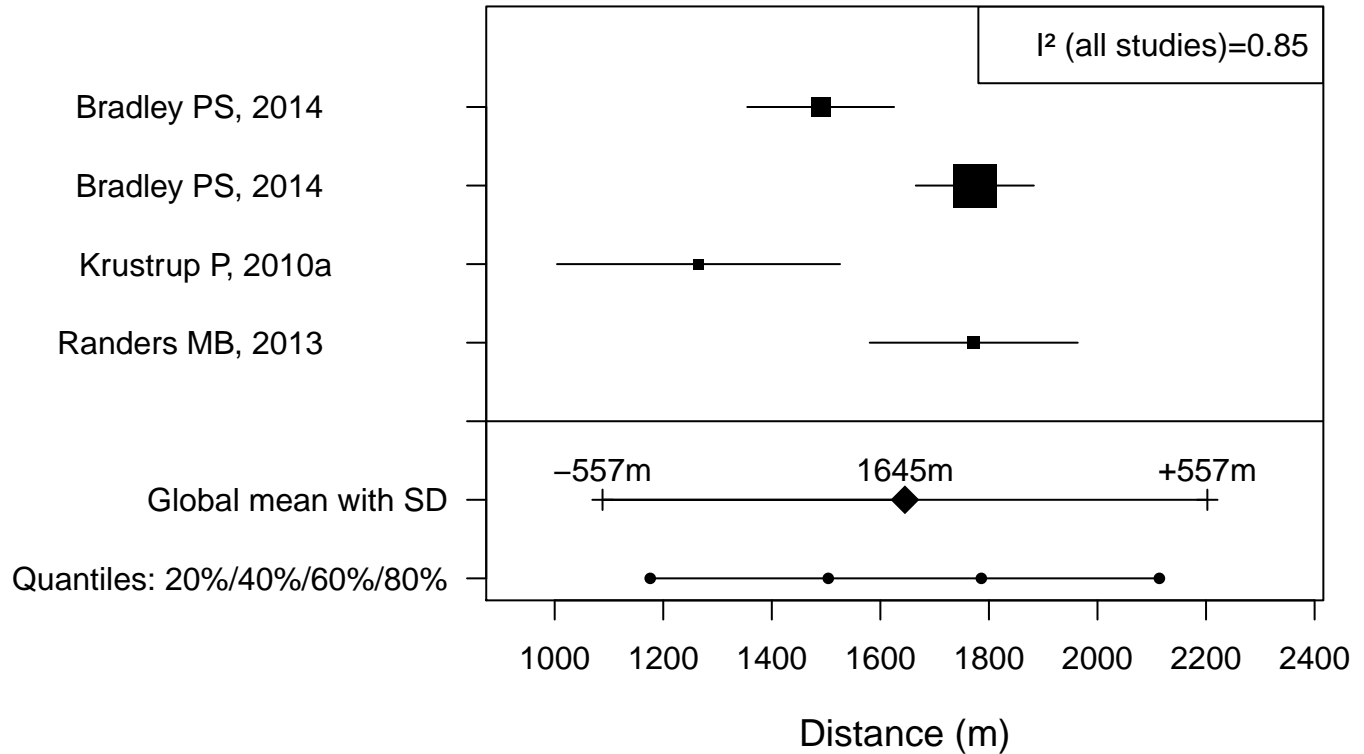

Supplement: Supplementary file 3 [file Data_Sheet_2.ZIP › Forestplots/Forestplot_YYIE2_Soccer_Female_Elite.pdf]

## Forestplot: YYIE2 Soccer Female Sub-Elite

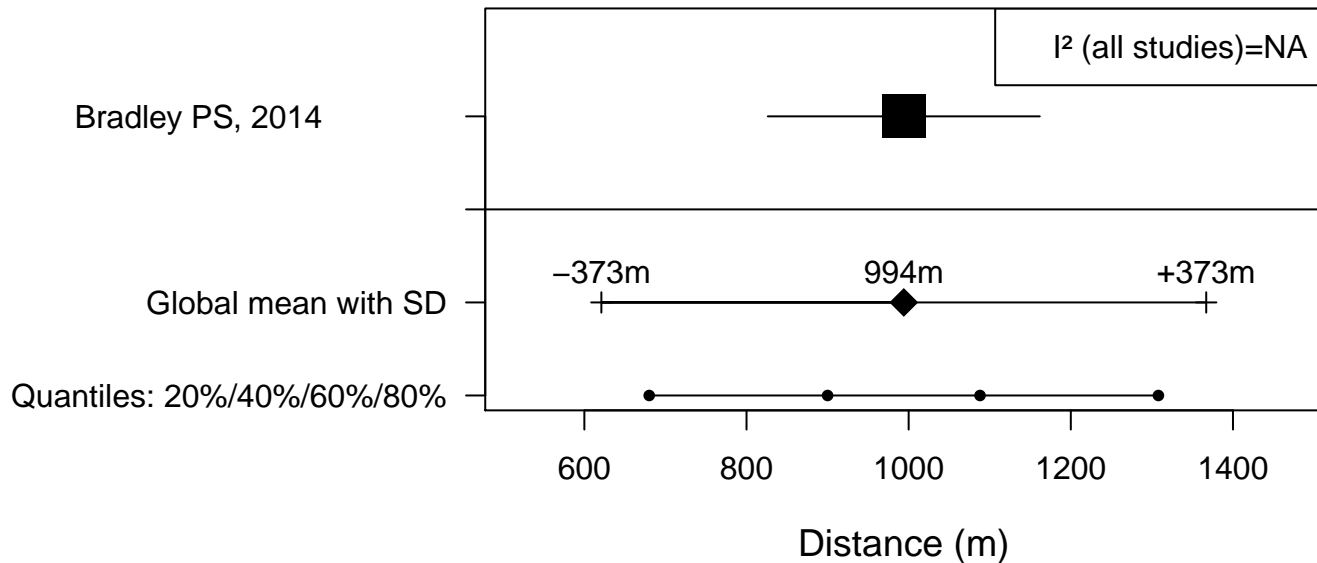

Supplement: Supplementary file 3 [file Data_Sheet_2.ZIP › Forestplots/Forestplot_YYIE2_Soccer_Female_Sub-Elite.pdf]

# Forestplot: YYIE2 Soccer Male Amateur

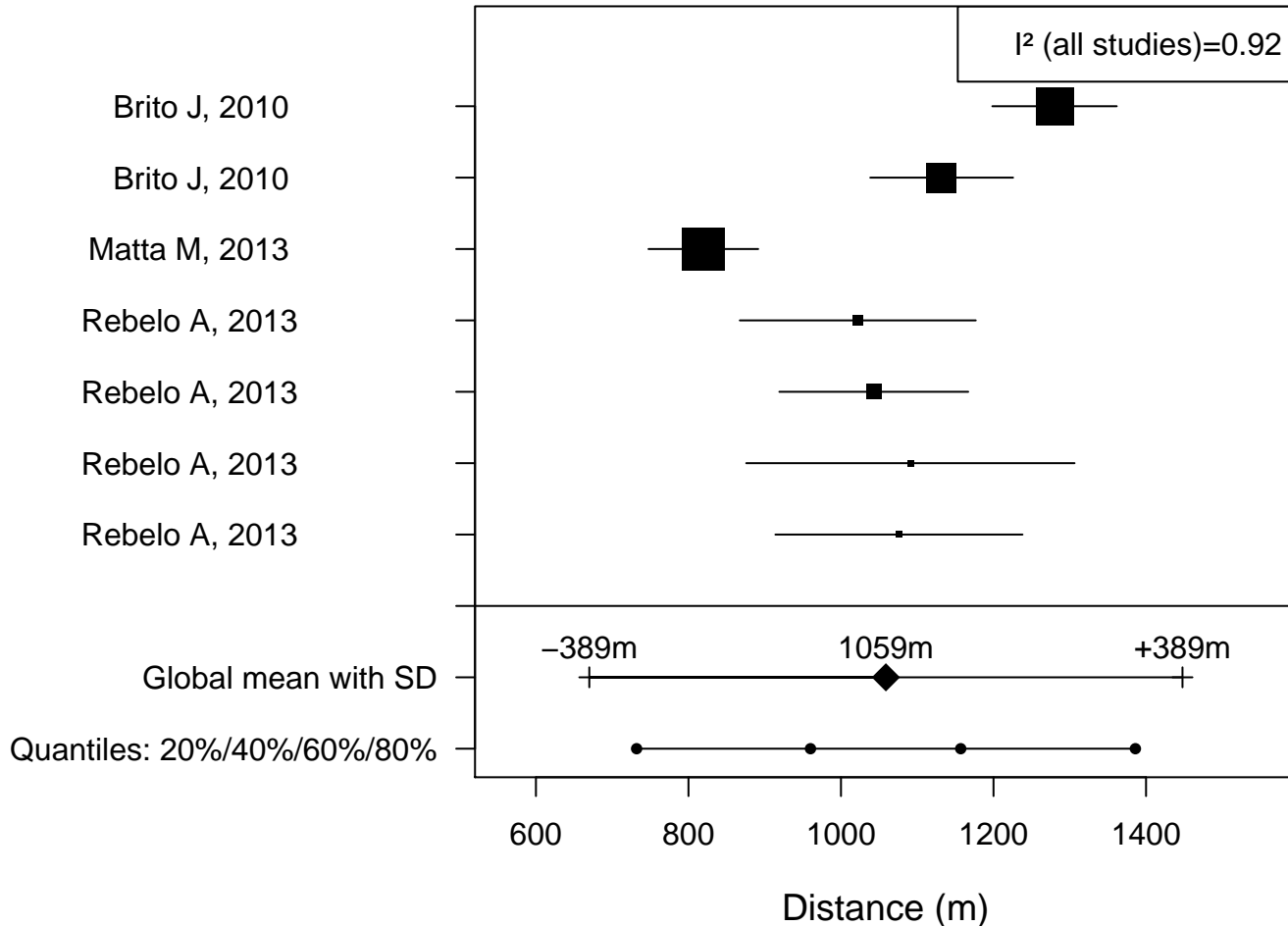

Supplement: Supplementary file 3 [file Data_Sheet_2.ZIP › Forestplots/Forestplot_YYIE2_Soccer_Male_Amateur.pdf]

# Forestplot: YYIE2 Soccer Male Elite

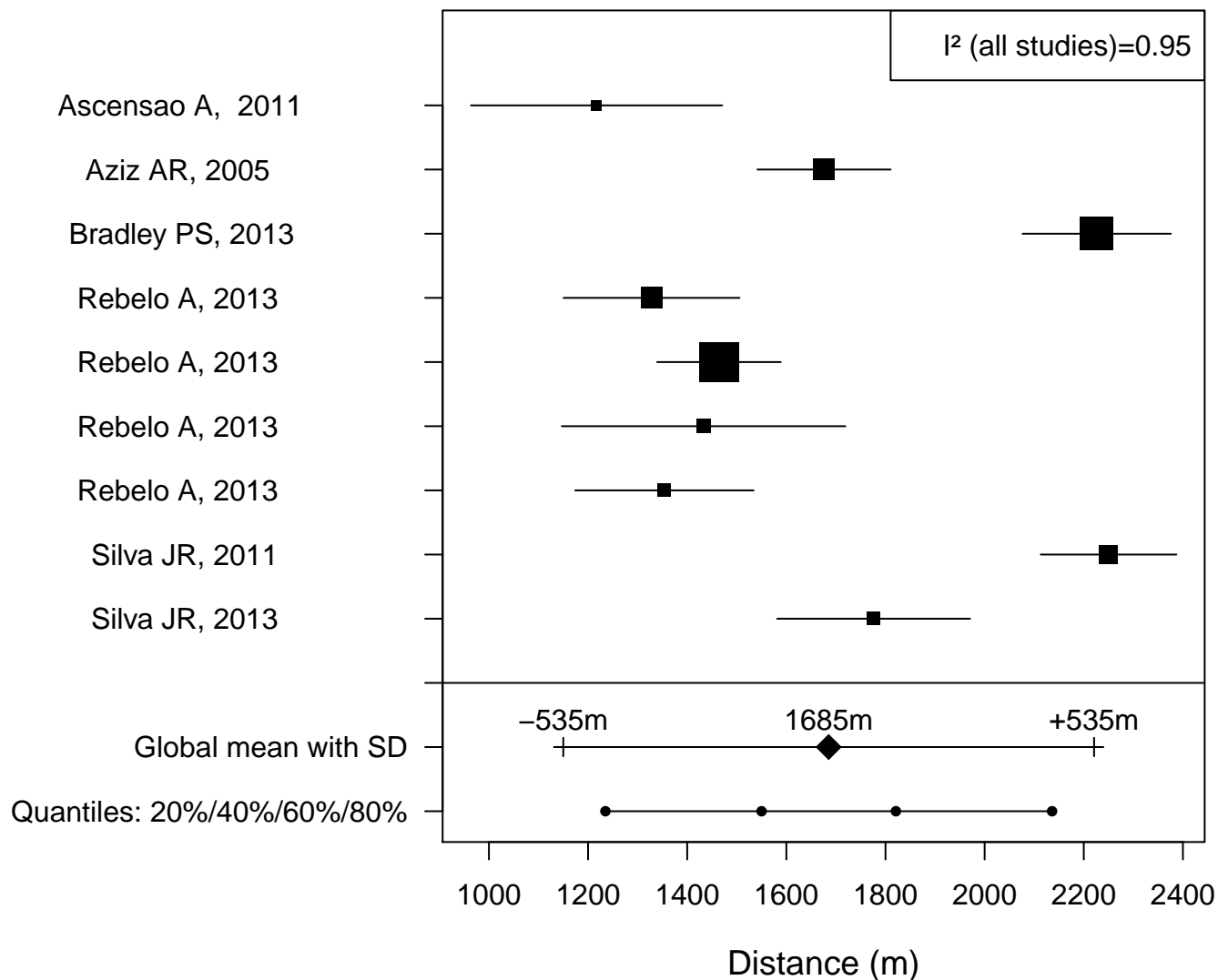

Supplement: Supplementary file 3 [file Data_Sheet_2.ZIP › Forestplots/Forestplot_YYIE2_Soccer_Male_Elite.pdf]

## Forestplot: YYIE2 Soccer Male Sub-Elite

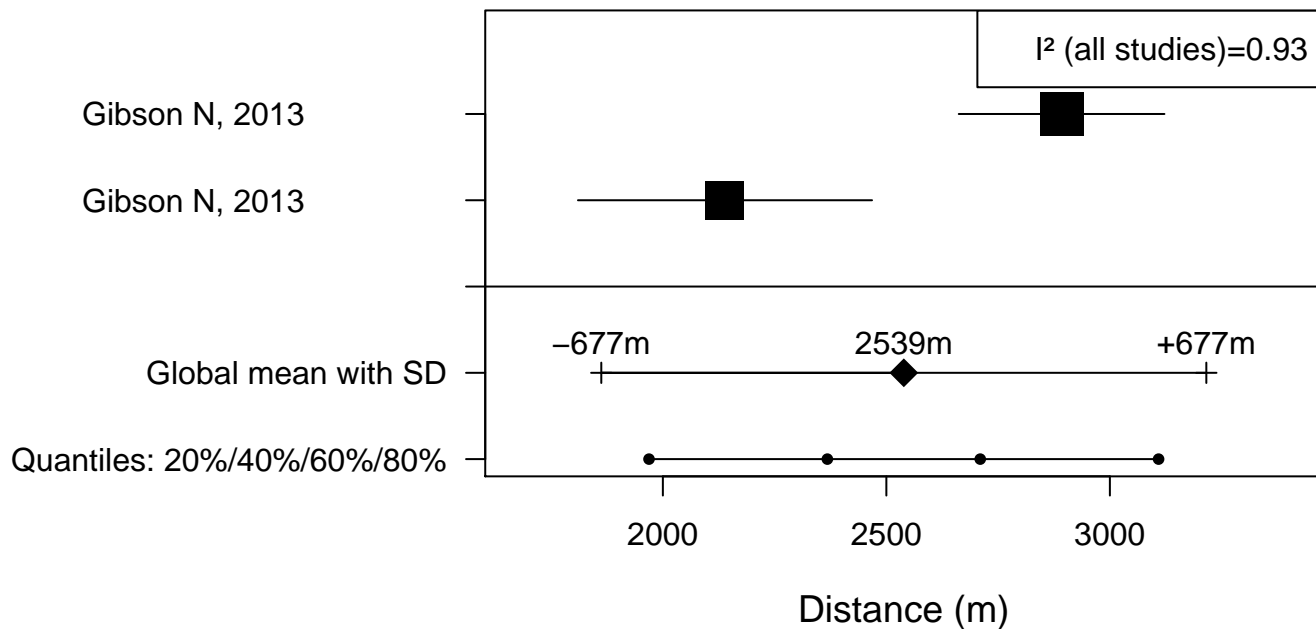

Supplement: Supplementary file 3 [file Data_Sheet_2.ZIP › Forestplots/Forestplot_YYIE2_Soccer_Male_Sub-Elite.pdf]

# Forestplot: YYIE2 Soccer Male Top-Elite

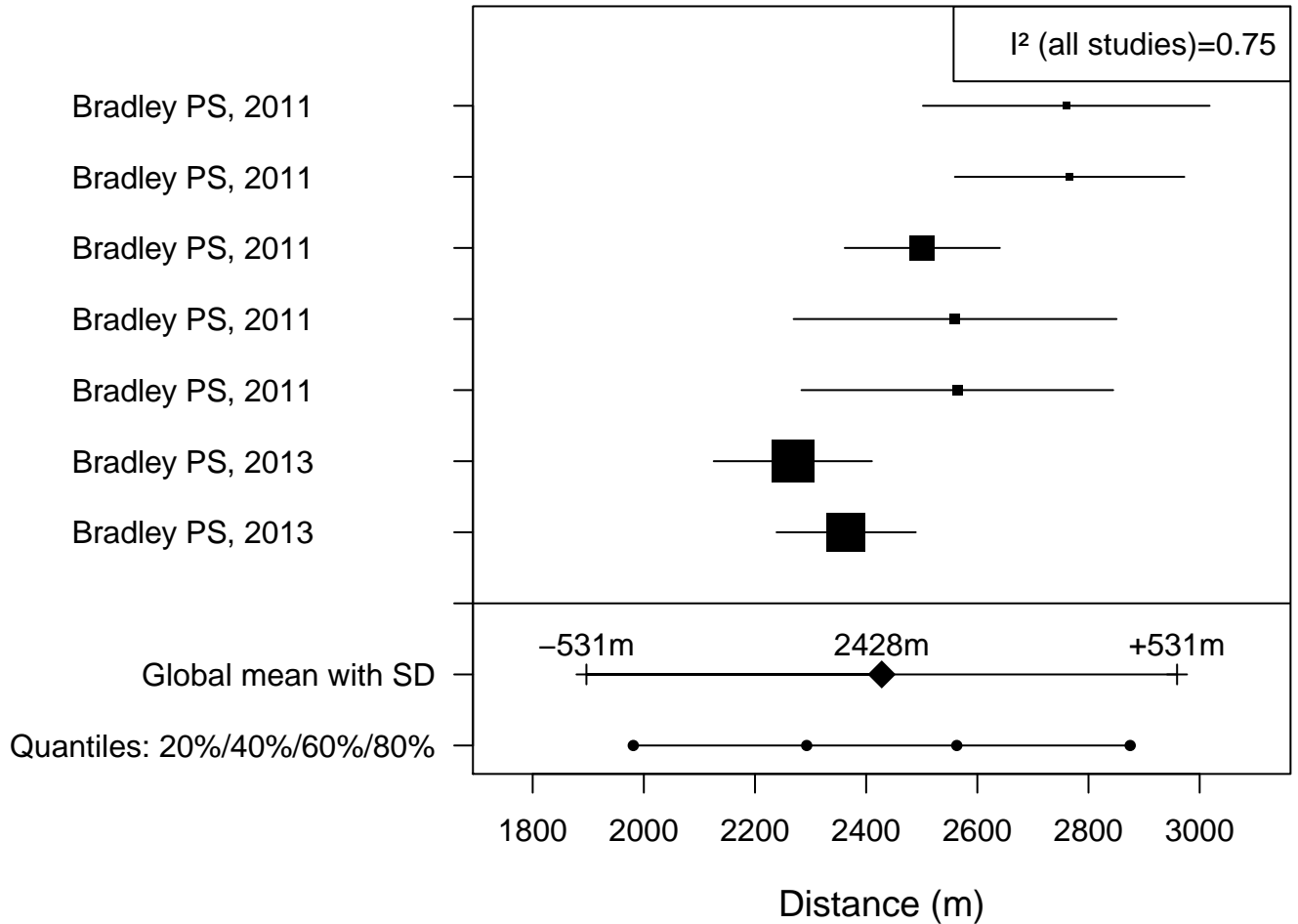

Supplement: Supplementary file 3 [file Data_Sheet_2.ZIP › Forestplots/Forestplot_YYIE2_Soccer_Male_Top-Elite.pdf]

## Forestplot: YYIR1 Basketball Female Sub-Elite or Elite

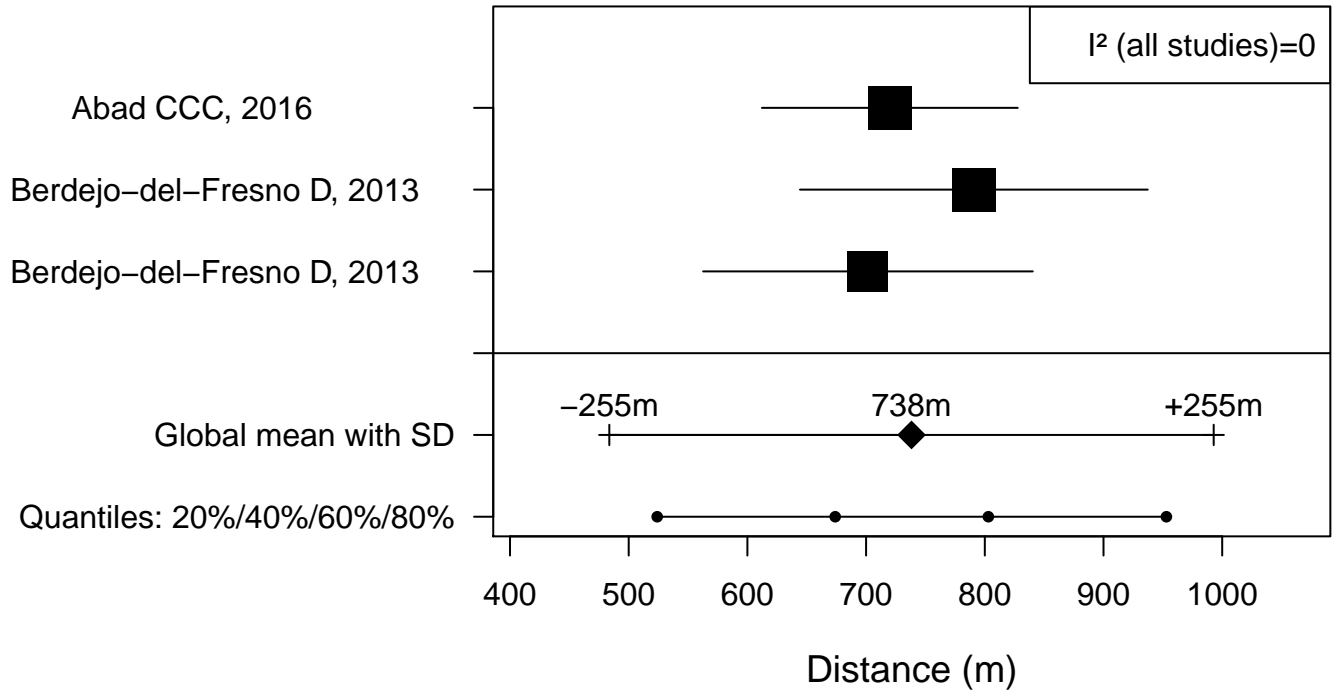

Supplement: Supplementary file 3 [file Data_Sheet_2.ZIP › Forestplots/Forestplot_YYIR1_Basketball_Female_Sub-Elite or Elite.pdf]

## Forestplot: YYIR1 Basketball Male Amateur

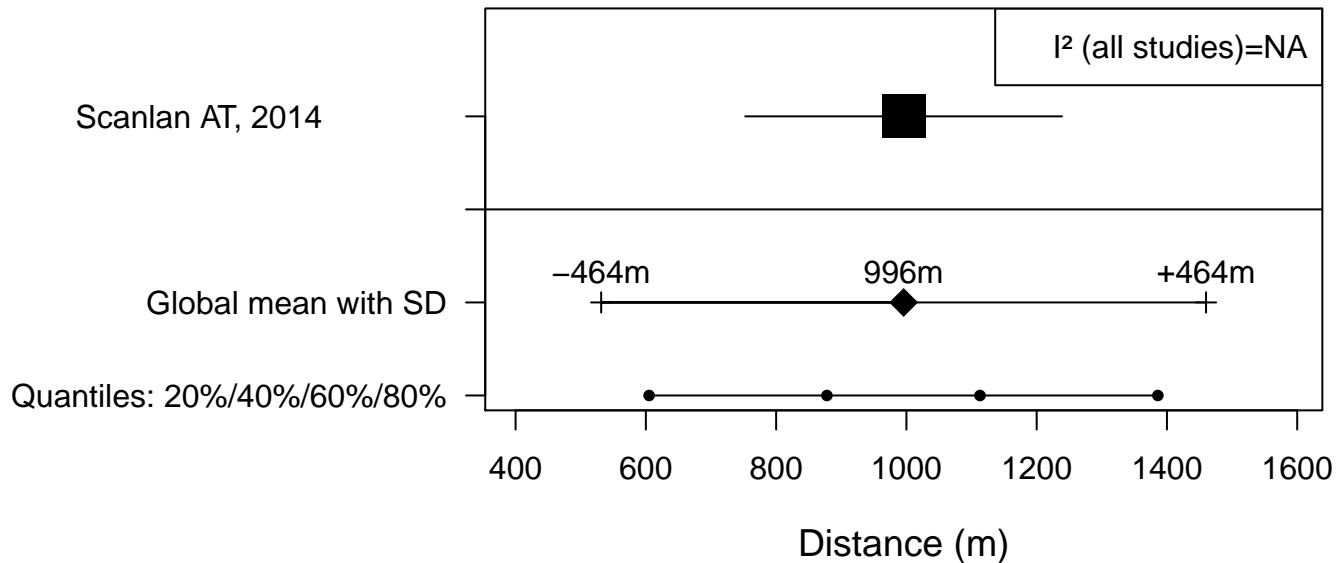

Supplement: Supplementary file 3 [file Data_Sheet_2.ZIP › Forestplots/Forestplot_YYIR1_Basketball_Male_Amateur.pdf]

# Forestplot: YYIR1 Basketball Male Elite

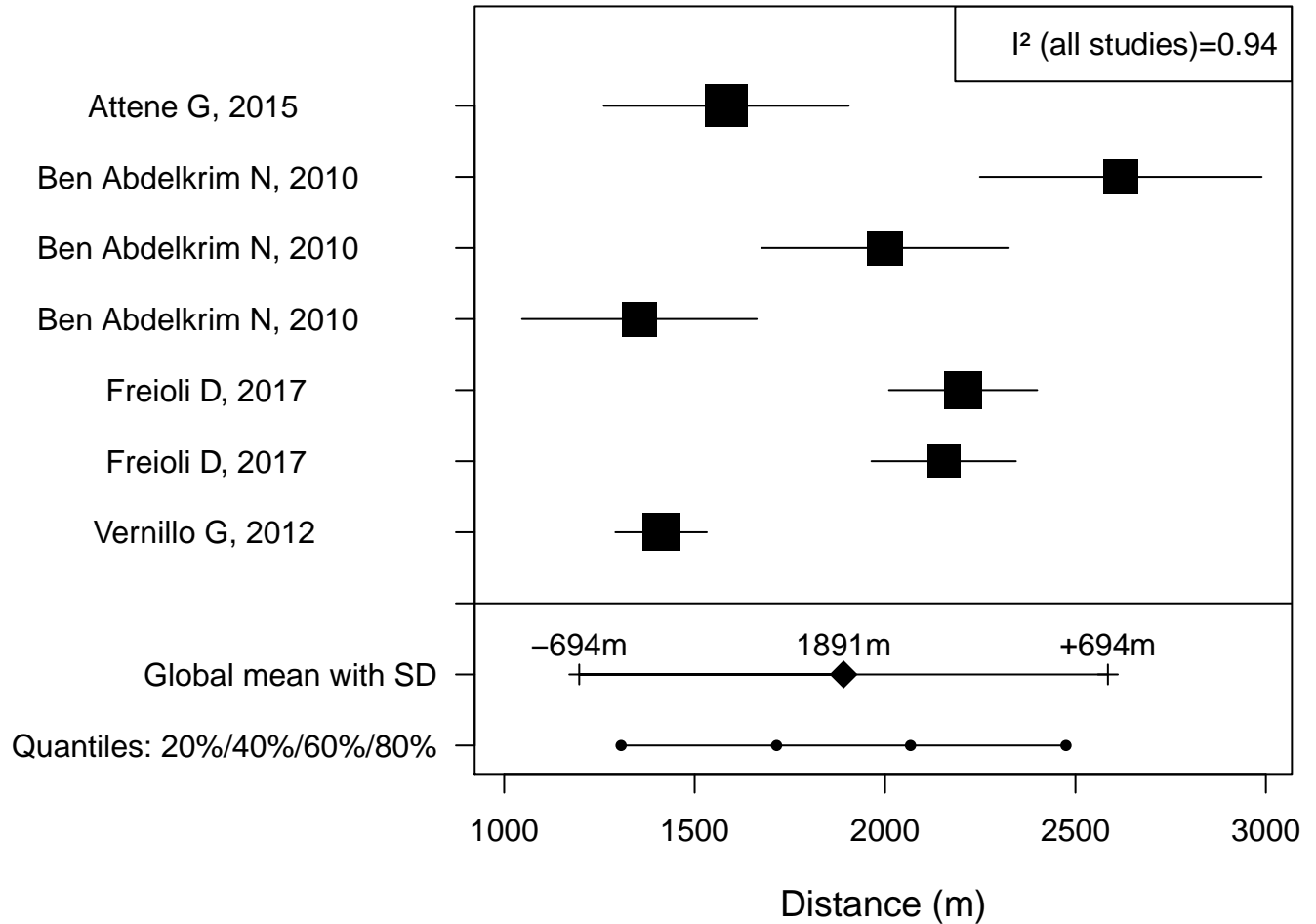

Supplement: Supplementary file 3 [file Data_Sheet_2.ZIP › Forestplots/Forestplot_YYIR1_Basketball_Male_Elite.pdf]

## Forestplot: YYIR1 Basketball Male Sub-Elite

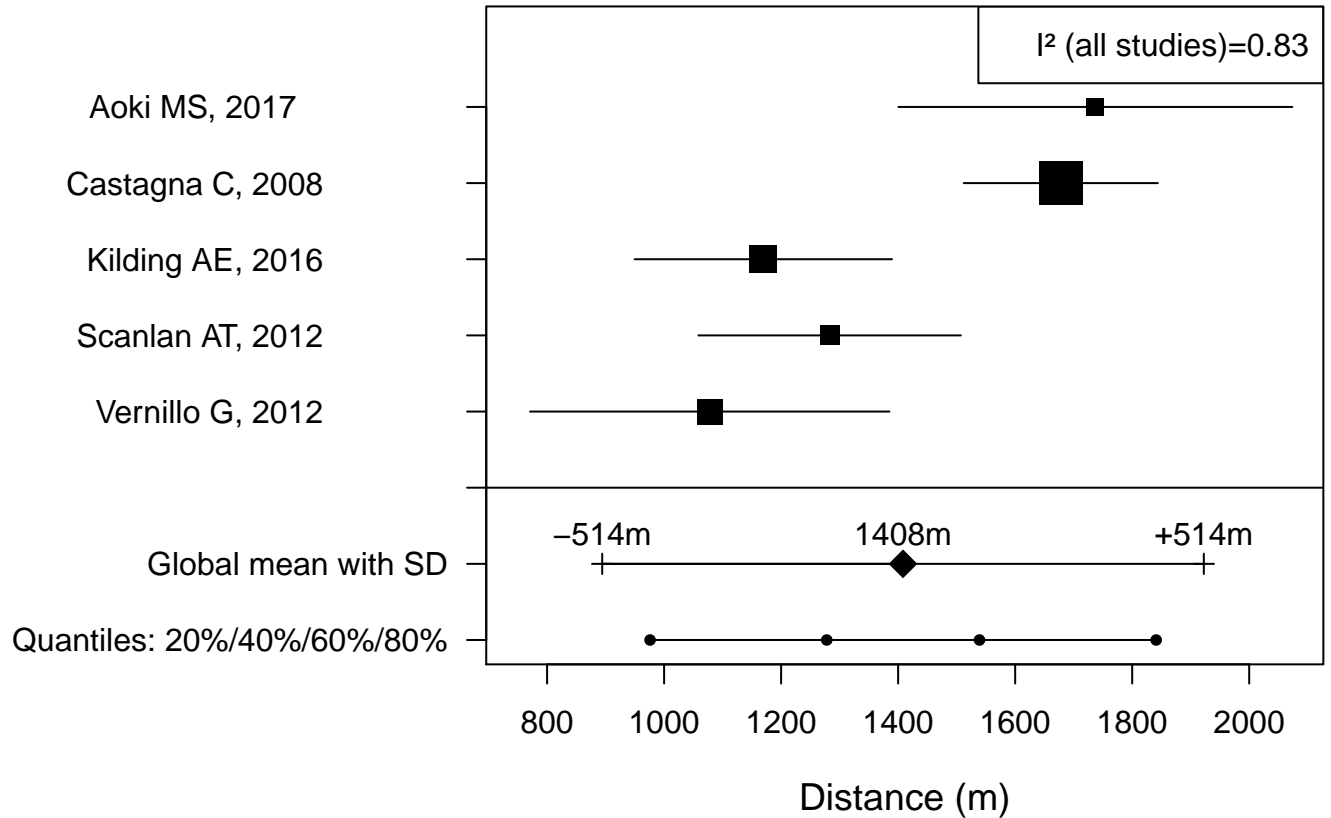

Supplement: Supplementary file 3 [file Data_Sheet_2.ZIP › Forestplots/Forestplot_YYIR1_Basketball_Male_Sub-Elite.pdf]

## Forestplot: YYIR1 Cricket Male Elite

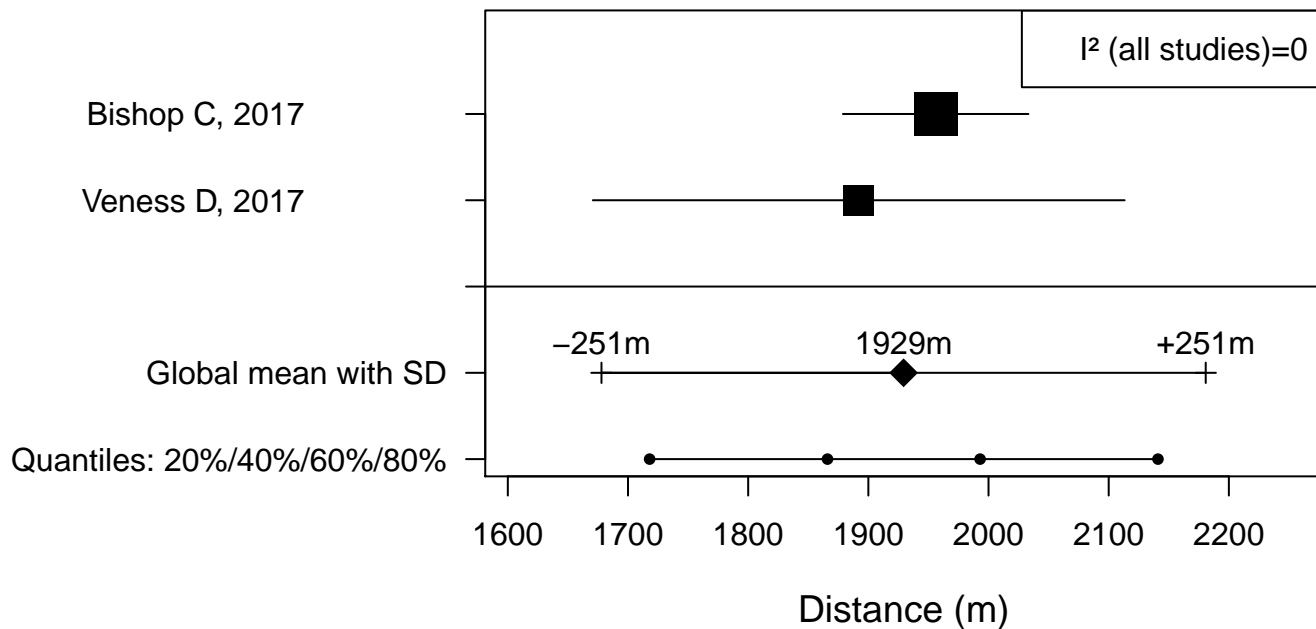

Supplement: Supplementary file 3 [file Data_Sheet_2.ZIP › Forestplots/Forestplot_YYIR1_Cricket_Male_Elite.pdf]

## Forestplot: YYIR1 Cricket Male Sub-Elite

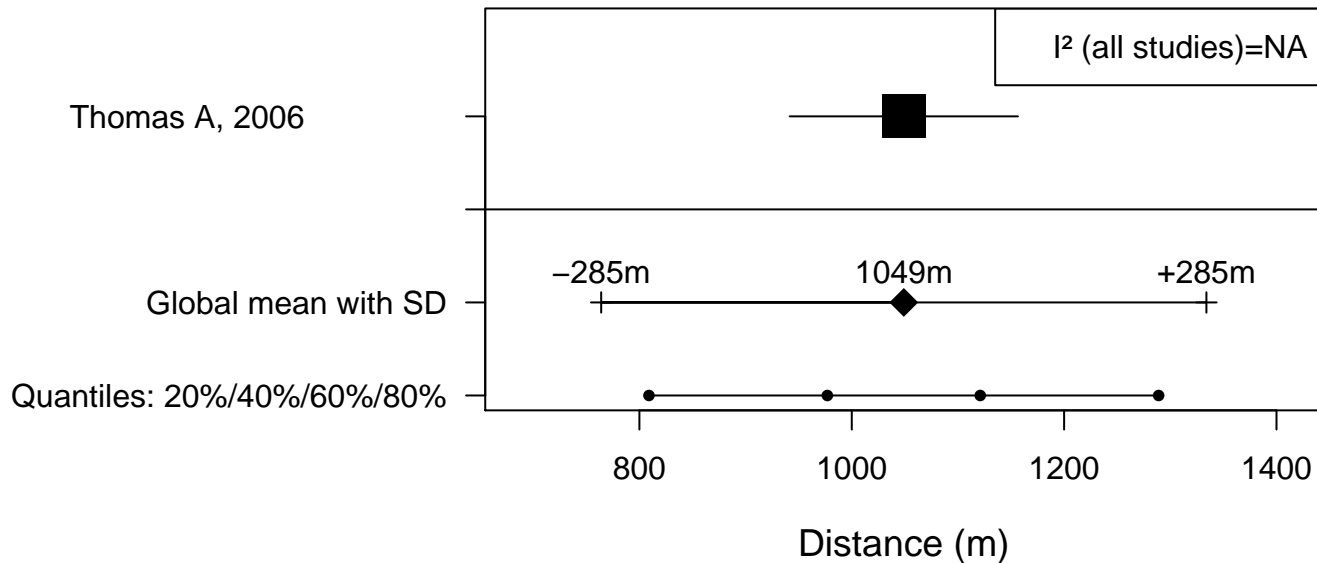

Supplement: Supplementary file 3 [file Data_Sheet_2.ZIP › Forestplots/Forestplot_YYIR1_Cricket_Male_Sub-Elite.pdf]

## Forestplot: YYIR1 Football Male Sub-Elite or Elite

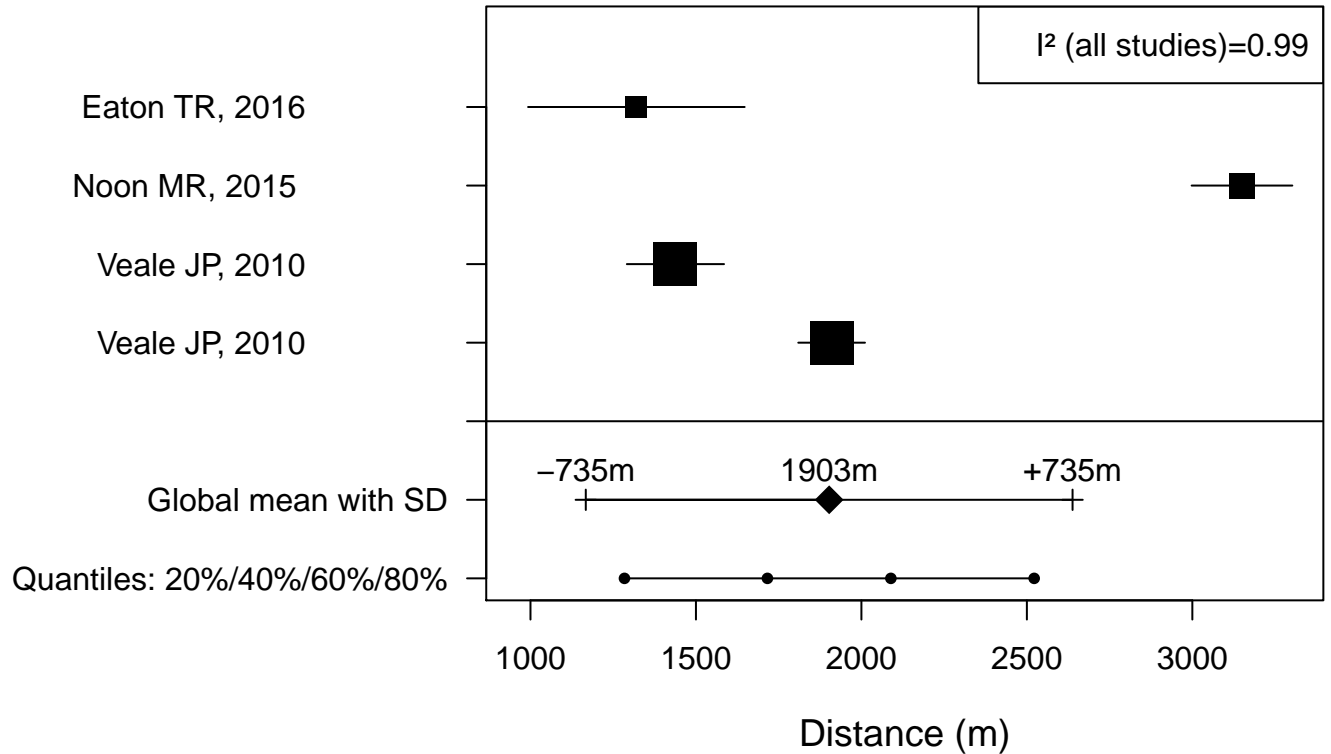

Supplement: Supplementary file 3 [file Data_Sheet_2.ZIP › Forestplots/Forestplot_YYIR1_Football_Male_Sub-Elite or Elite.pdf]

## Forestplot: YYIR1 Futsal Female Amateur

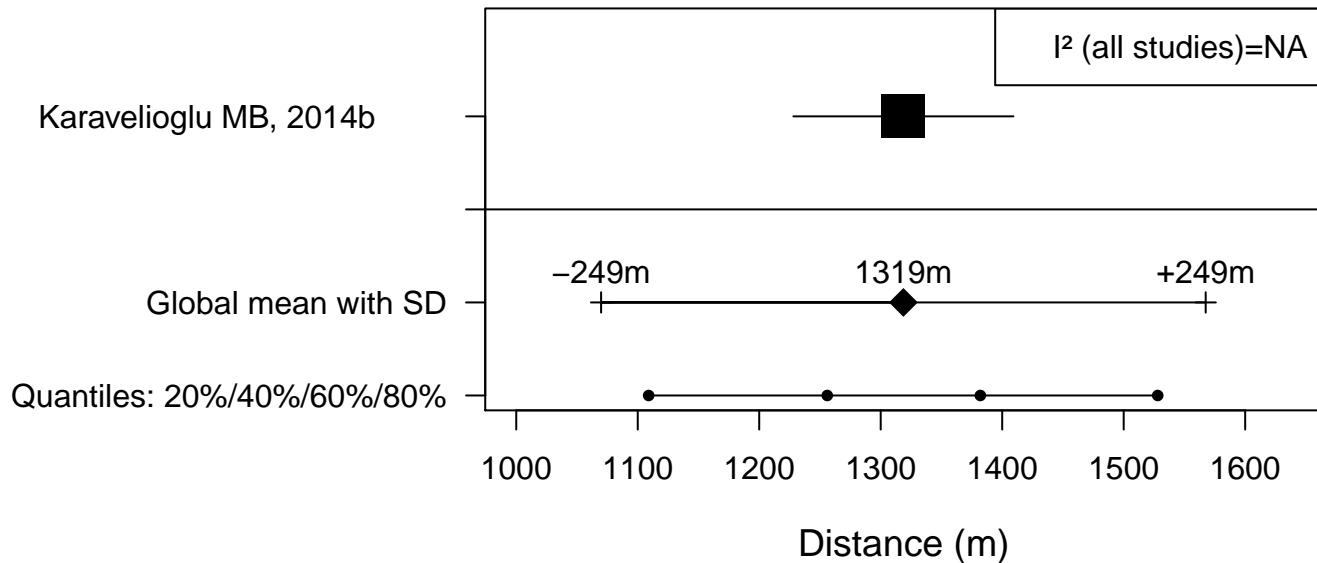

Supplement: Supplementary file 3 [file Data_Sheet_2.ZIP › Forestplots/Forestplot_YYIR1_Futsal_Female_Amateur.pdf]

## Forestplot: YYIR1 Futsal Male Elite

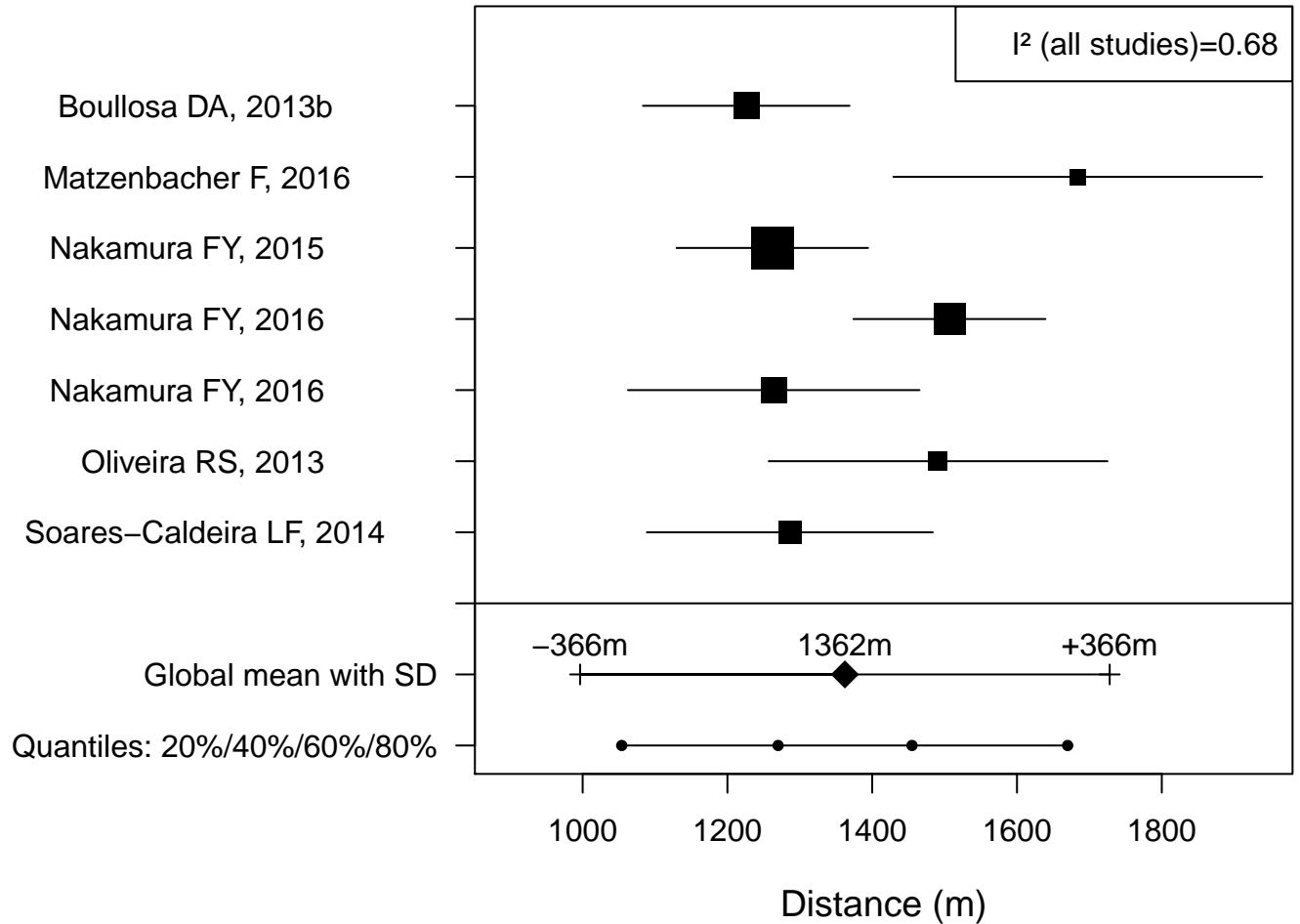

Supplement: Supplementary file 3 [file Data_Sheet_2.ZIP › Forestplots/Forestplot_YYIR1_Futsal_Male_Elite.pdf]

## Forestplot: YYIR1 Handball Female Elite

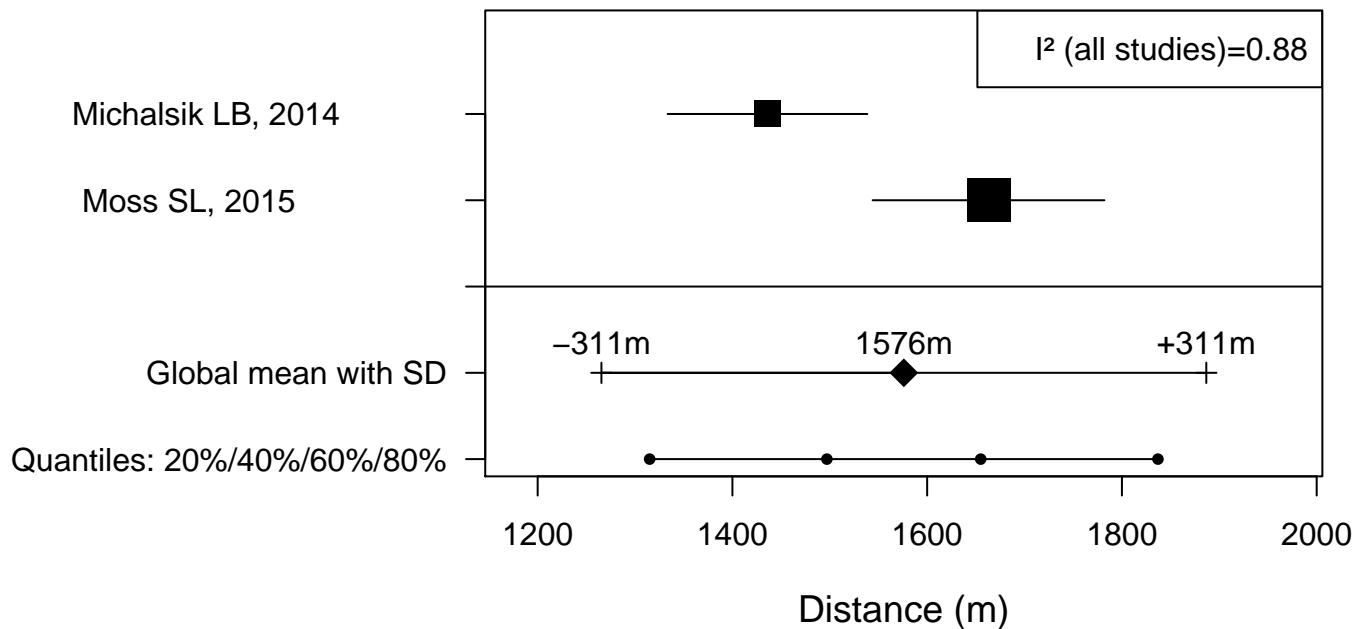

Supplement: Supplementary file 3 [file Data_Sheet_2.ZIP › Forestplots/Forestplot_YYIR1_Handball_Female_Elite.pdf]

## Forestplot: YYIR1 Handball Male Elite

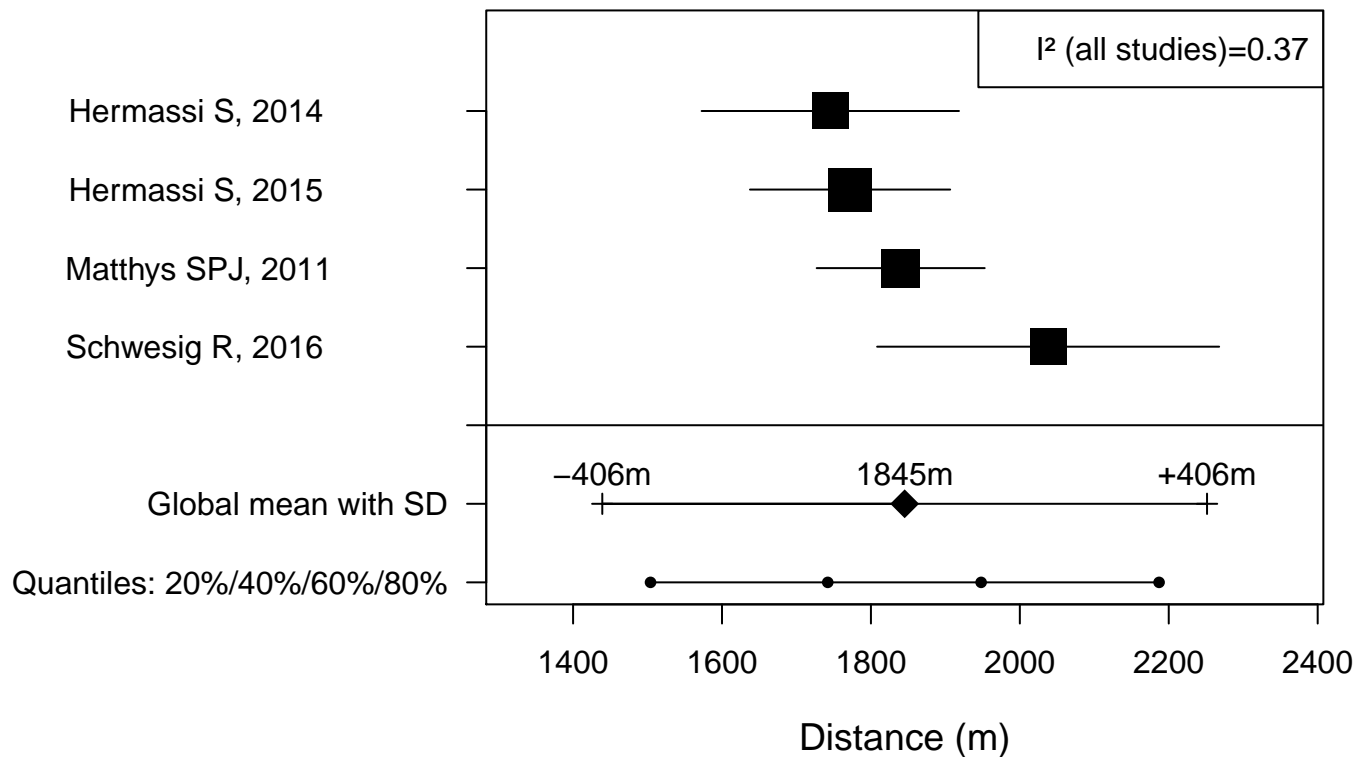

Supplement: Supplementary file 3 [file Data_Sheet_2.ZIP › Forestplots/Forestplot_YYIR1_Handball_Male_Elite.pdf]

## Forestplot: YYIR1 Handball Male Sub-Elite

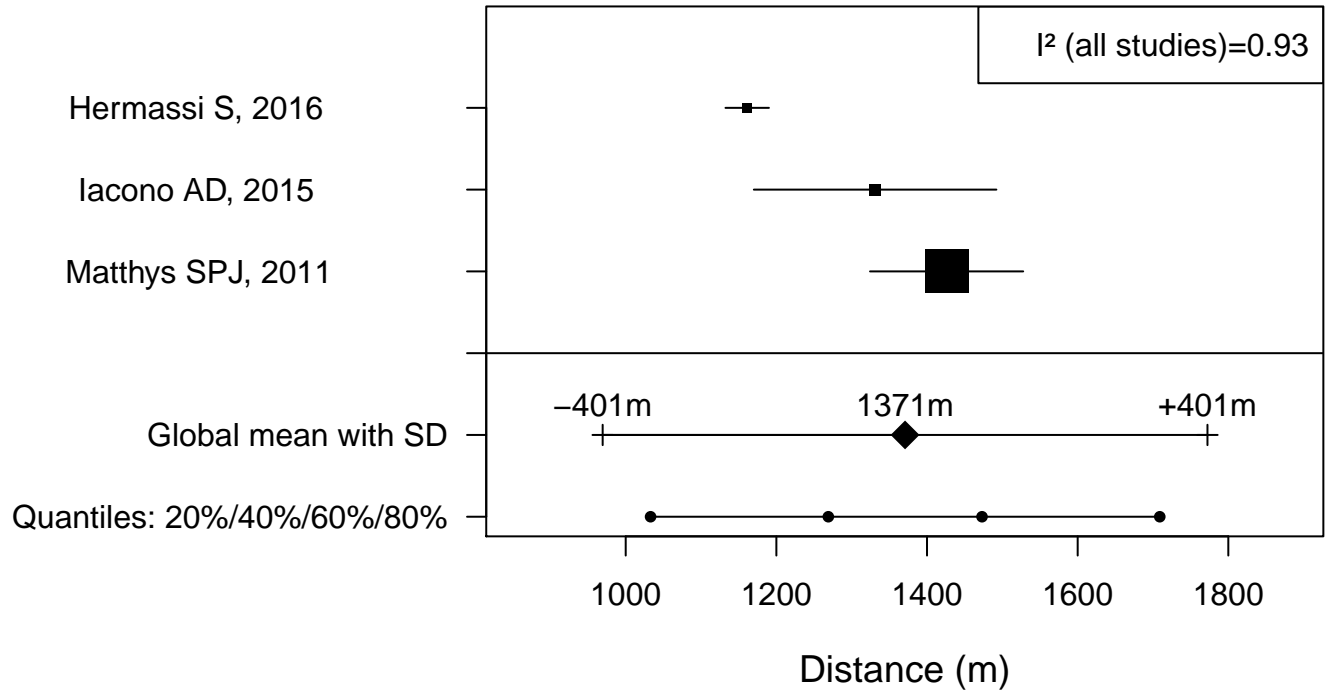

Supplement: Supplementary file 3 [file Data_Sheet_2.ZIP › Forestplots/Forestplot_YYIR1_Handball_Male_Sub-Elite.pdf]

## Forestplot: YYIR1 Hockey Female Elite

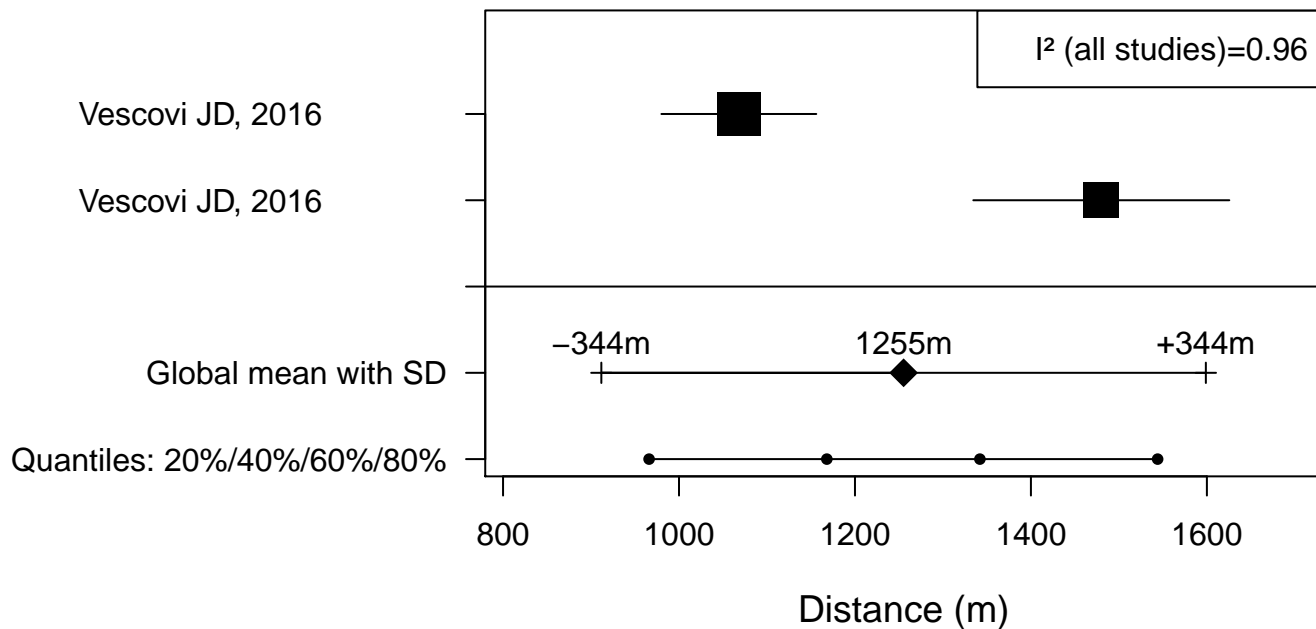

Supplement: Supplementary file 3 [file Data_Sheet_2.ZIP › Forestplots/Forestplot_YYIR1_Hockey_Female_Elite.pdf]

## Forestplot: YYIR1 Hockey Female Sub-Elite

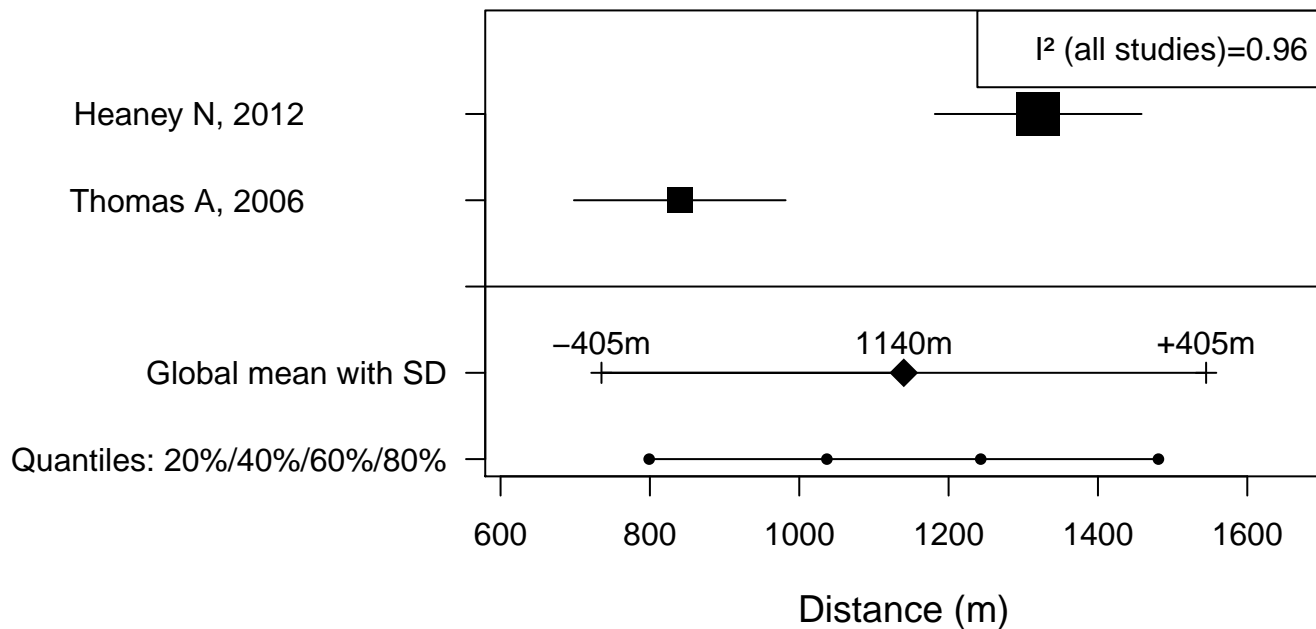

Supplement: Supplementary file 3 [file Data_Sheet_2.ZIP › Forestplots/Forestplot_YYIR1_Hockey_Female_Sub-Elite.pdf]

## Forestplot: YYIR1 Hurling Male Elite

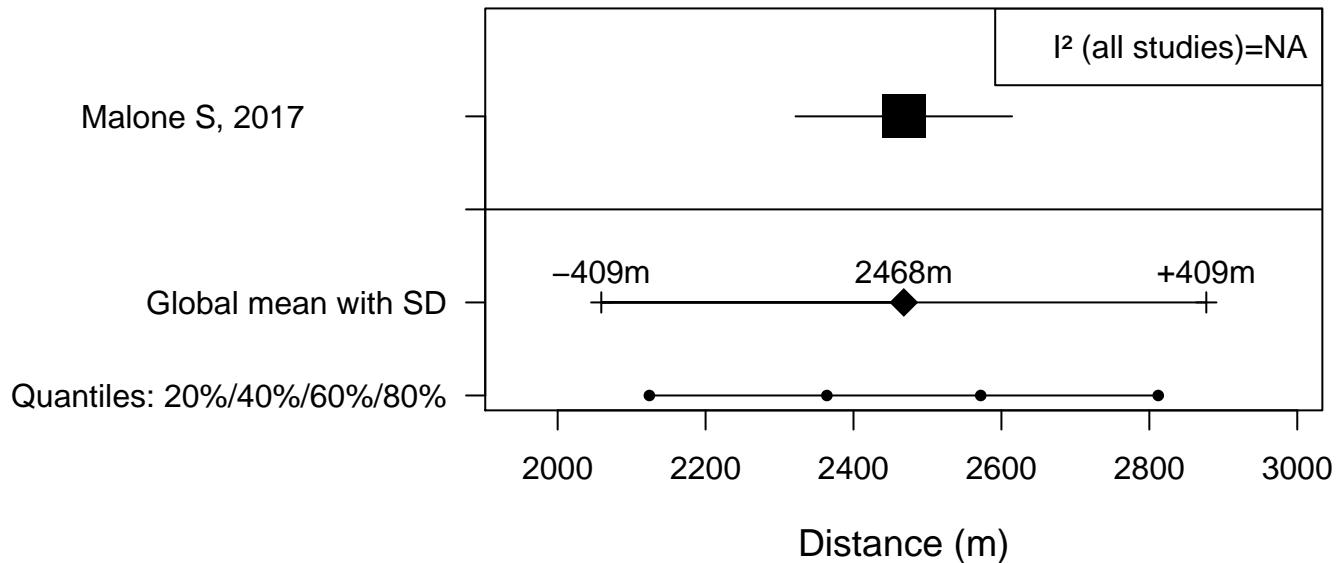

Supplement: Supplementary file 3 [file Data_Sheet_2.ZIP › Forestplots/Forestplot_YYIR1_Hurling_Male_Elite.pdf]

## Forestplot: YYIR1 Inactive Male

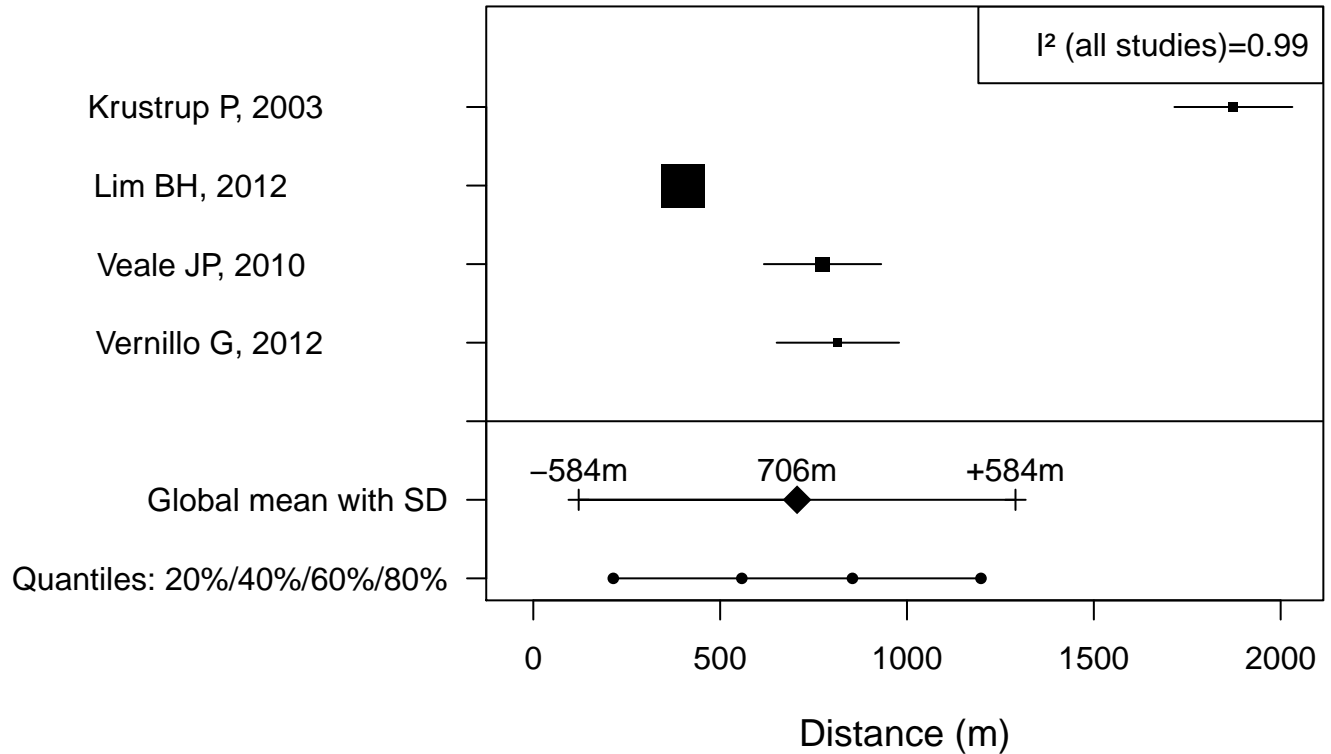

Supplement: Supplementary file 3 [file Data_Sheet_2.ZIP › Forestplots/Forestplot_YYIR1_Inactive_Male_.pdf]

## Forestplot: YYIR1 Netball Female Sub-Elite

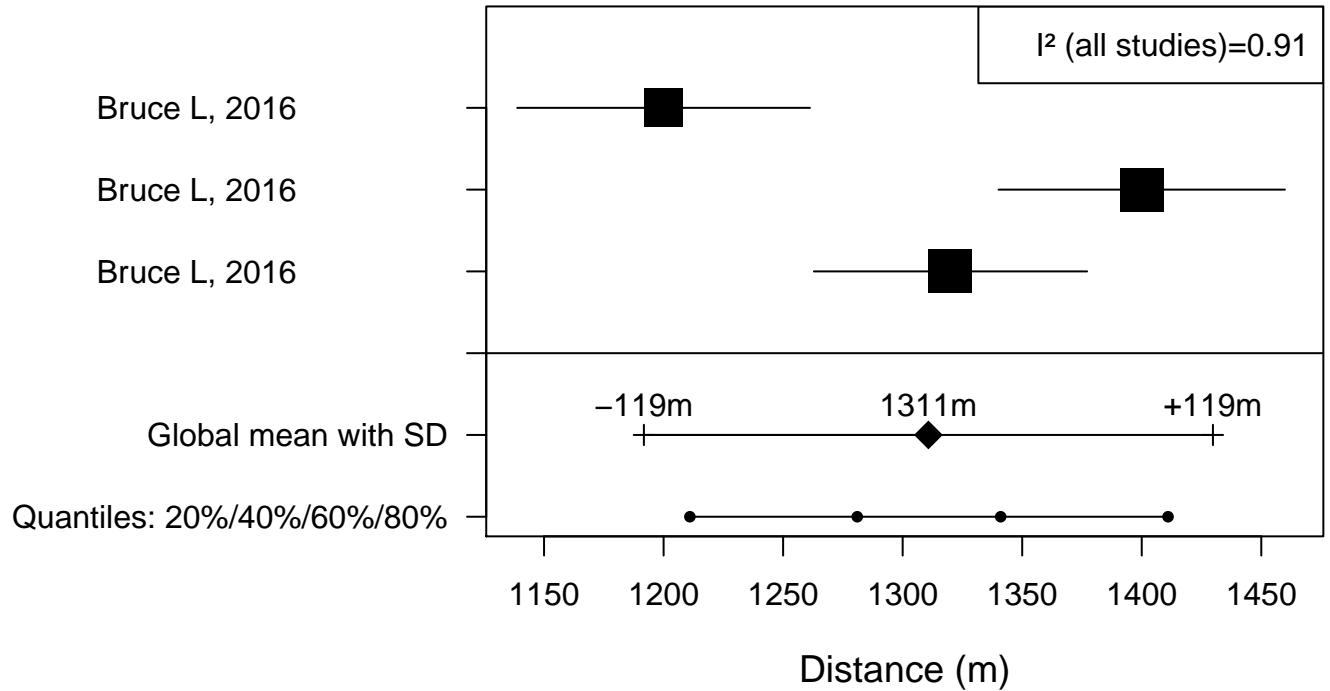

Supplement: Supplementary file 3 [file Data_Sheet_2.ZIP › Forestplots/Forestplot_YYIR1_Netball_Female_Sub-Elite.pdf]

## Forestplot: YYIR1 Recreational Female

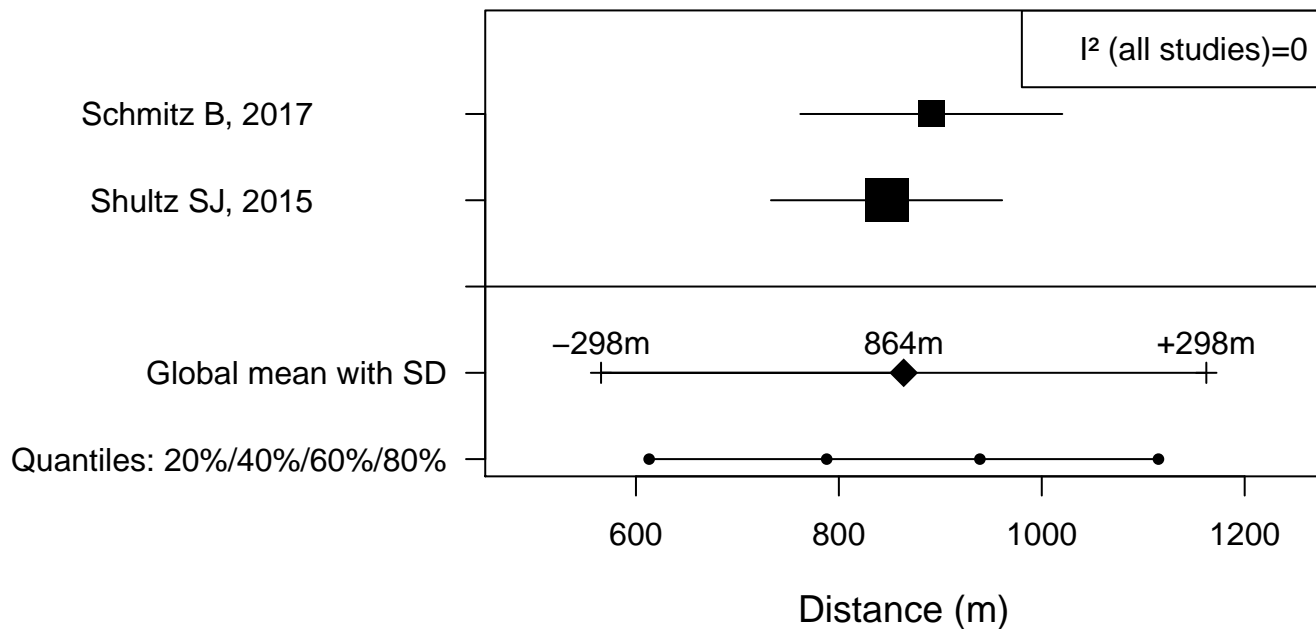

Supplement: Supplementary file 3 [file Data_Sheet_2.ZIP › Forestplots/Forestplot_YYIR1_Recreational_Female_.pdf]

# Forestplot: YYIR1 Recreational Male

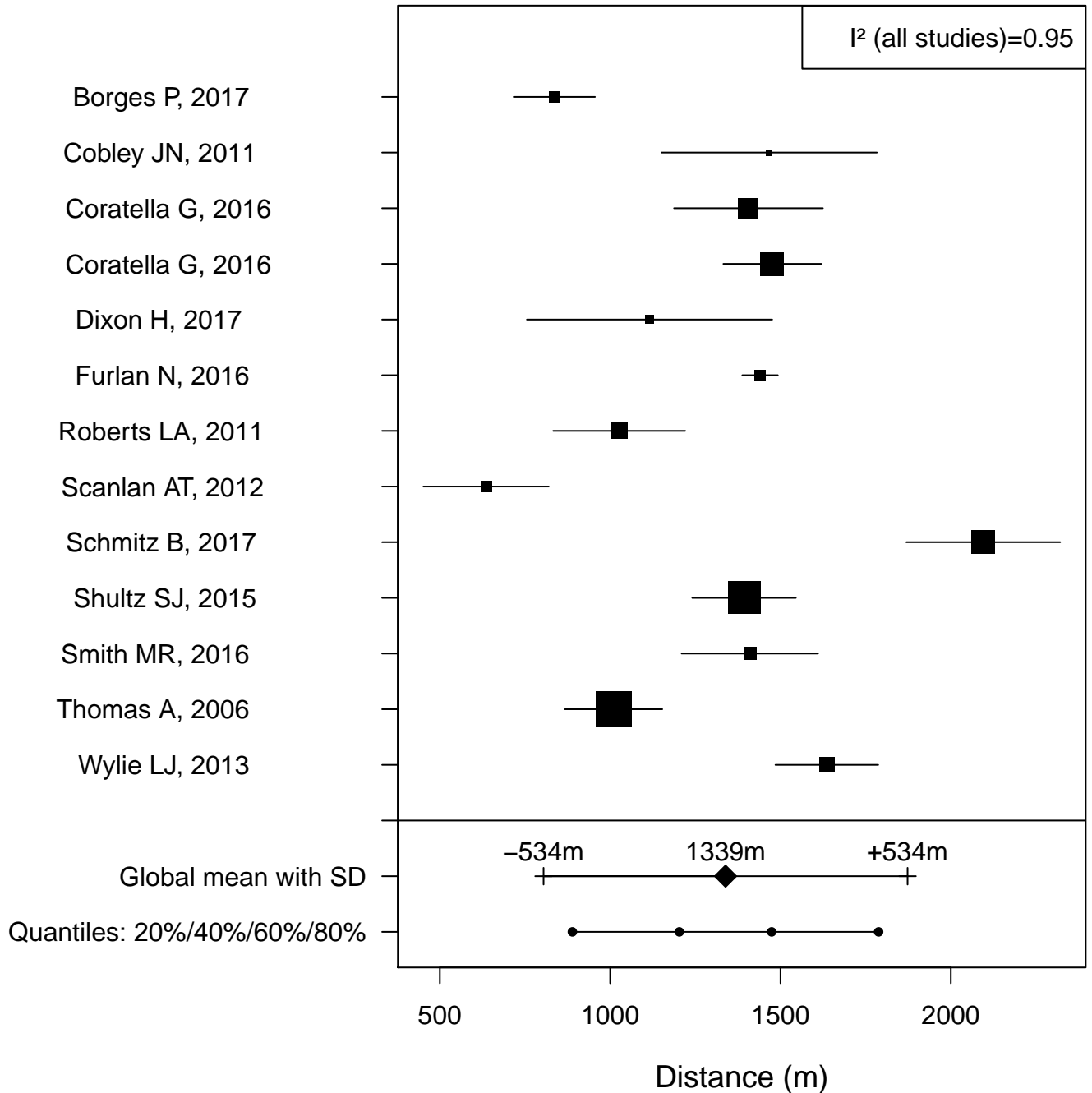

Supplement: Supplementary file 3 [file Data_Sheet_2.ZIP › Forestplots/Forestplot_YYIR1_Recreational_Male_.pdf]

## Forestplot: YYIR1 Referees Female Sub-Elite

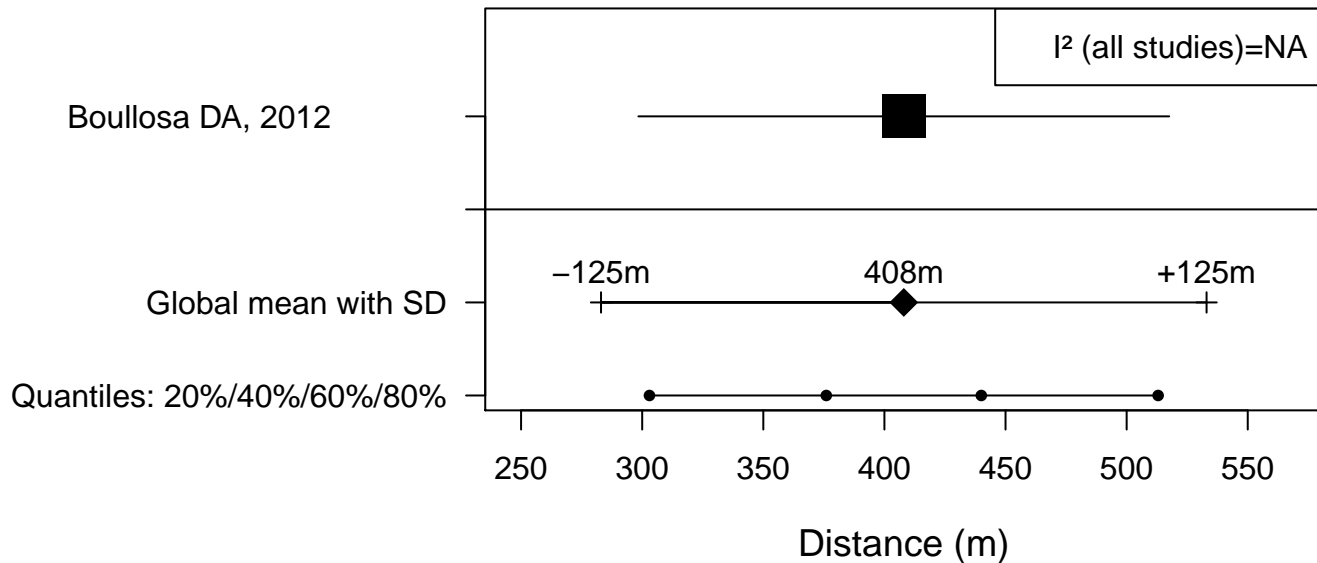

Supplement: Supplementary file 3 [file Data_Sheet_2.ZIP › Forestplots/Forestplot_YYIR1_Referees_Female_Sub-Elite.pdf]

## Forestplot: YYIR1 Referees Male Amateur

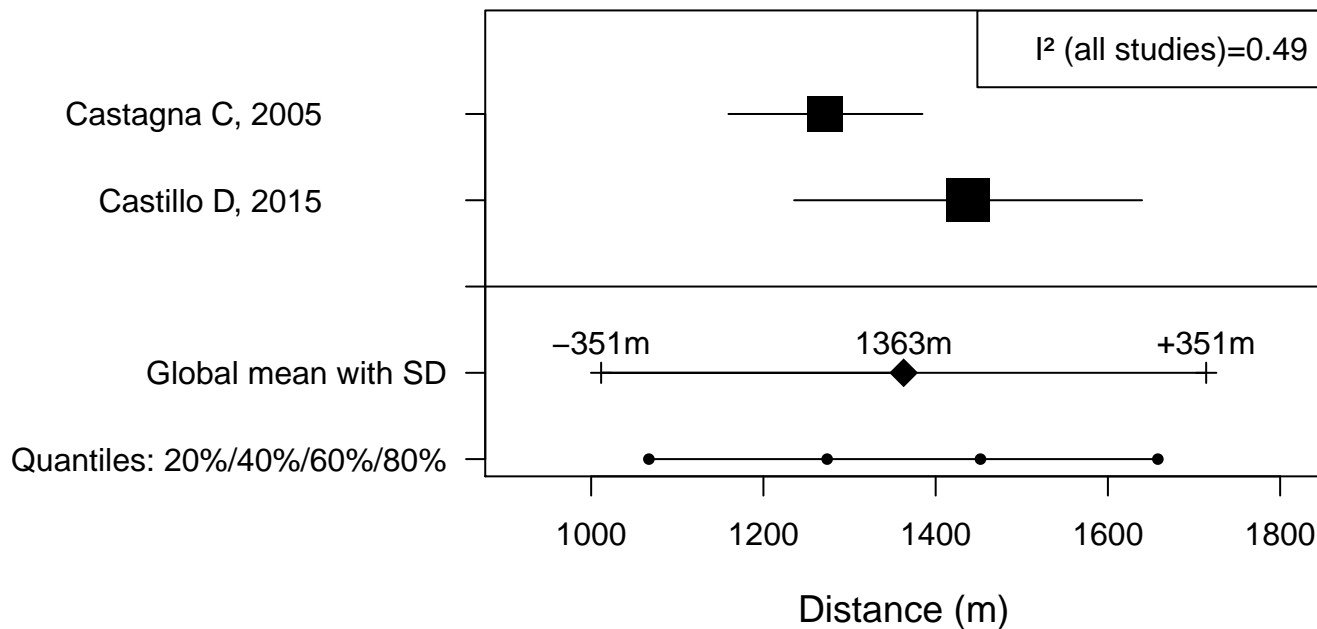

Supplement: Supplementary file 3 [file Data_Sheet_2.ZIP › Forestplots/Forestplot_YYIR1_Referees_Male_Amateur.pdf]

# Forestplot: YYIR1 Referees Male Elite

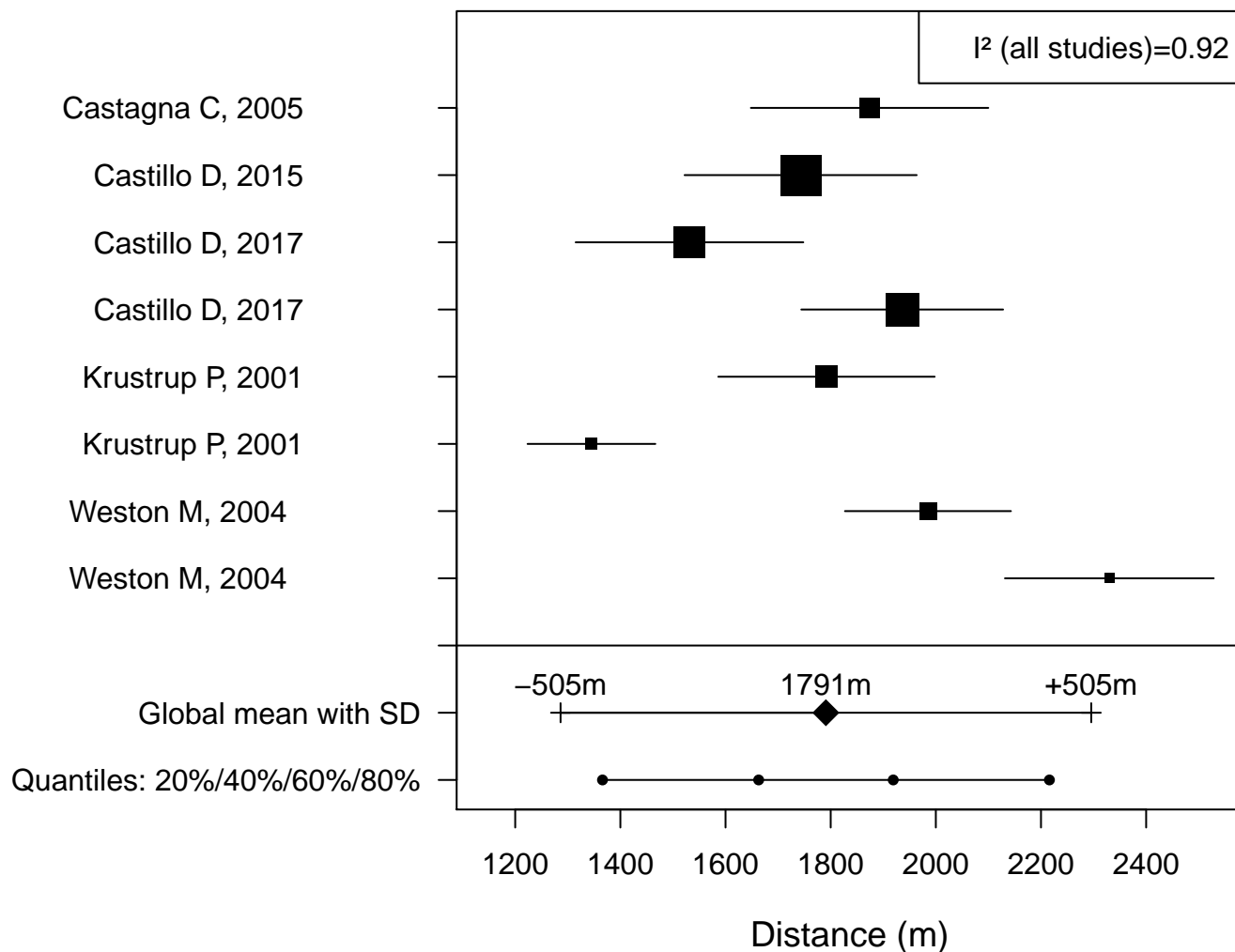

Supplement: Supplementary file 3 [file Data_Sheet_2.ZIP › Forestplots/Forestplot_YYIR1_Referees_Male_Elite.pdf]

## Forestplot: YYIR1 Referees Male Sub-Elite

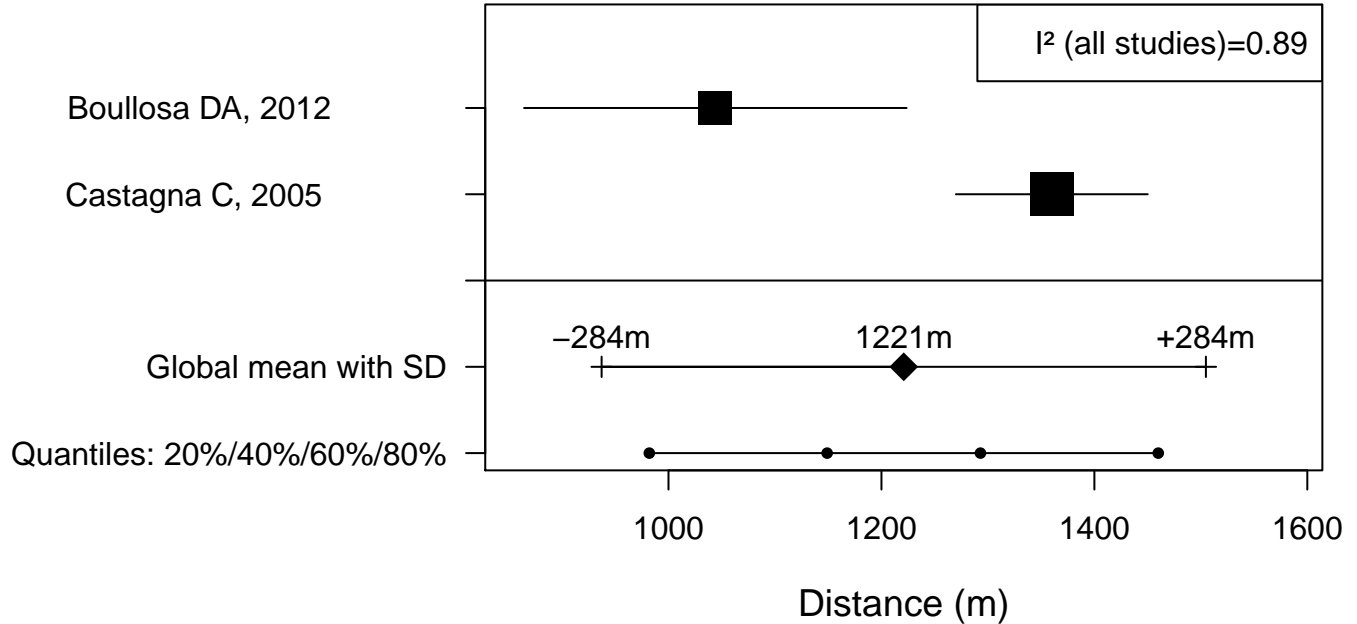

Supplement: Supplementary file 3 [file Data_Sheet_2.ZIP › Forestplots/Forestplot_YYIR1_Referees_Male_Sub-Elite.pdf]

Forestplot: YYIR1 Rugby or GF Male Sub-Elite

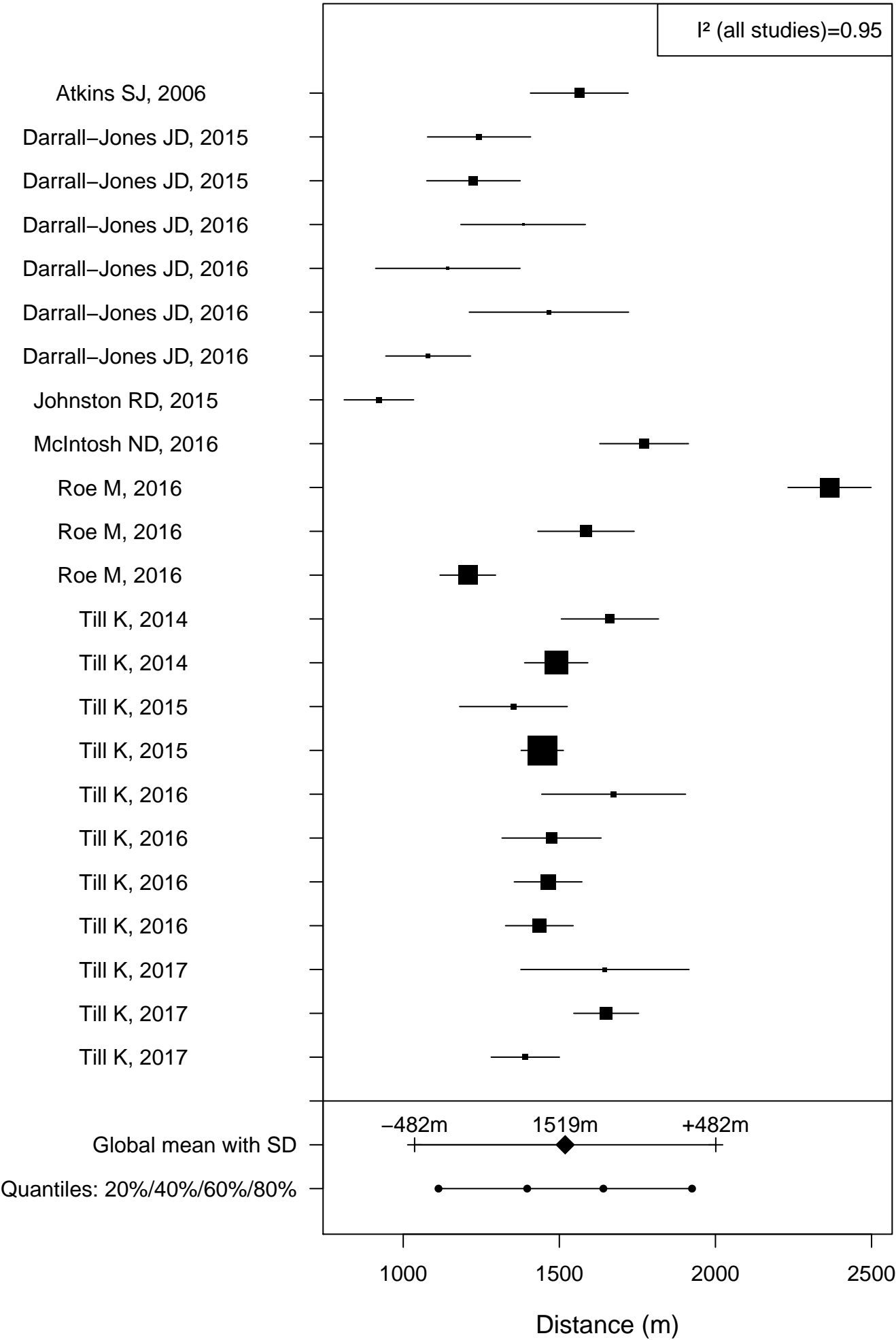

Supplement: Supplementary file 3 [file Data_Sheet_2.ZIP › Forestplots/Forestplot_YYIR1_Rugby or GF_Male_Sub-Elite.pdf]

# Forestplot: YYIR1 Rugby or RS or GF Male Elite

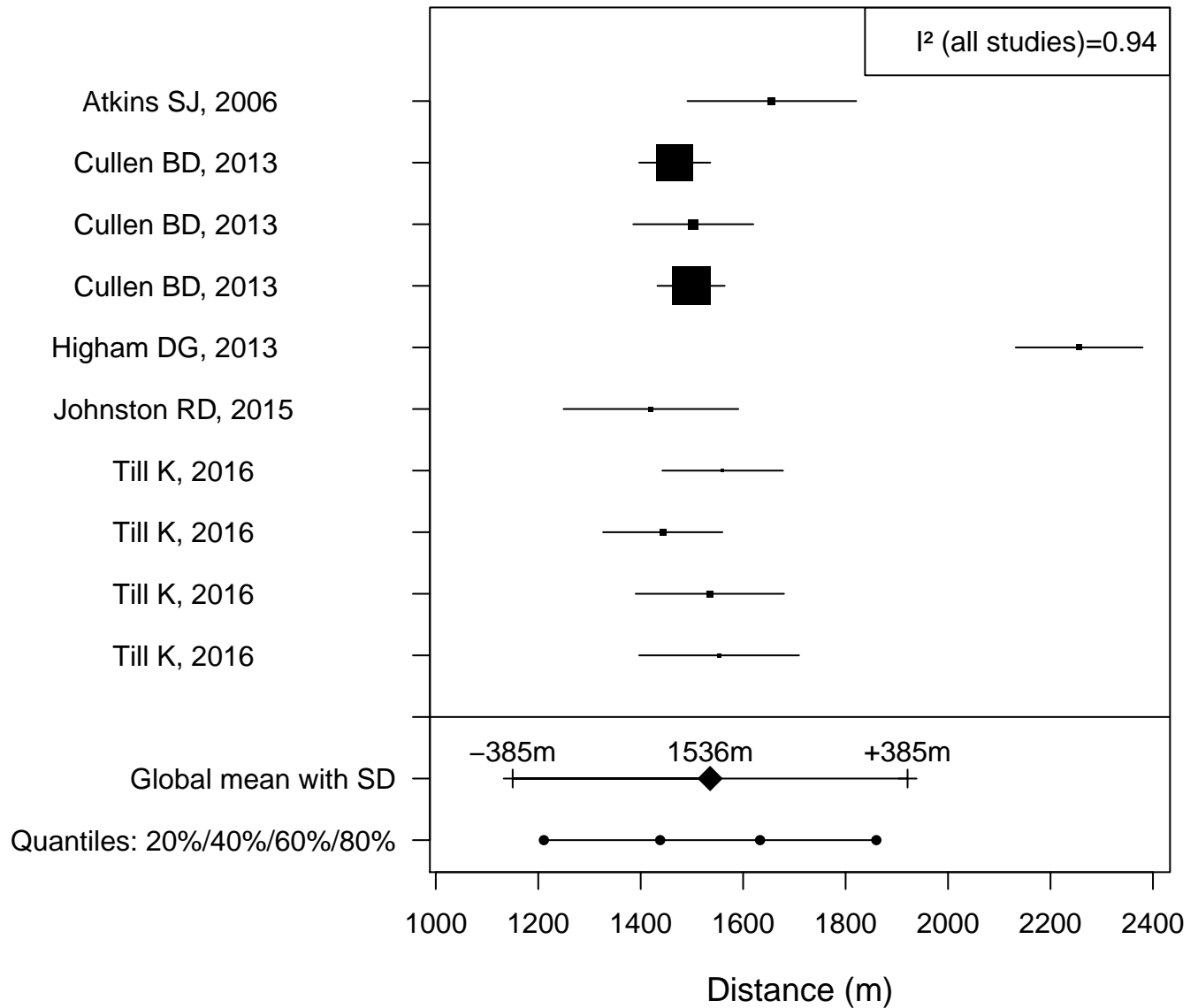

Supplement: Supplementary file 3 [file Data_Sheet_2.ZIP › Forestplots/Forestplot_YYIR1_Rugby or RS or GF_Male_Elite.pdf]

## Forestplot: YYIR1 Rugby or RS Female Elite

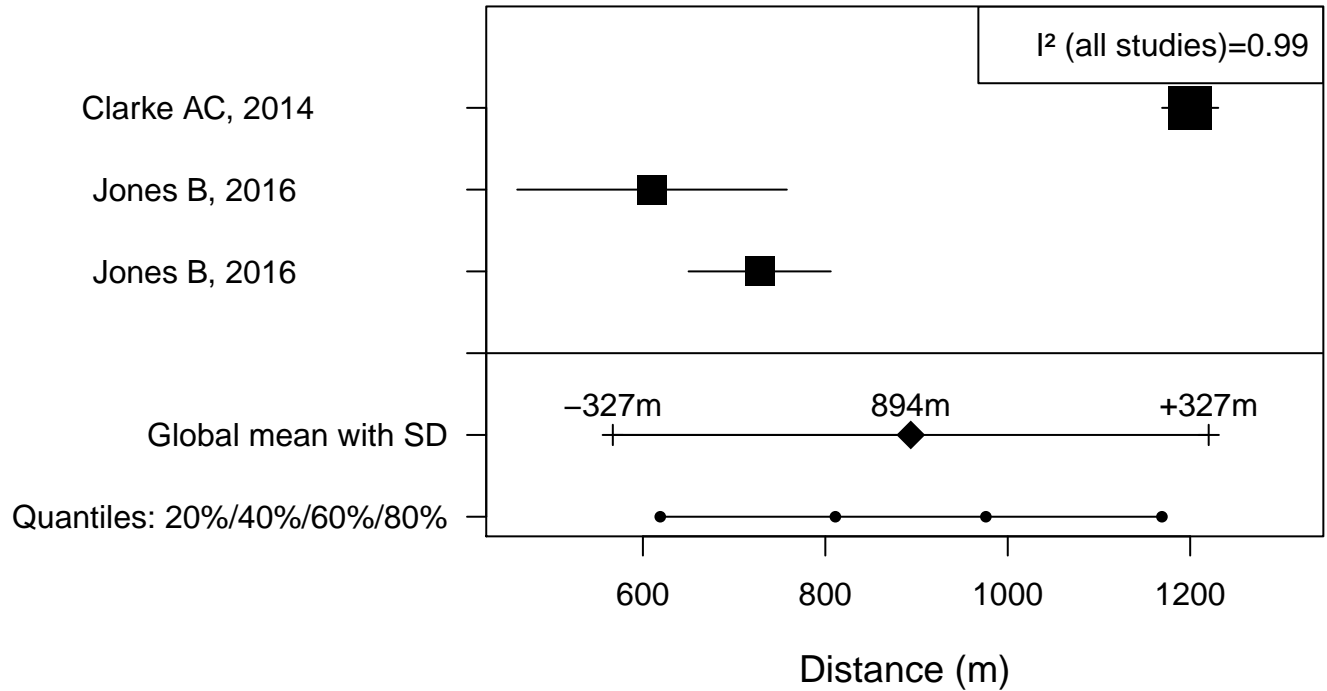

Supplement: Supplementary file 3 [file Data_Sheet_2.ZIP › Forestplots/Forestplot_YYIR1_Rugby or RS_Female_Elite.pdf]

## Forestplot: YYIR1 Rugby Male Amateur

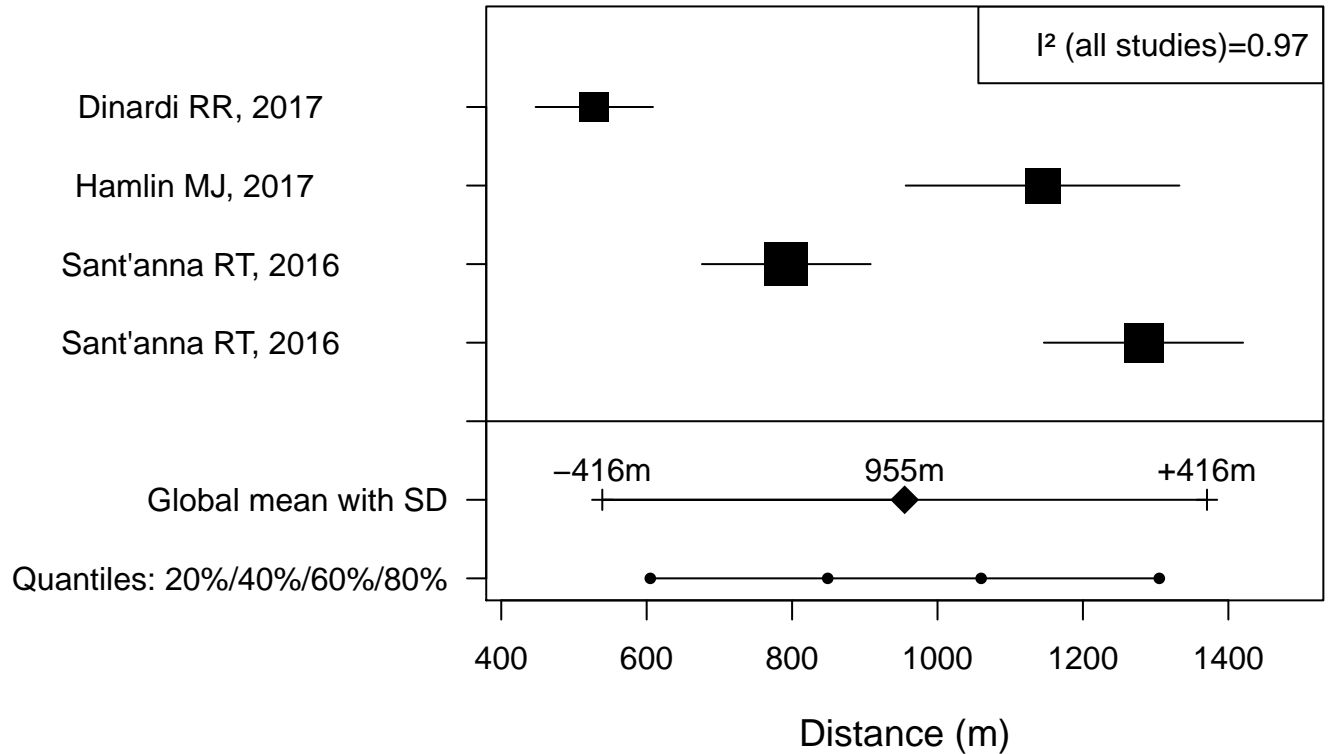

Supplement: Supplementary file 3 [file Data_Sheet_2.ZIP › Forestplots/Forestplot_YYIR1_Rugby_Male_Amateur.pdf]

## Forestplot: YYIR1 Soccer Female Amateur

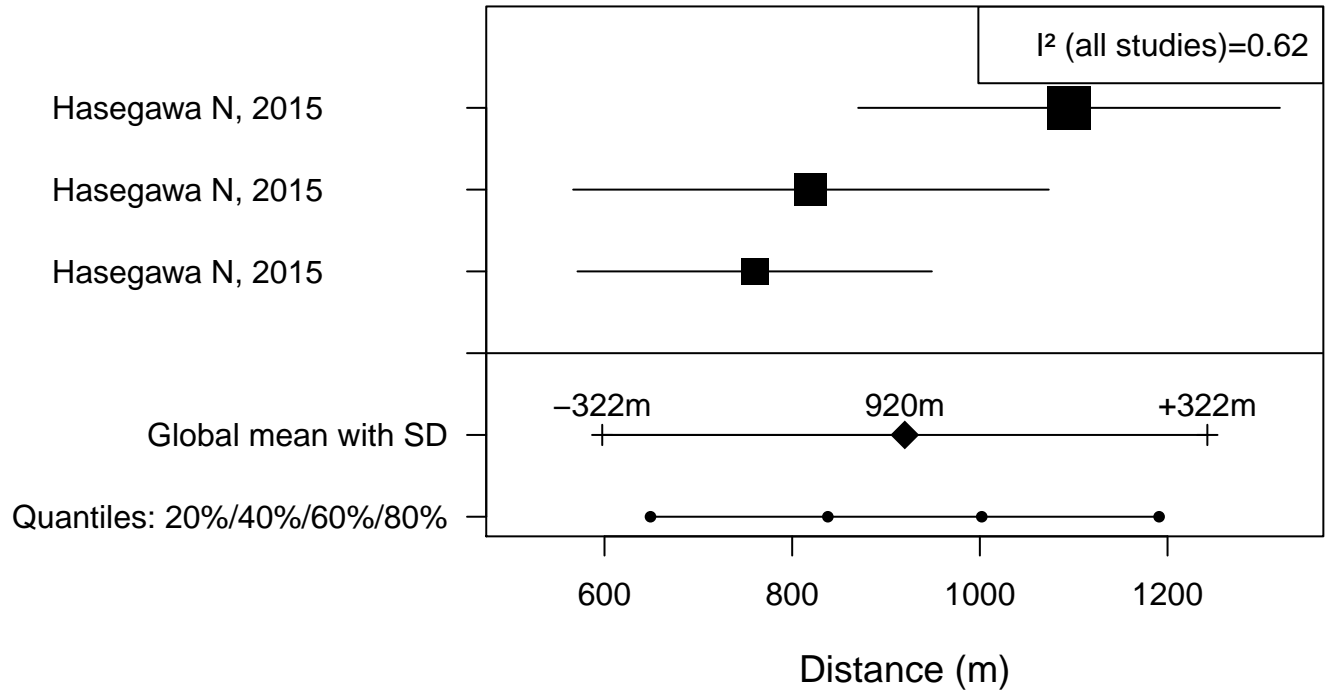

Supplement: Supplementary file 3 [file Data_Sheet_2.ZIP › Forestplots/Forestplot_YYIR1_Soccer_Female_Amateur.pdf]

# Forestplot: YYIR1 Soccer Female Sub-Elite or Elite

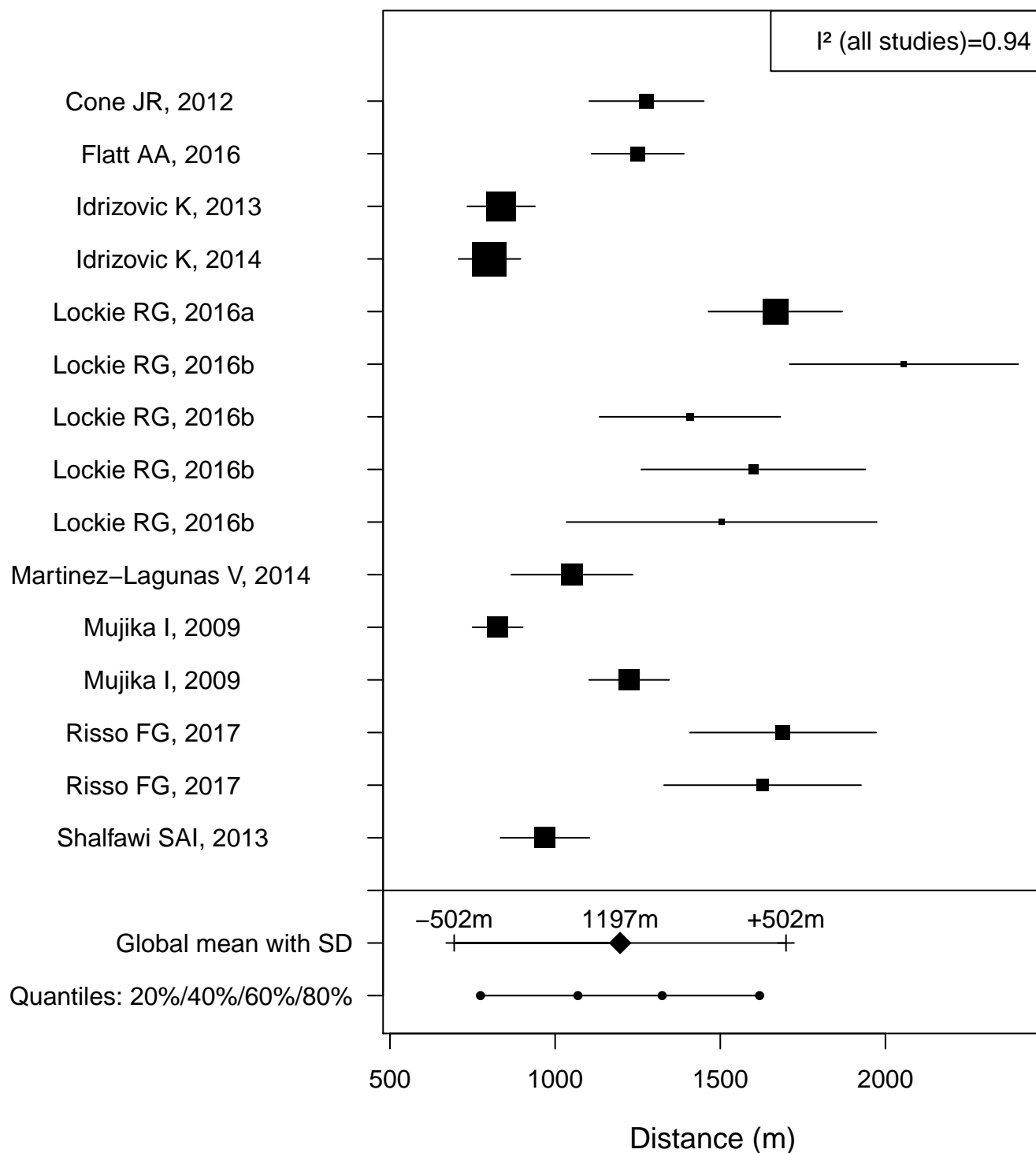

Supplement: Supplementary file 3 [file Data_Sheet_2.ZIP › Forestplots/Forestplot_YYIR1_Soccer_Female_Sub-Elite or Elite.pdf]

Forestplot: YYIR1 Soccer Male Amateur

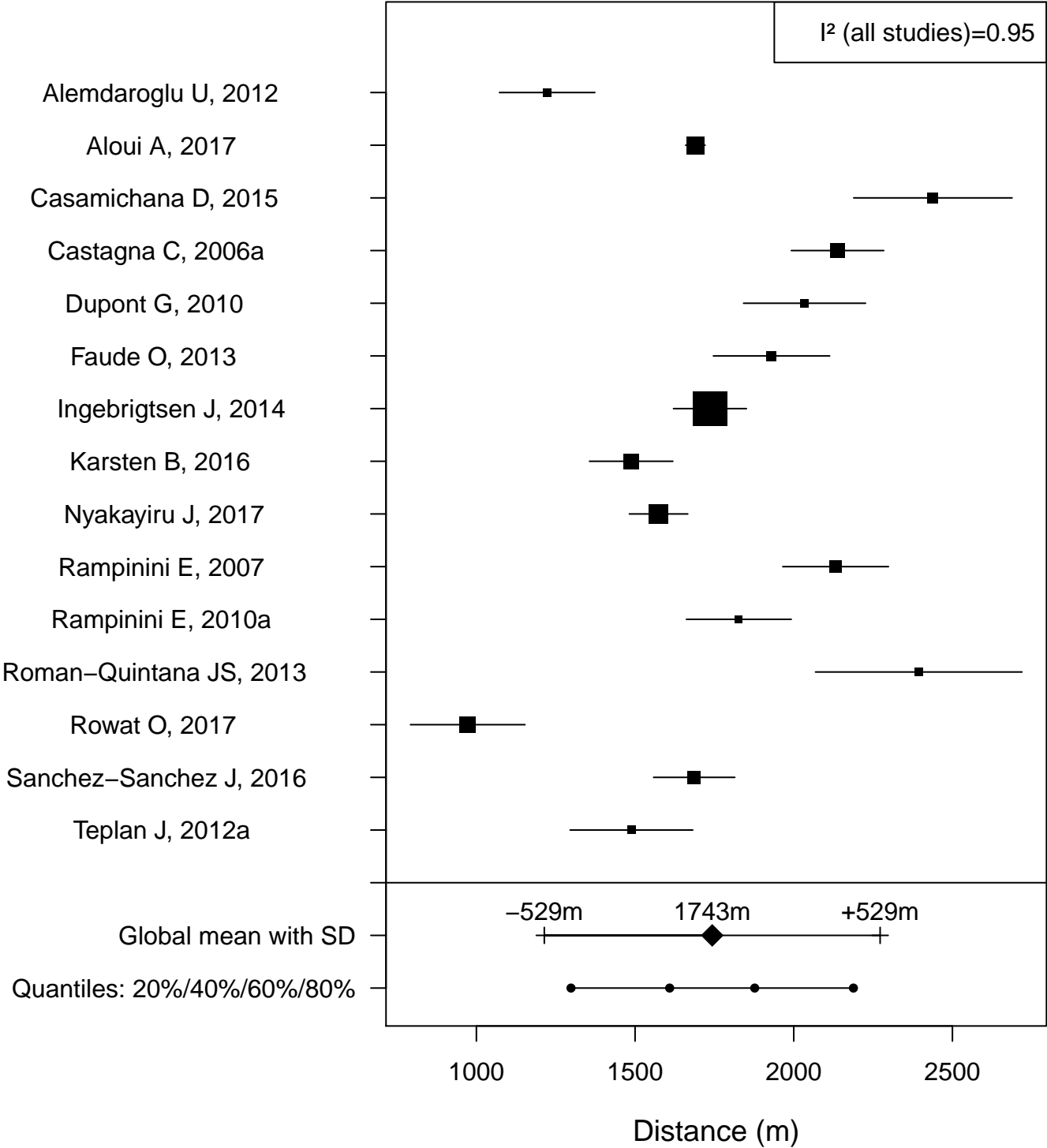

Supplement: Supplementary file 3 [file Data_Sheet_2.ZIP › Forestplots/Forestplot_YYIR1_Soccer_Male_Amateur.pdf]

# Forestplot: YYIR1 Soccer Male Elite

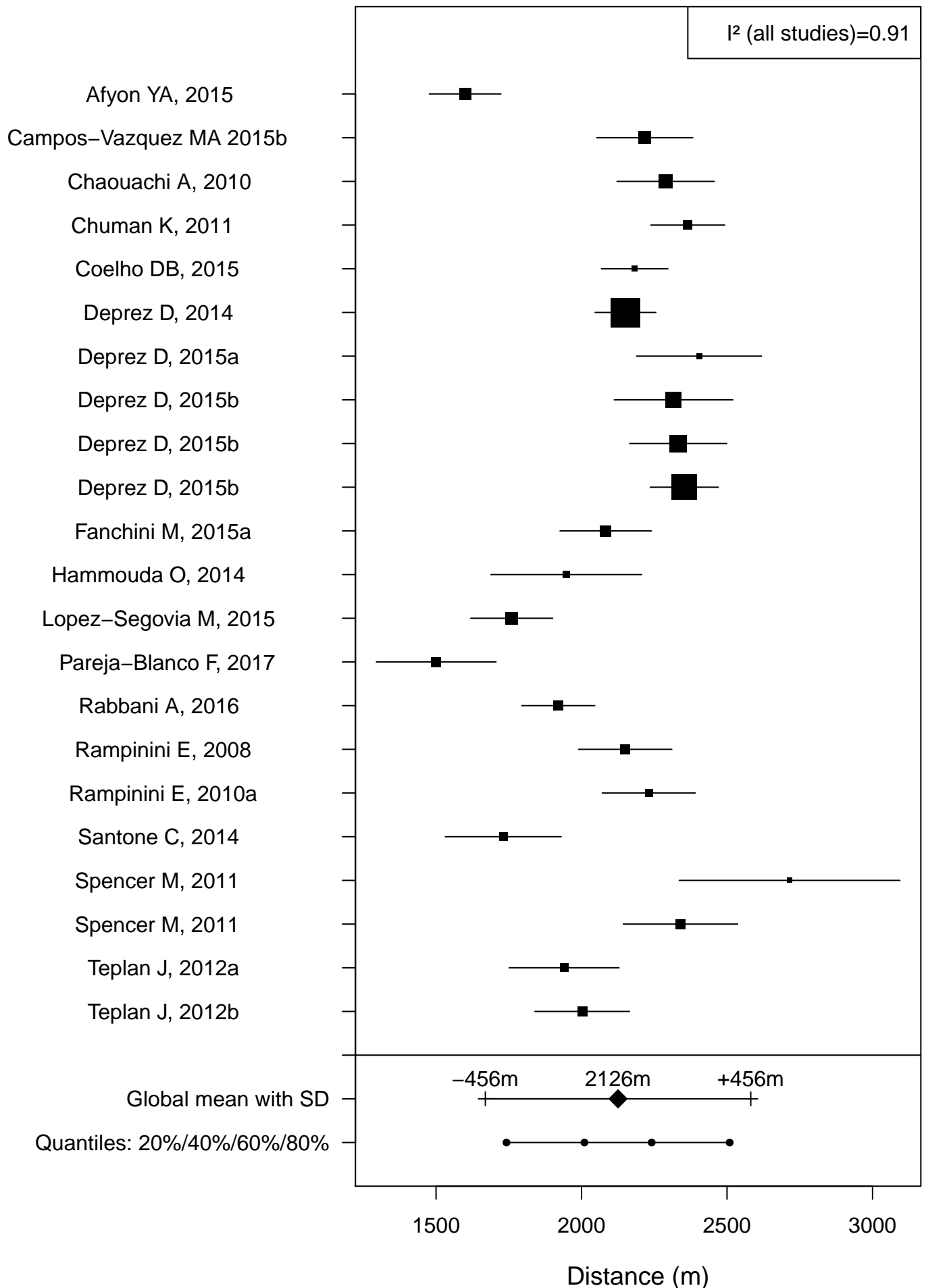

Supplement: Supplementary file 3 [file Data_Sheet_2.ZIP › Forestplots/Forestplot_YYIR1_Soccer_Male_Elite.pdf]

Forestplot: YYIR1 Soccer Male Sub-Elite

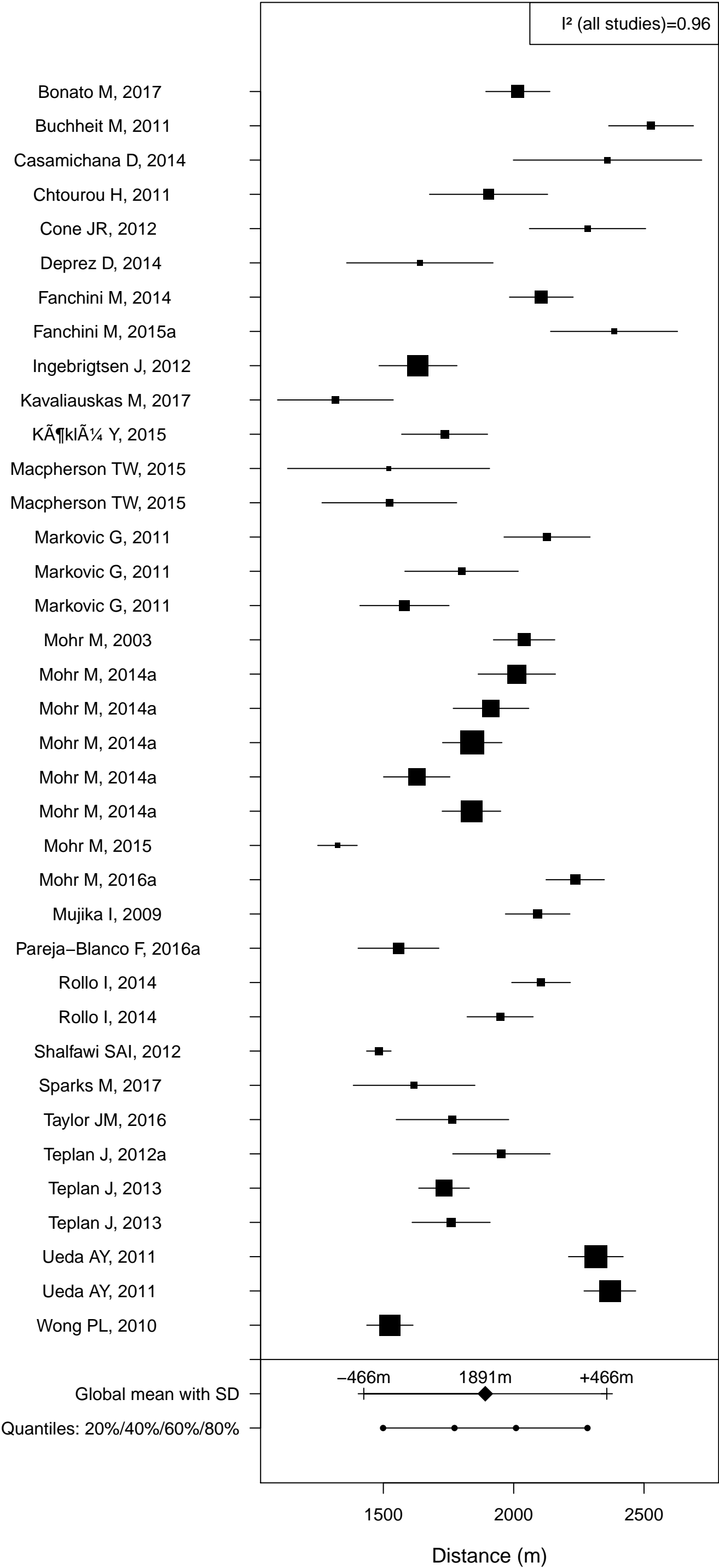

Supplement: Supplementary file 3 [file Data_Sheet_2.ZIP › Forestplots/Forestplot_YYIR1_Soccer_Male_Sub-Elite.pdf]

# Forestplot: YYIR1 Soccer Male Top-Elite

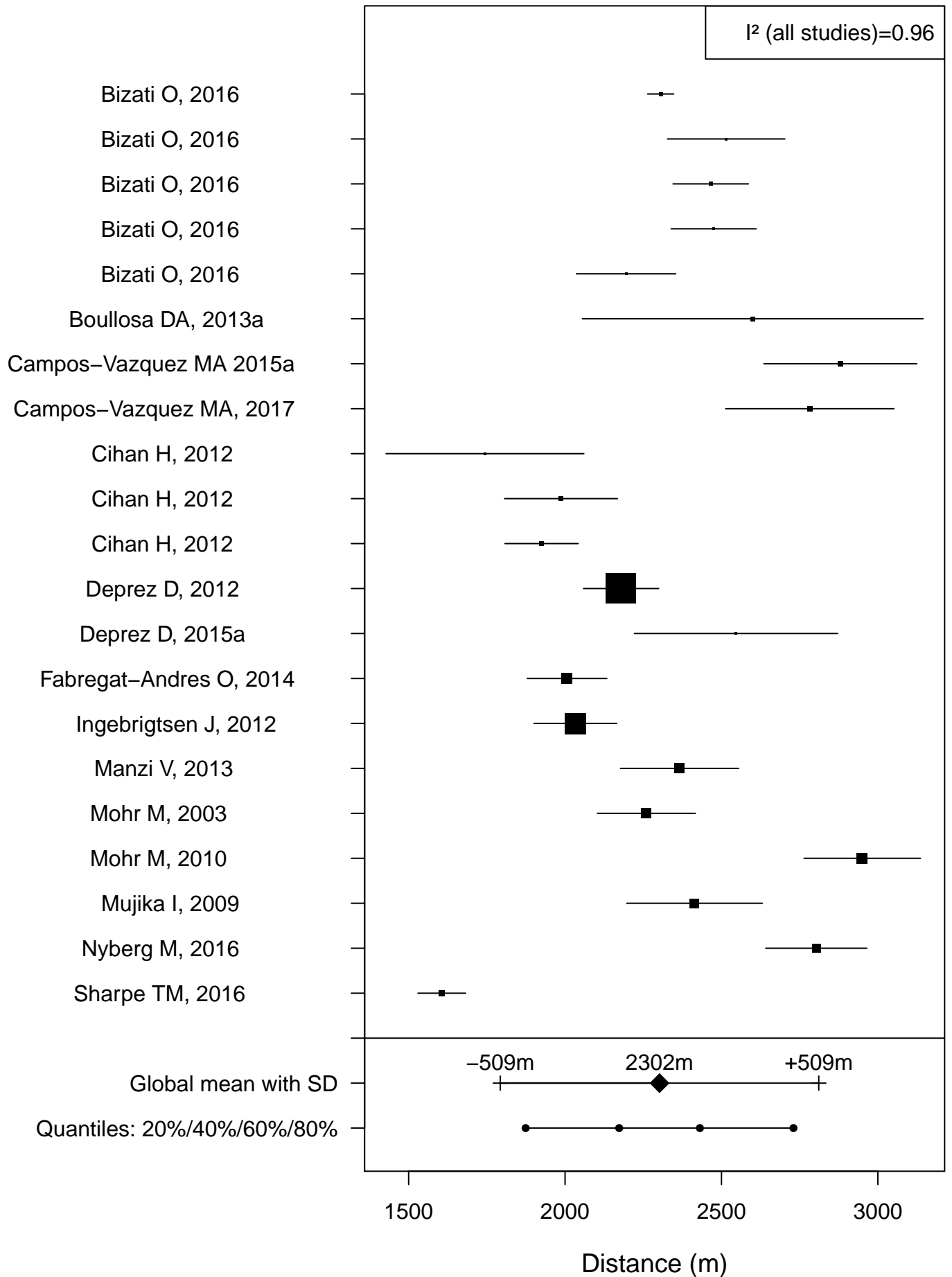

Supplement: Supplementary file 3 [file Data_Sheet_2.ZIP › Forestplots/Forestplot_YYIR1_Soccer_Male_Top-Elite.pdf]

## Forestplot: YYIR1 Tennis Male Elite

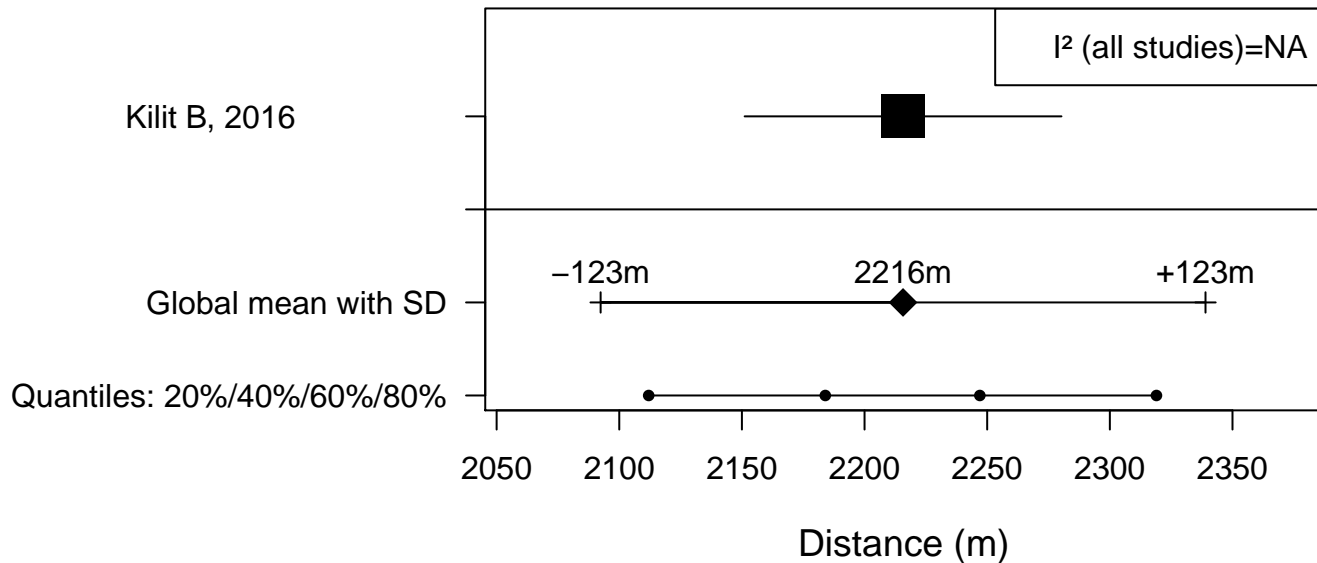

Supplement: Supplementary file 3 [file Data_Sheet_2.ZIP › Forestplots/Forestplot_YYIR1_Tennis_Male_Elite.pdf]

## Forestplot: YYIR1 Ultimate Frisbee Male Sub-Elite

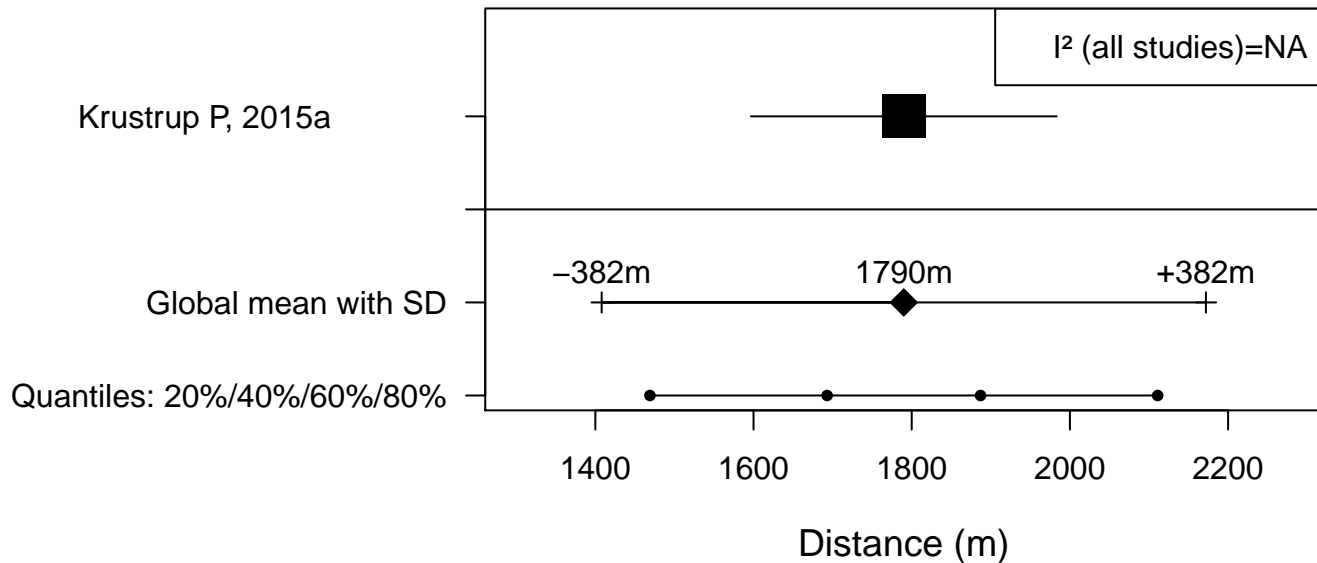

Supplement: Supplementary file 3 [file Data_Sheet_2.ZIP › Forestplots/Forestplot_YYIR1_Ultimate Frisbee_Male_Sub-Elite.pdf]

## Forestplot: YYIR1 Volleyball Female Elite

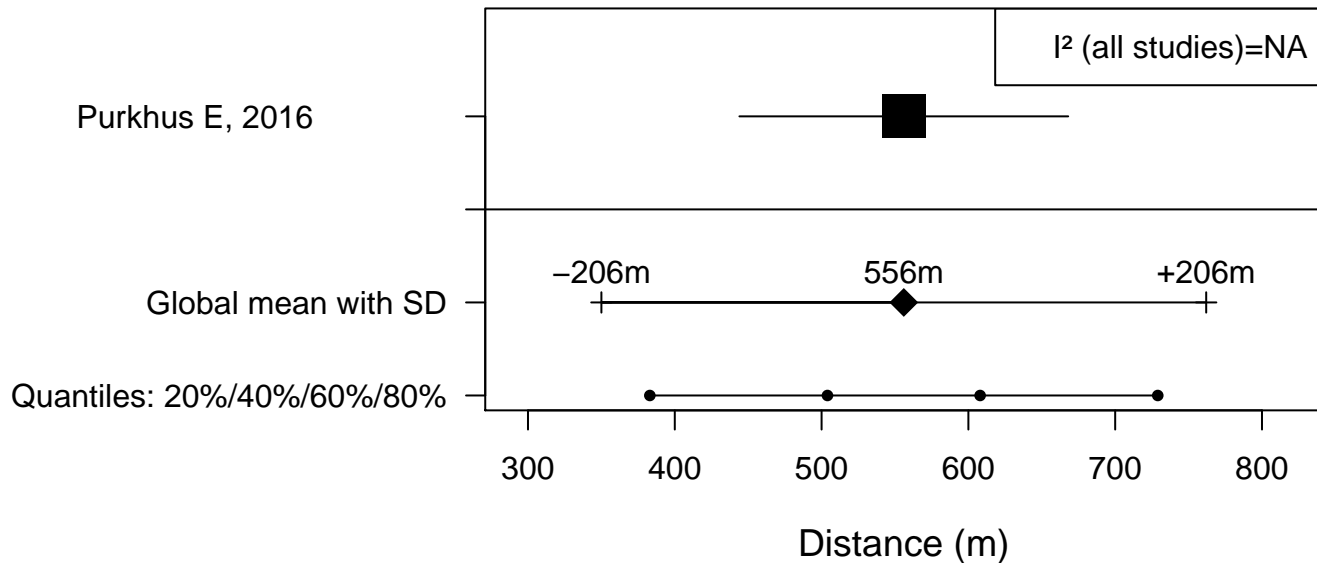

Supplement: Supplementary file 3 [file Data_Sheet_2.ZIP › Forestplots/Forestplot_YYIR1_Volleyball_Female_Elite.pdf]

## Forestplot: YYIR1 X-country Skiing Male Amateur

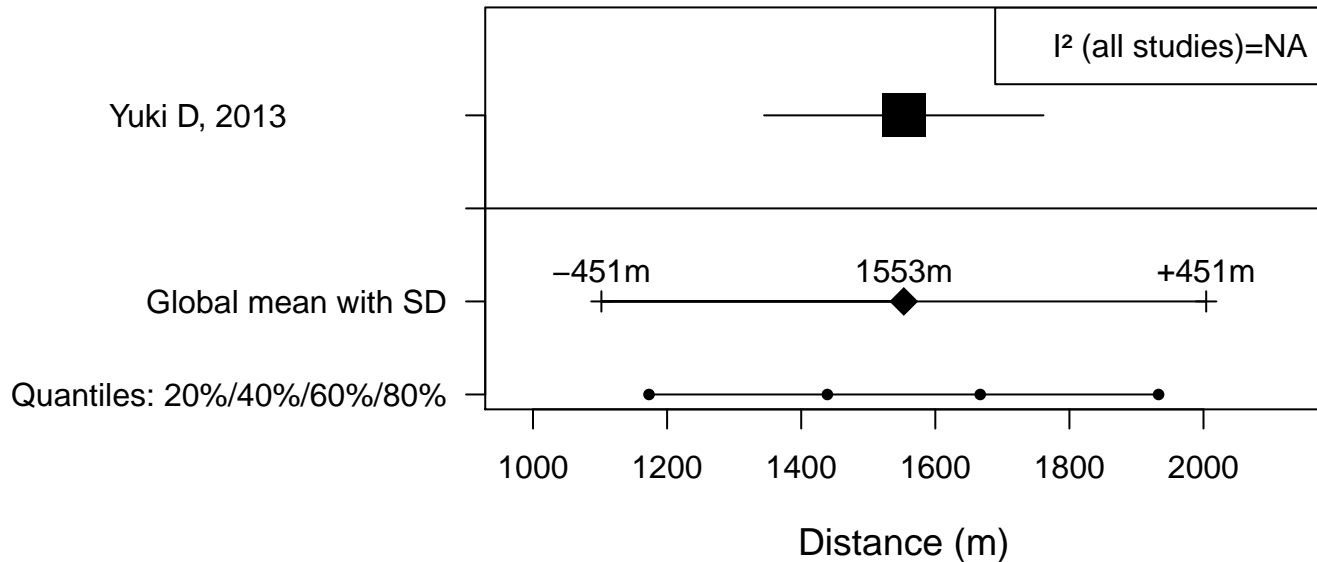

Supplement: Supplementary file 3 [file Data_Sheet_2.ZIP › Forestplots/Forestplot_YYIR1_X-country Skiing_Male_Amateur.pdf]

## Forestplot: YYIR1 X-country Skiing Male Elite

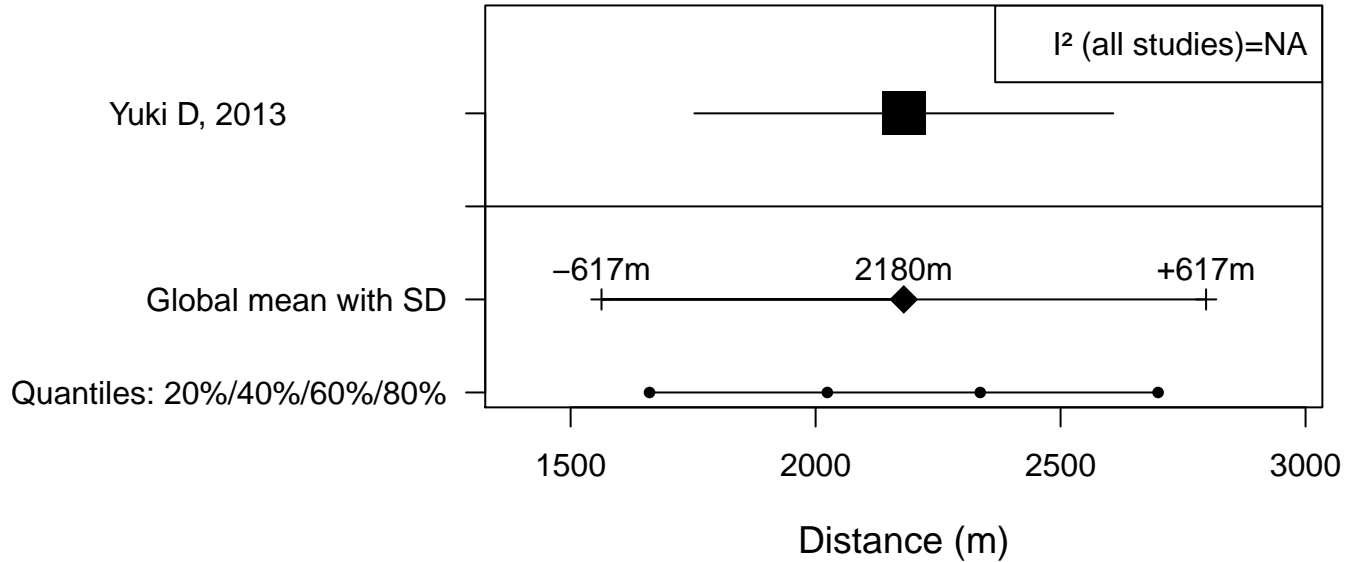

Supplement: Supplementary file 3 [file Data_Sheet_2.ZIP › Forestplots/Forestplot_YYIR1_X-country Skiing_Male_Elite.pdf]

## Forestplot: YYIR2 Basketball Female Elite

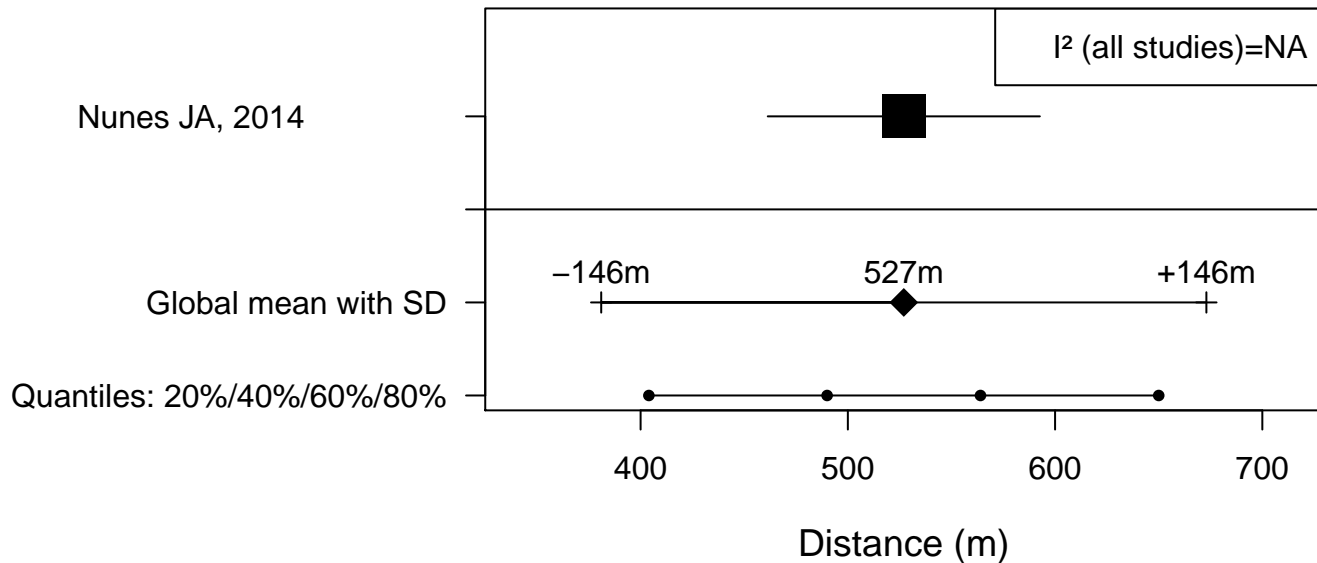

Supplement: Supplementary file 3 [file Data_Sheet_2.ZIP › Forestplots/Forestplot_YYIR2_Basketball_Female_Elite.pdf]

## Forestplot: YYIR2 Basketball Male Elite

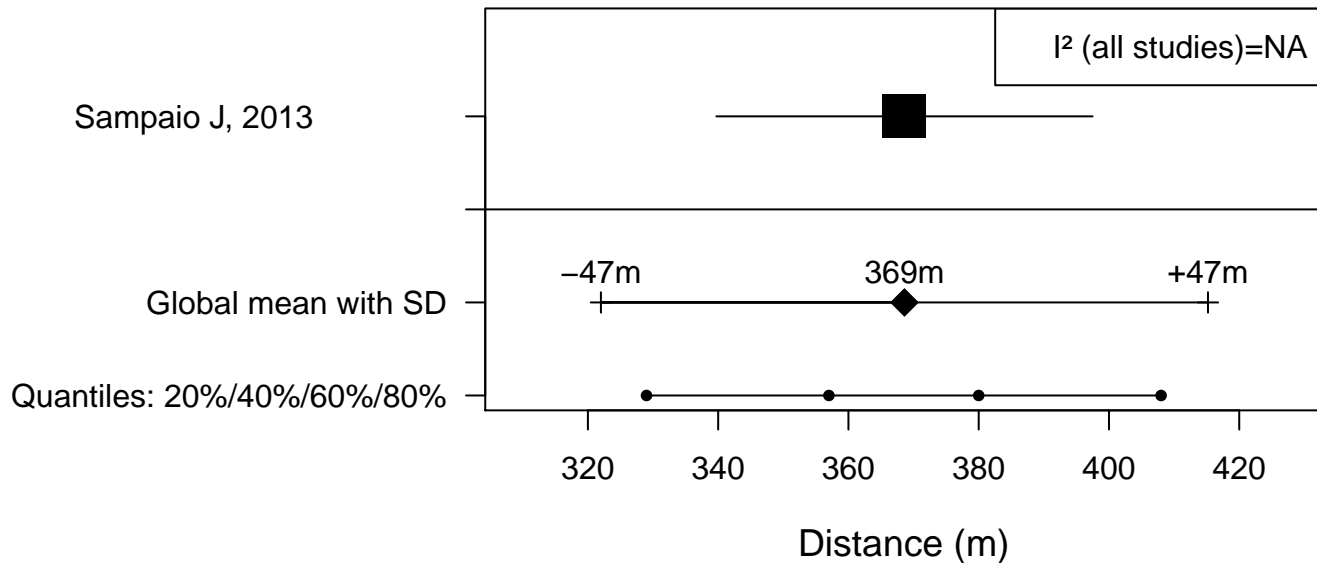

Supplement: Supplementary file 3 [file Data_Sheet_2.ZIP › Forestplots/Forestplot_YYIR2_Basketball_Male_Elite.pdf]

## Forestplot: YYIR2 Endurance Runners Male Amateur

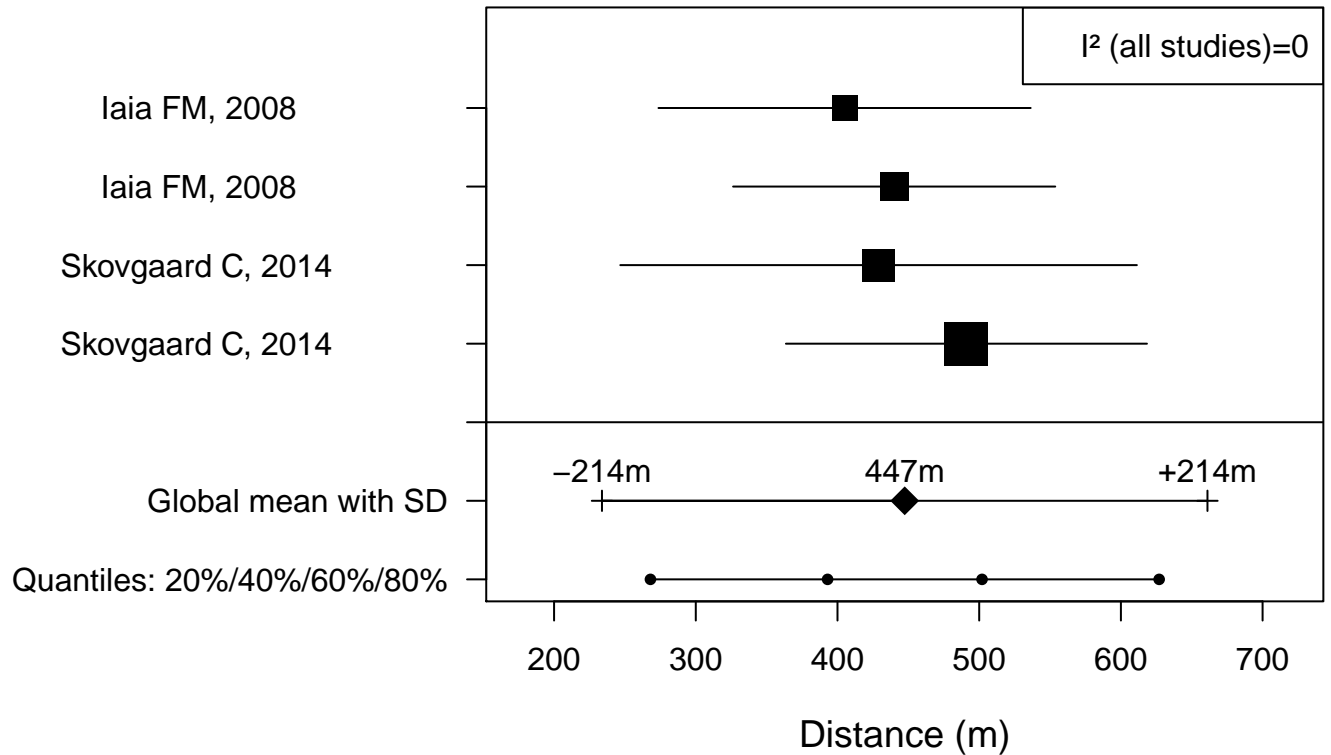

Supplement: Supplementary file 3 [file Data_Sheet_2.ZIP › Forestplots/Forestplot_YYIR2_Endurance Runners_Male_Amateur.pdf]

## Forestplot: YYIR2 Football Male Amateur or Sub-Elite

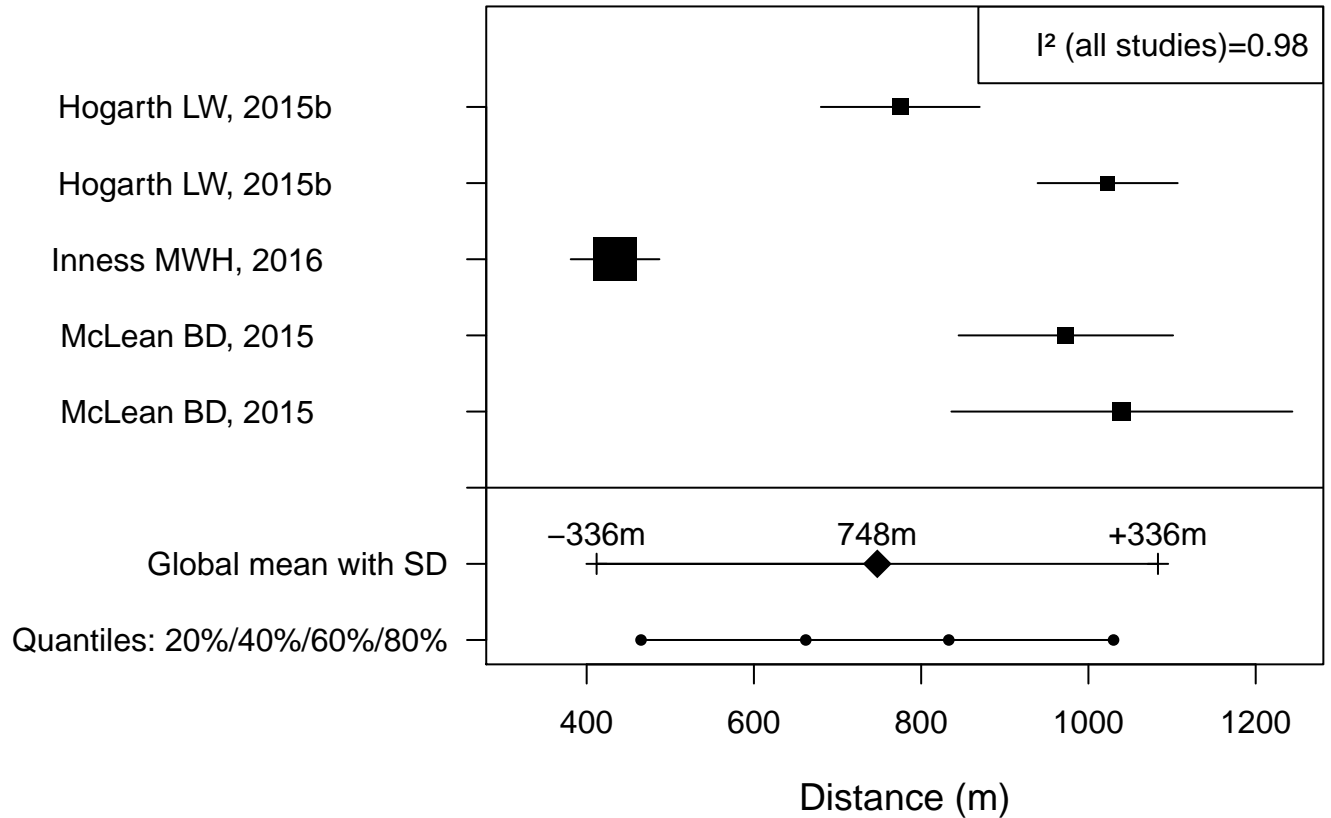

Supplement: Supplementary file 3 [file Data_Sheet_2.ZIP › Forestplots/Forestplot_YYIR2_Football_Male_Amateur or Sub-Elite.pdf]

# Forestplot: YYIR2 Football Male Elite

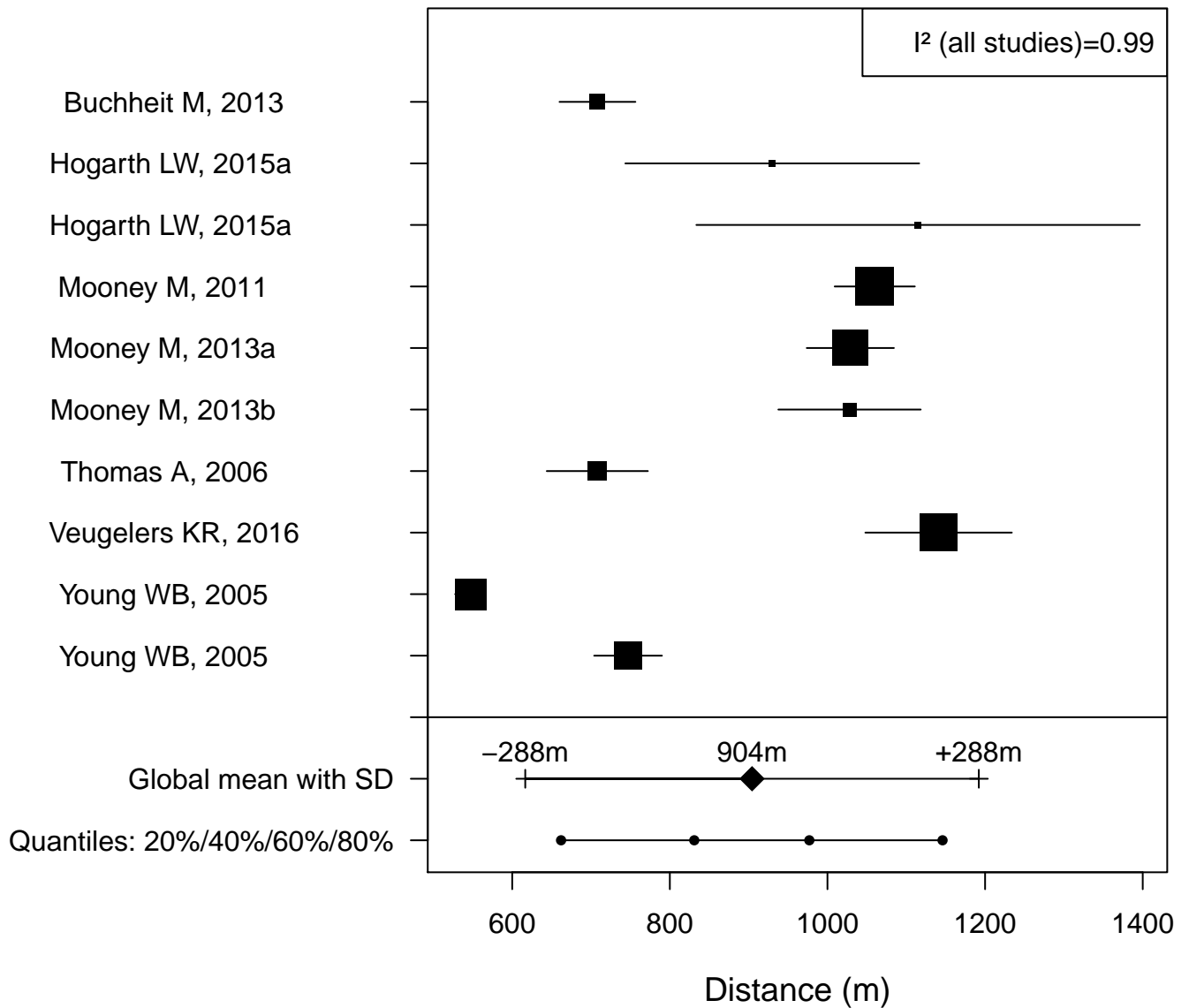

Supplement: Supplementary file 3 [file Data_Sheet_2.ZIP › Forestplots/Forestplot_YYIR2_Football_Male_Elite.pdf]

## Forestplot: YYIR2 Futsal Male Elite

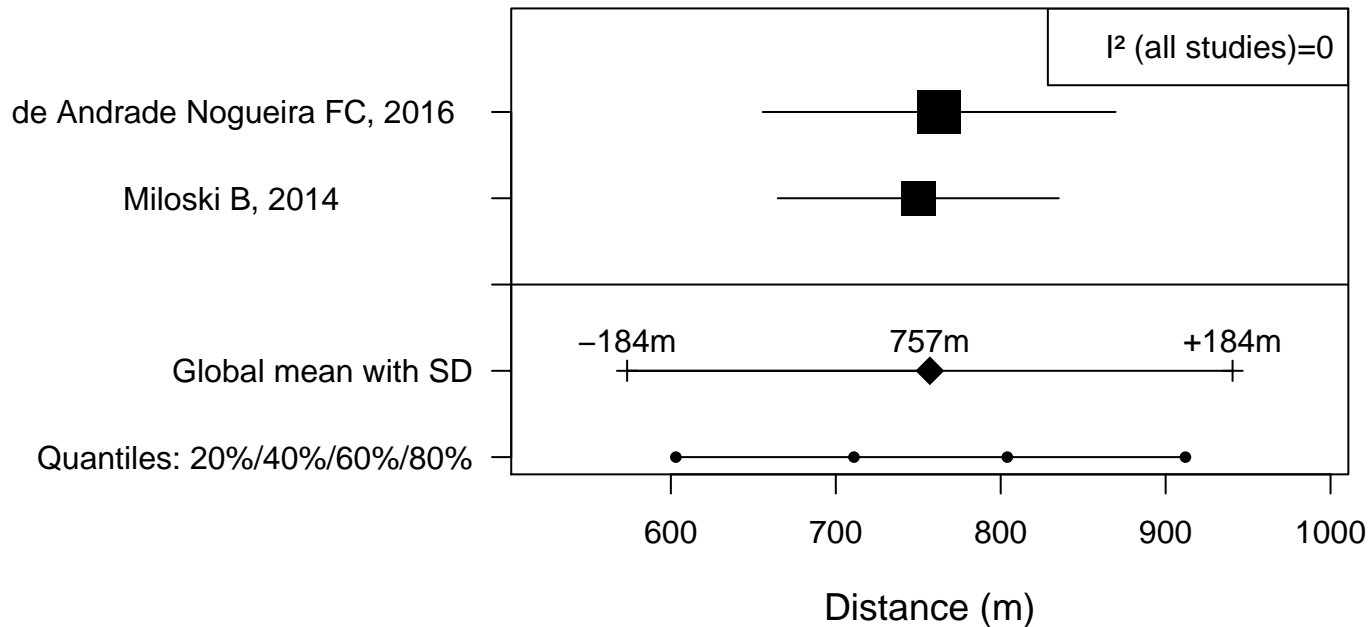

Supplement: Supplementary file 3 [file Data_Sheet_2.ZIP › Forestplots/Forestplot_YYIR2_Futsal_Male_Elite.pdf]

# Forestplot: YYIR2 Handball Male Elite

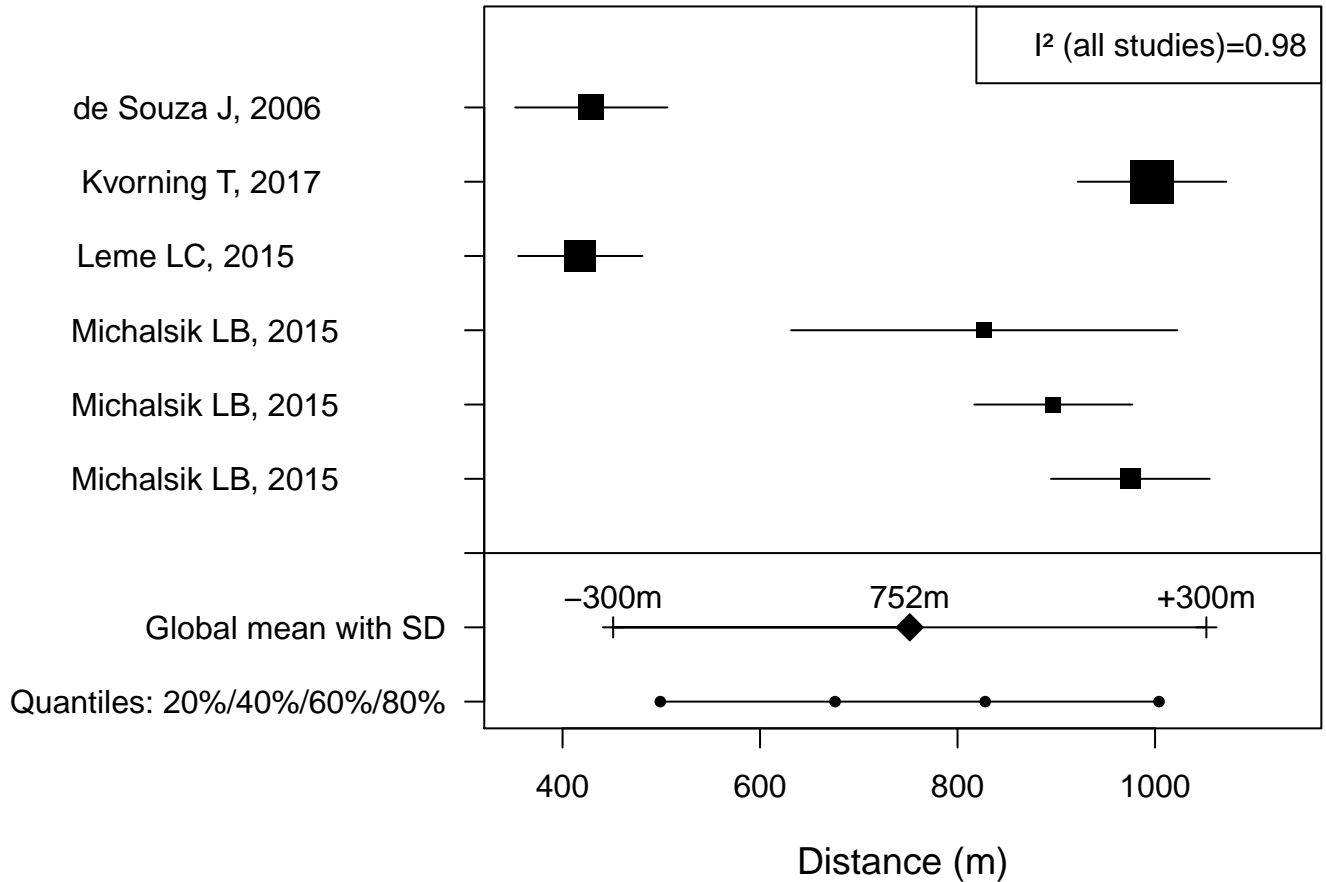

Supplement: Supplementary file 3 [file Data_Sheet_2.ZIP › Forestplots/Forestplot_YYIR2_Handball_Male_Elite.pdf]

## Forestplot: YYIR2 Hockey Male Elite

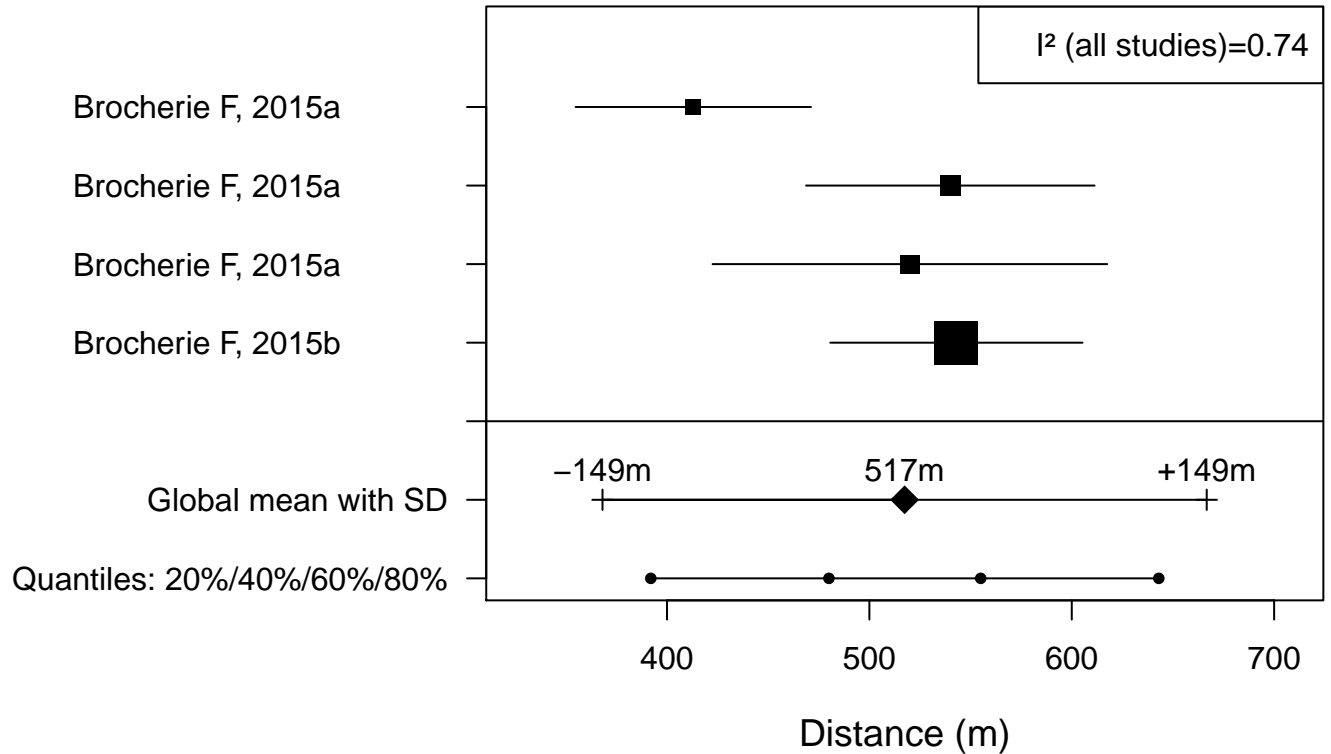

Supplement: Supplementary file 3 [file Data_Sheet_2.ZIP › Forestplots/Forestplot_YYIR2_Hockey_Male_Elite.pdf]

## Forestplot: YYIR2 Recreational Male

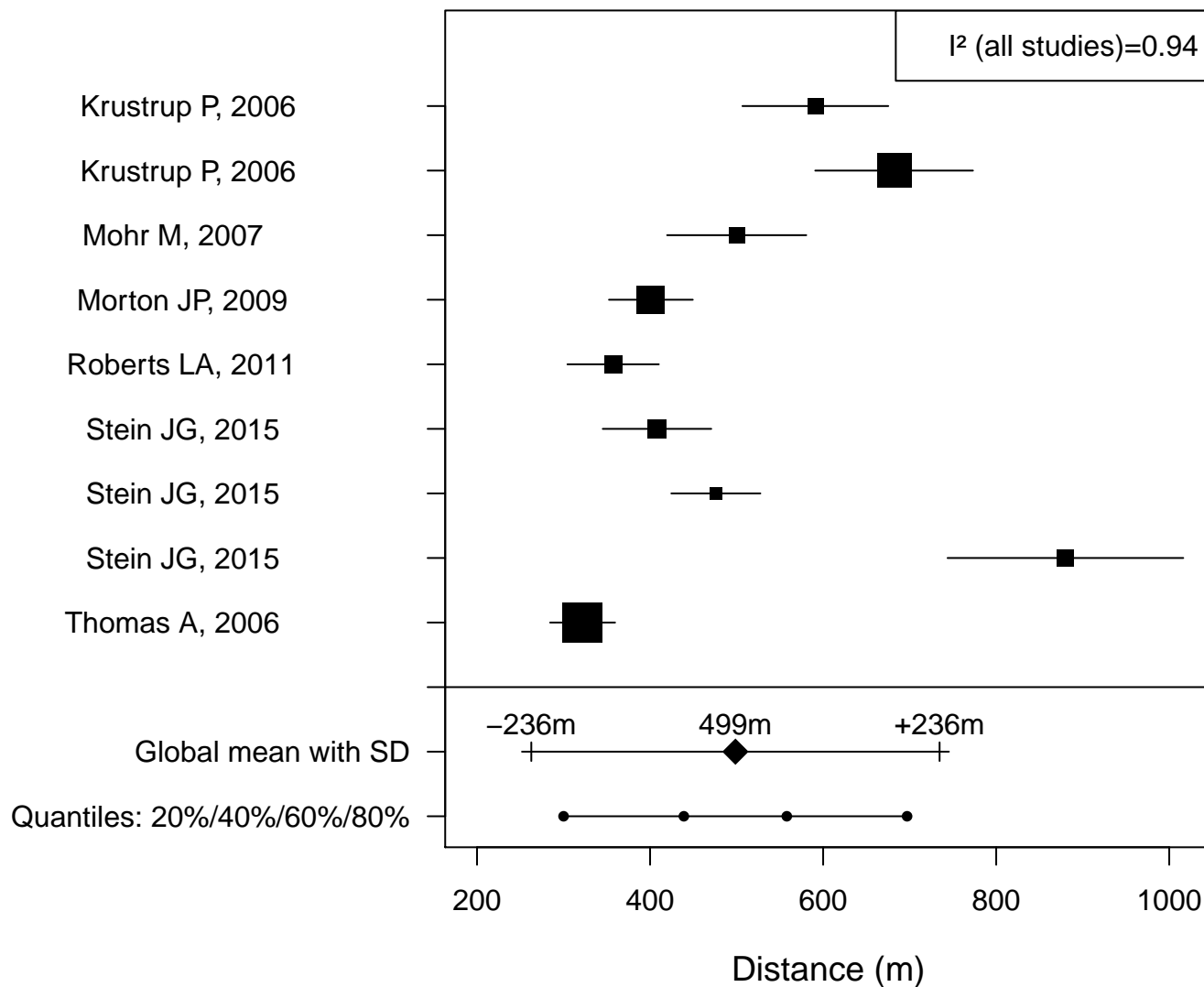

Supplement: Supplementary file 3 [file Data_Sheet_2.ZIP › Forestplots/Forestplot_YYIR2_Recreational_Male_.pdf]

# Forestplot: YYIR2 RS or GF Male Elite

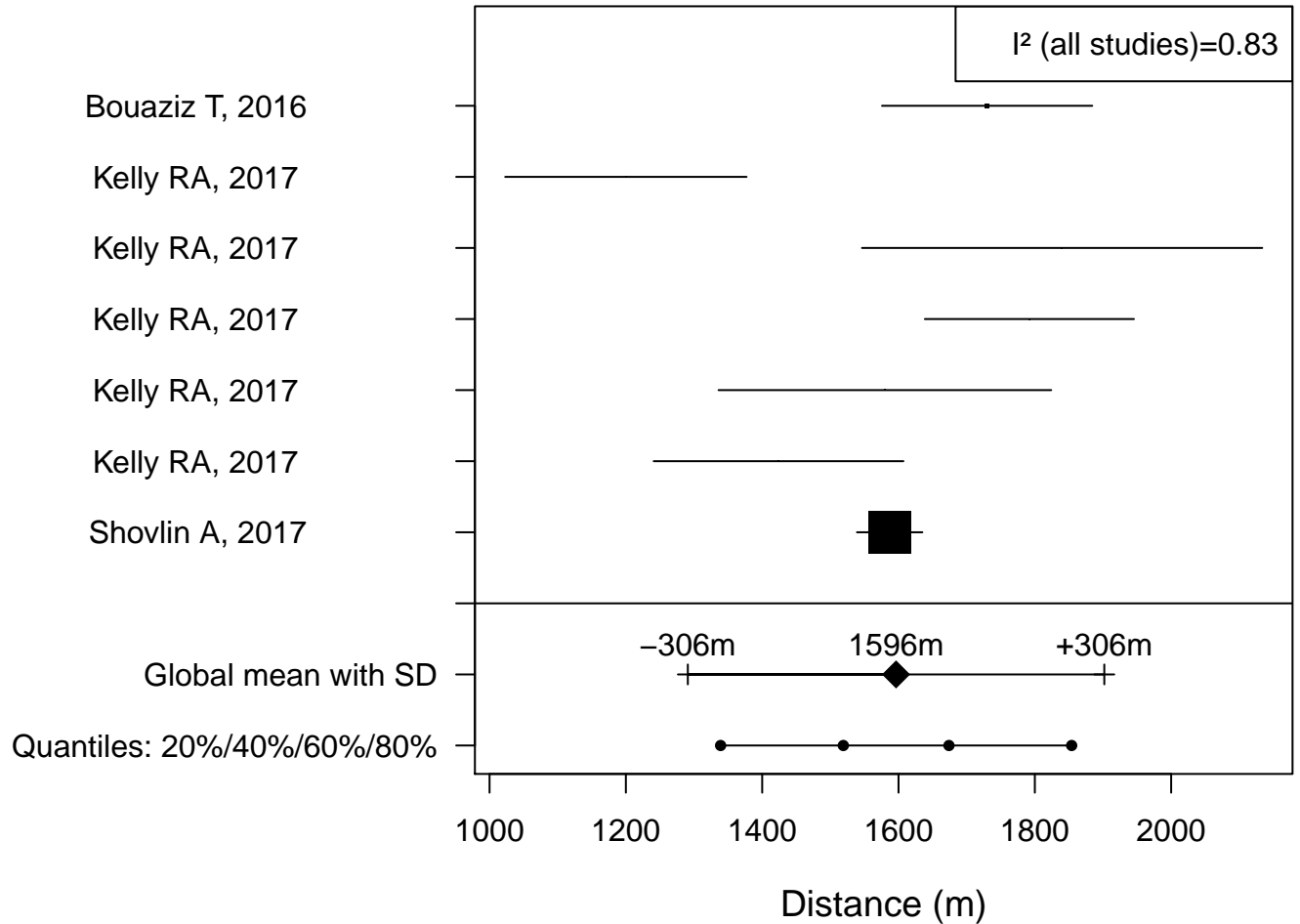

Supplement: Supplementary file 3 [file Data_Sheet_2.ZIP › Forestplots/Forestplot_YYIR2_RS or GF_Male_Elite.pdf]

## Forestplot: YYIR2 Soccer Female Elite

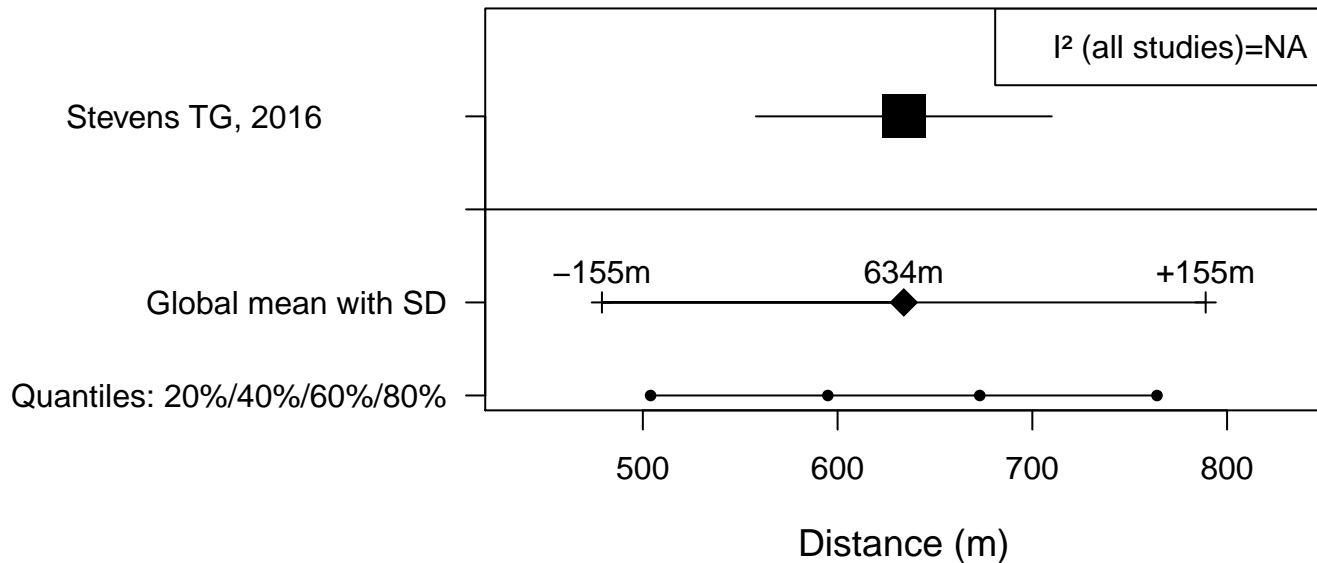

Supplement: Supplementary file 3 [file Data_Sheet_2.ZIP › Forestplots/Forestplot_YYIR2_Soccer_Female_Elite.pdf]

## Forestplot: YYIR2 Soccer Female Sub-Elite

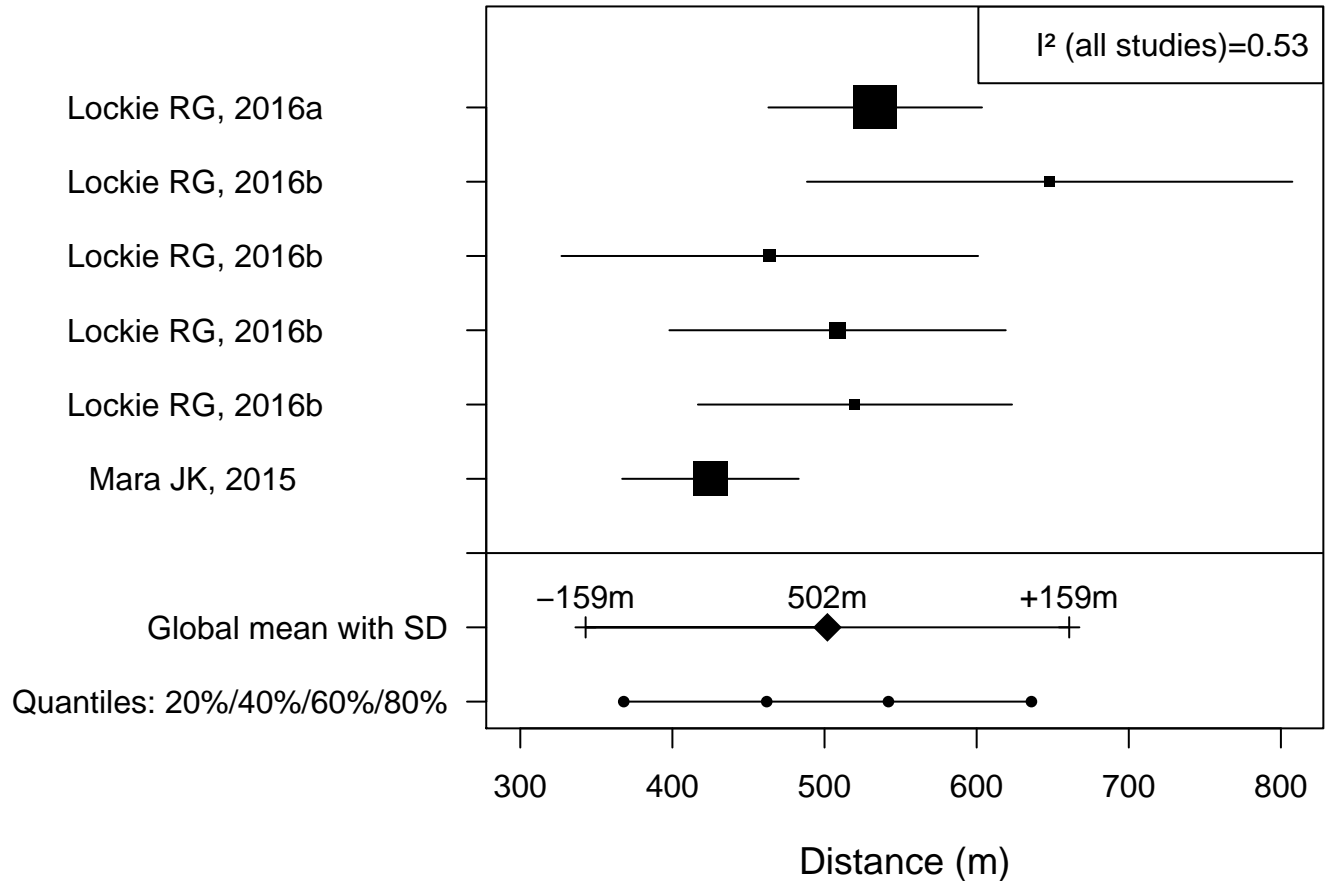

Supplement: Supplementary file 3 [file Data_Sheet_2.ZIP › Forestplots/Forestplot_YYIR2_Soccer_Female_Sub-Elite.pdf]

## Forestplot: YYIR2 Soccer Male Amateur

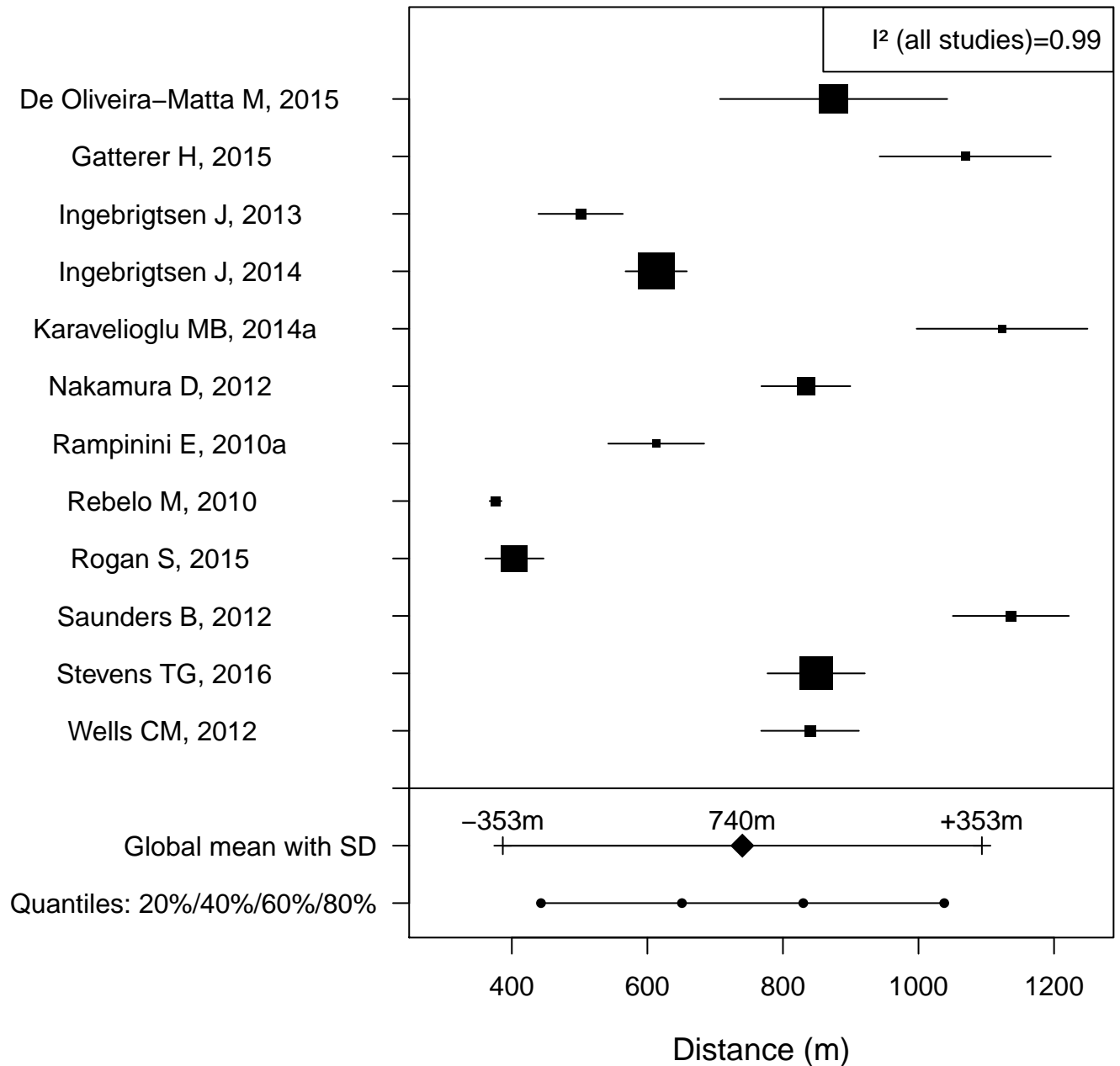

Supplement: Supplementary file 3 [file Data_Sheet_2.ZIP › Forestplots/Forestplot_YYIR2_Soccer_Male_Amateur.pdf]

# Forestplot: YYIR2 Soccer Male Elite

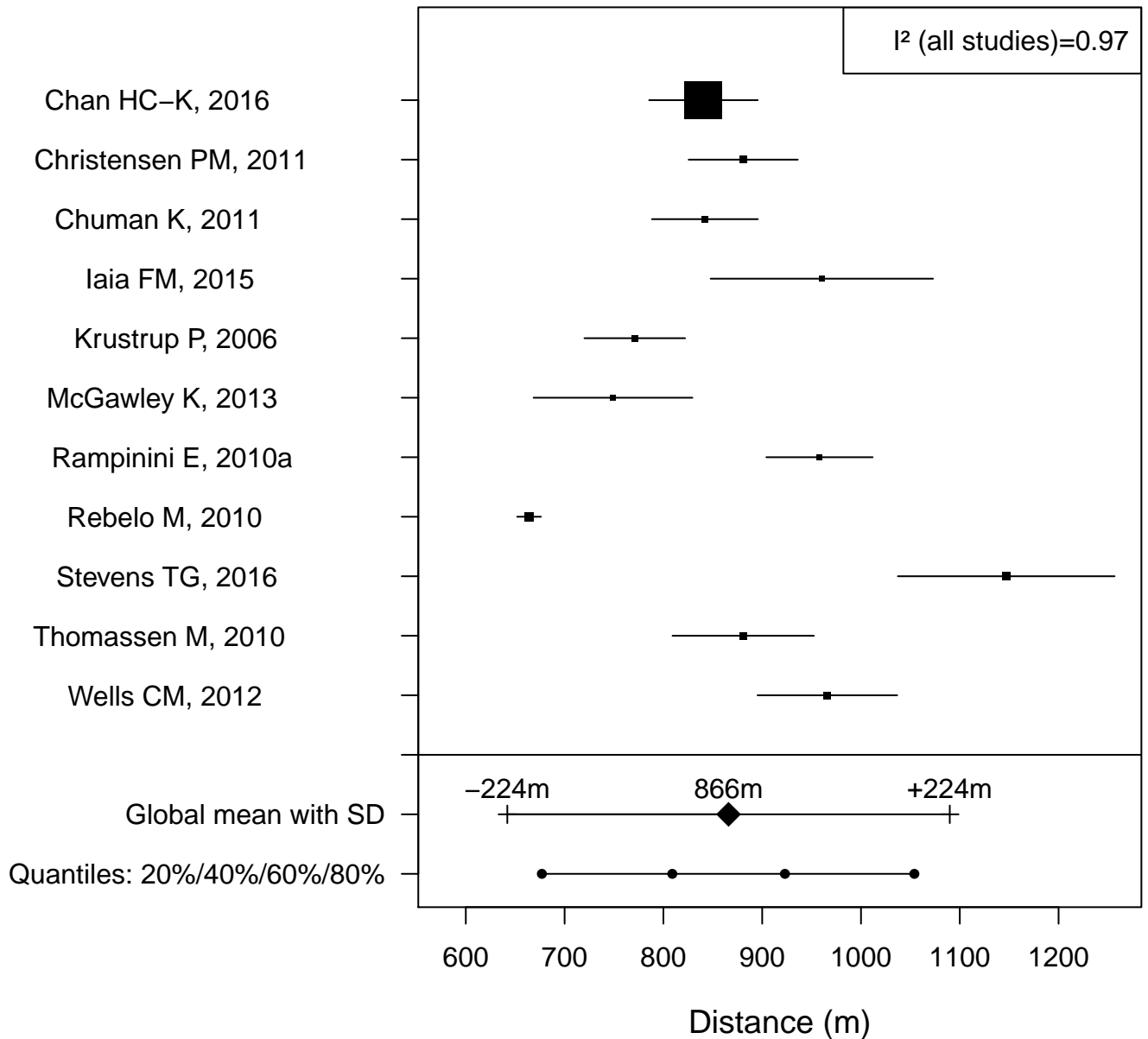

Supplement: Supplementary file 3 [file Data_Sheet_2.ZIP › Forestplots/Forestplot_YYIR2_Soccer_Male_Elite.pdf]

# Forestplot: YYIR2 Soccer Male Sub-Elite

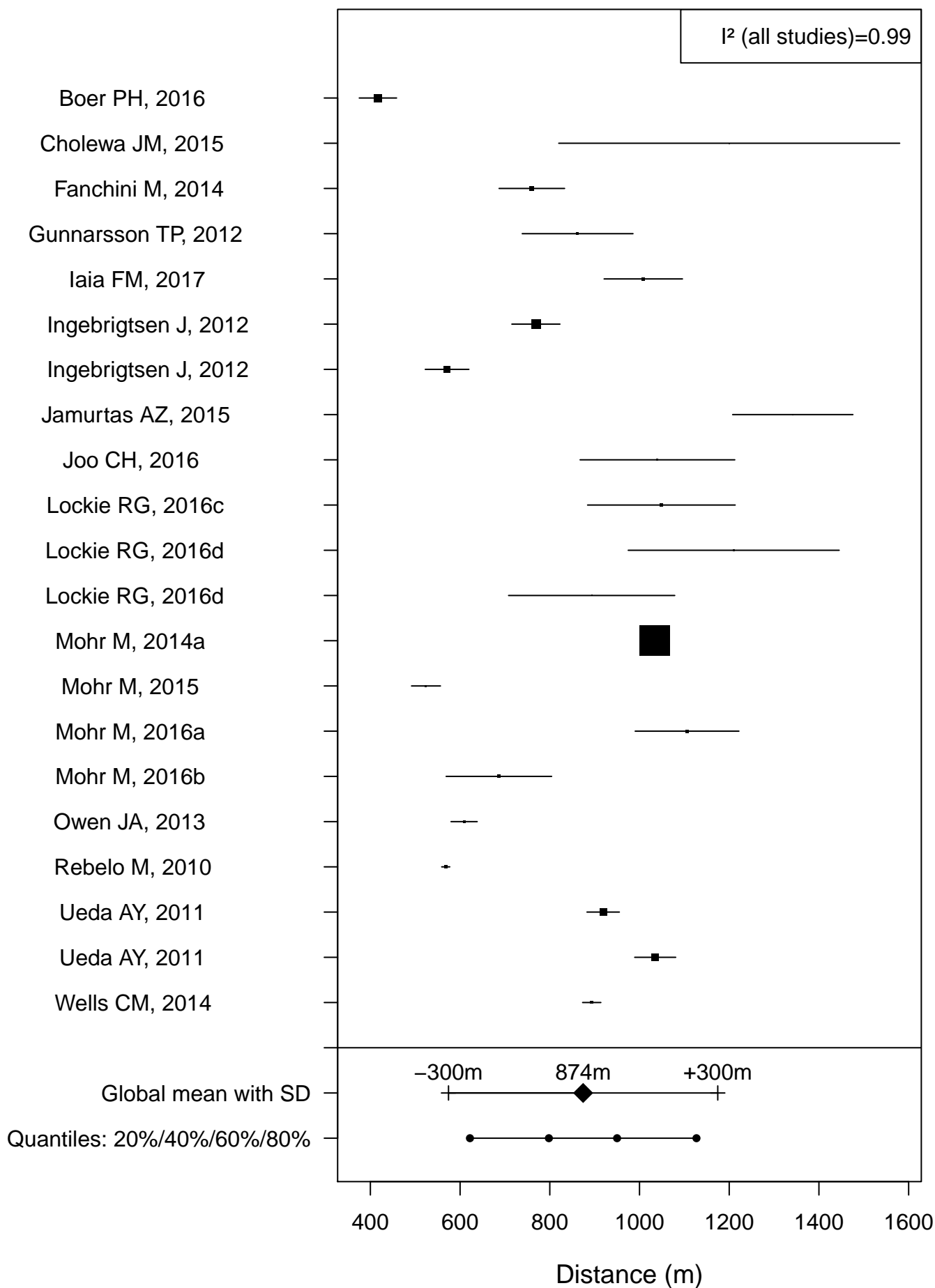

Supplement: Supplementary file 3 [file Data_Sheet_2.ZIP › Forestplots/Forestplot_YYIR2_Soccer_Male_Sub-Elite.pdf]

# Forestplot: YYIR2 Soccer Male Top-Elite

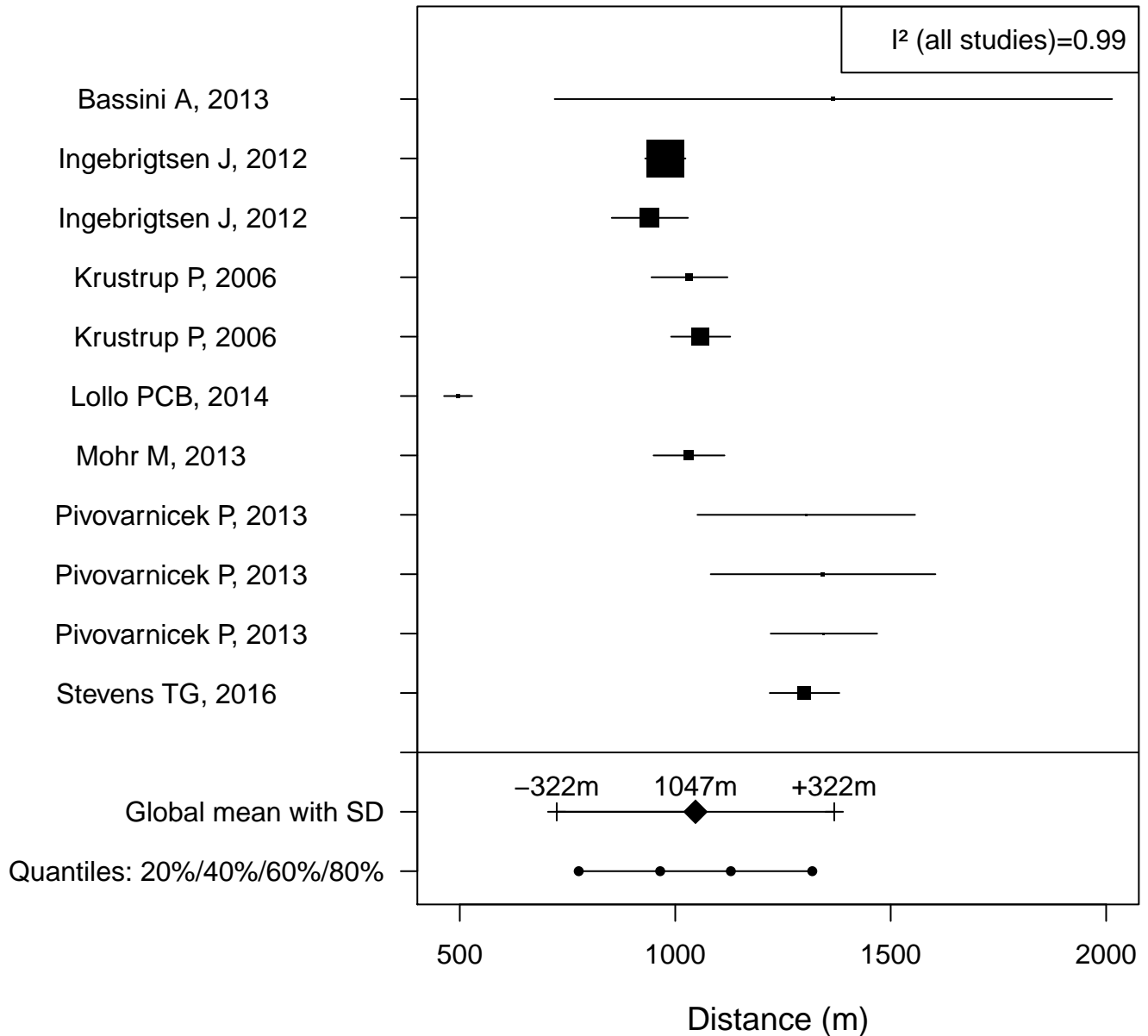

Supplement: Supplementary file 3 [file Data_Sheet_2.ZIP › Forestplots/Forestplot_YYIR2_Soccer_Male_Top-Elite.pdf]

## Forestplot: YYIR2 Ultimate Frisbee Male Sub-Elite

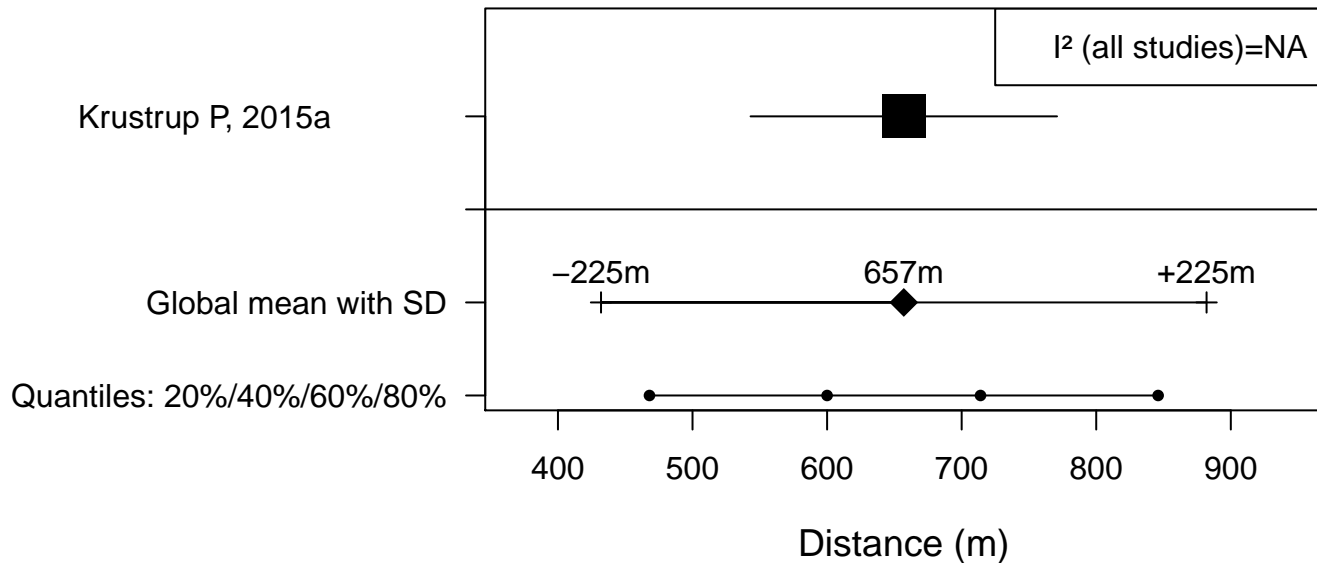

Supplement: Supplementary file 3 [file Data_Sheet_2.ZIP › Forestplots/Forestplot_YYIR2_Ultimate Frisbee_Male_Sub-Elite.pdf]

## Forestplot: YYIR2 Volleyball Female Elite

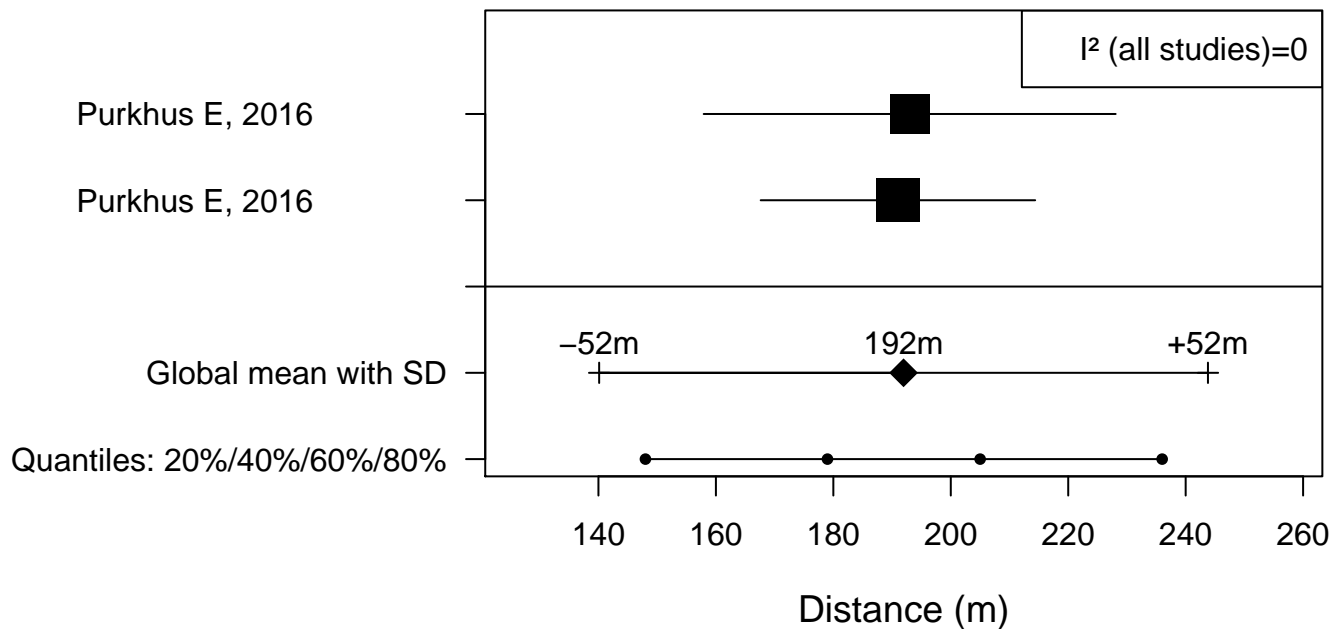

Supplement: Supplementary file 3 [file Data_Sheet_2.ZIP › Forestplots/Forestplot_YYIR2_Volleyball_Female_Elite.pdf]

## Forestplot: YYIR2 X-country Skiing Male Amateur

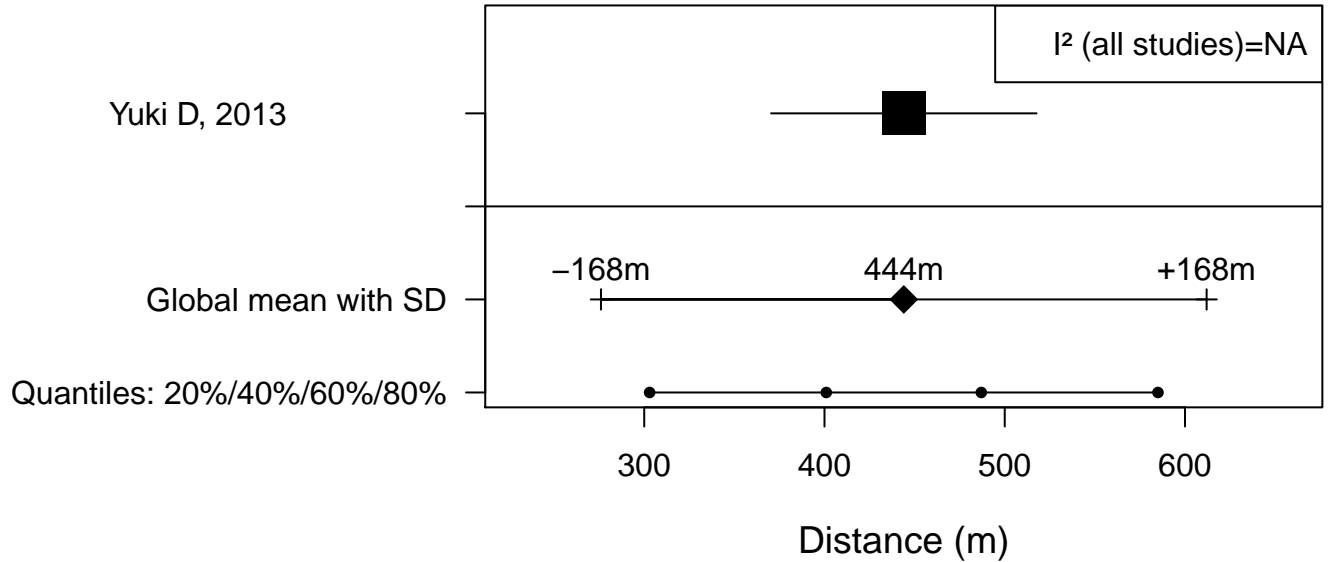

Supplement: Supplementary file 3 [file Data_Sheet_2.ZIP › Forestplots/Forestplot_YYIR2_X-country Skiing_Male_Amateur.pdf]

## Forestplot: YYIR2 X-country Skiing Male Elite

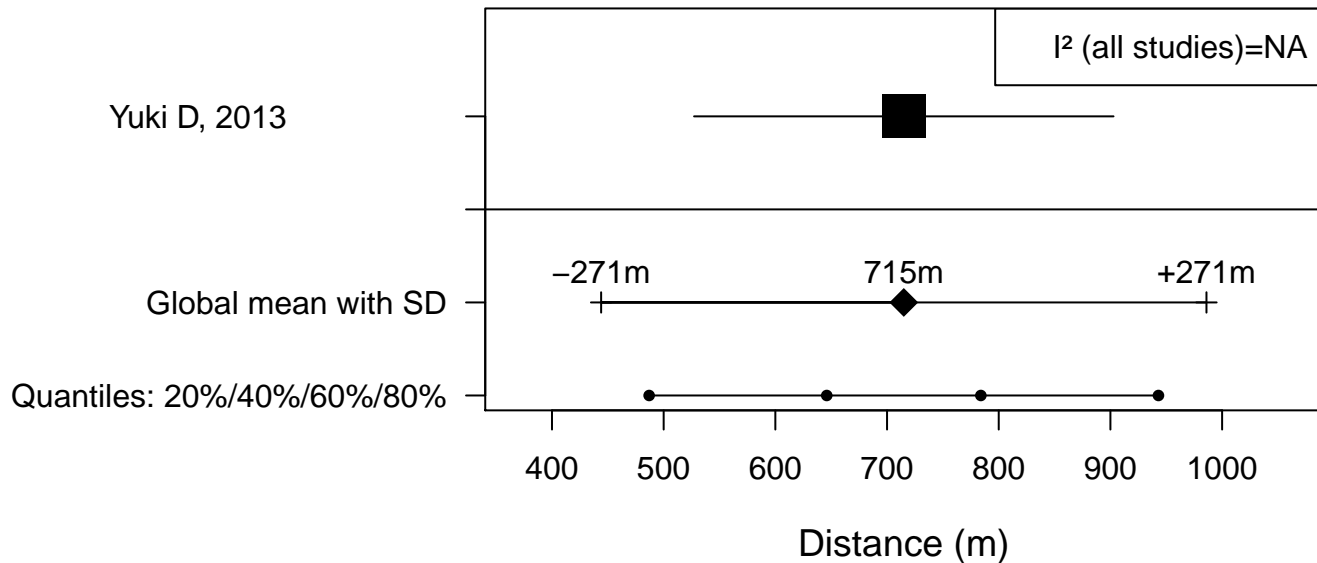

Supplement: Supplementary file 3 [file Data_Sheet_2.ZIP › Forestplots/Forestplot_YYIR2_X-country Skiing_Male_Elite.pdf]

# YYIR1

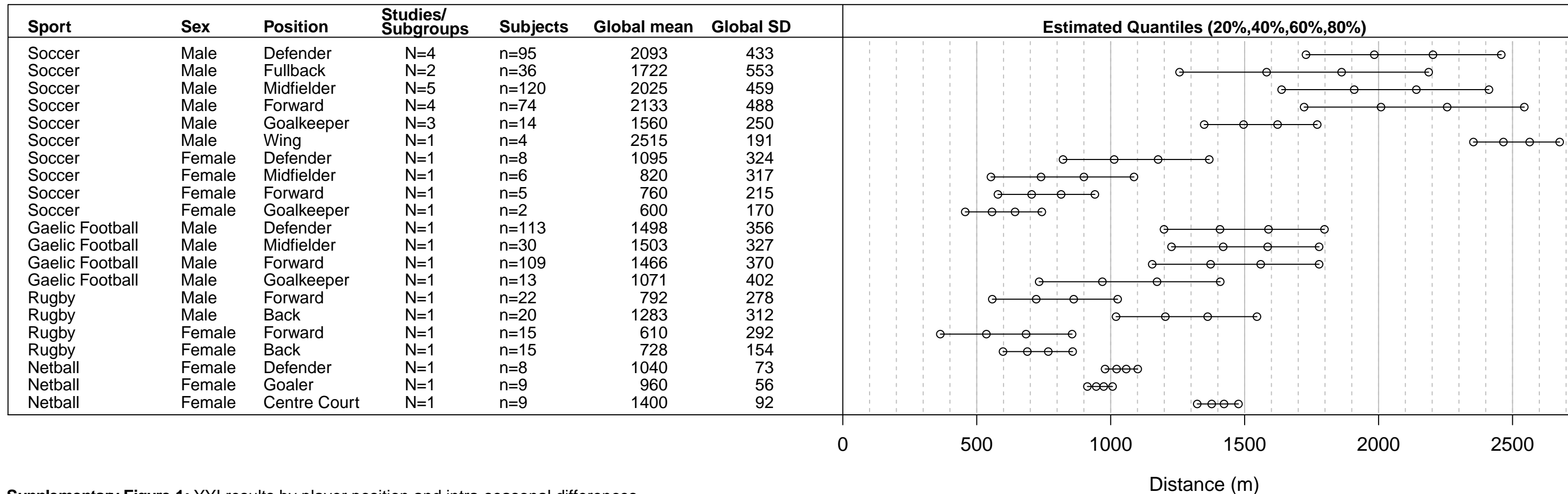

**Supplementary Figure 1:** YYI results by player position and intra-seasonal differences.

Supplement: Supplementary file 4 [file Image_1.PDF]

# YYIR2

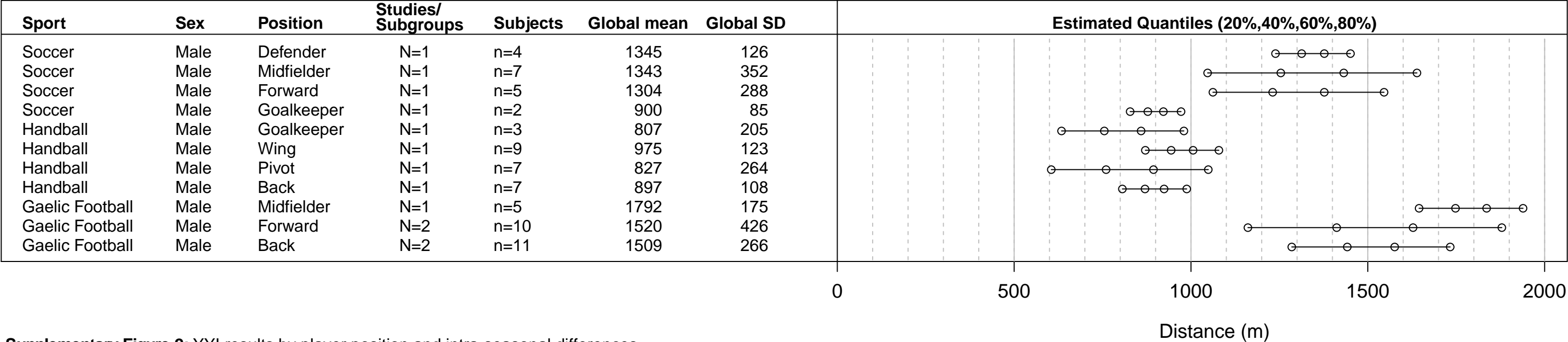

**Supplementary Figure 2:** YYI results by player position and intra-seasonal differences.

Supplement: Supplementary file 5 [file Image_2.PDF]

# YYIE2

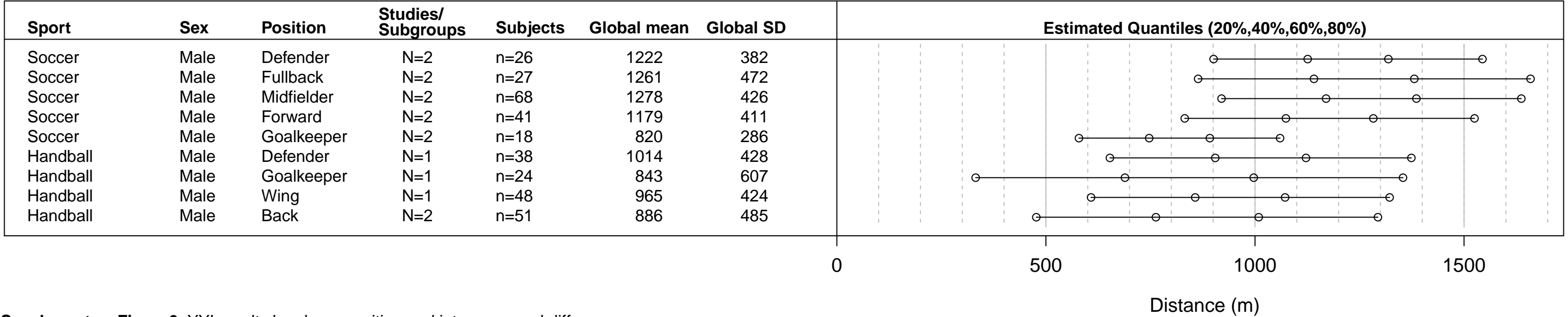

**Supplementary Figure 3:** YYI results by player position and intra-seasonal differences.

Supplement: Supplementary file 6 [file Image_3.PDF]

# YYIR1

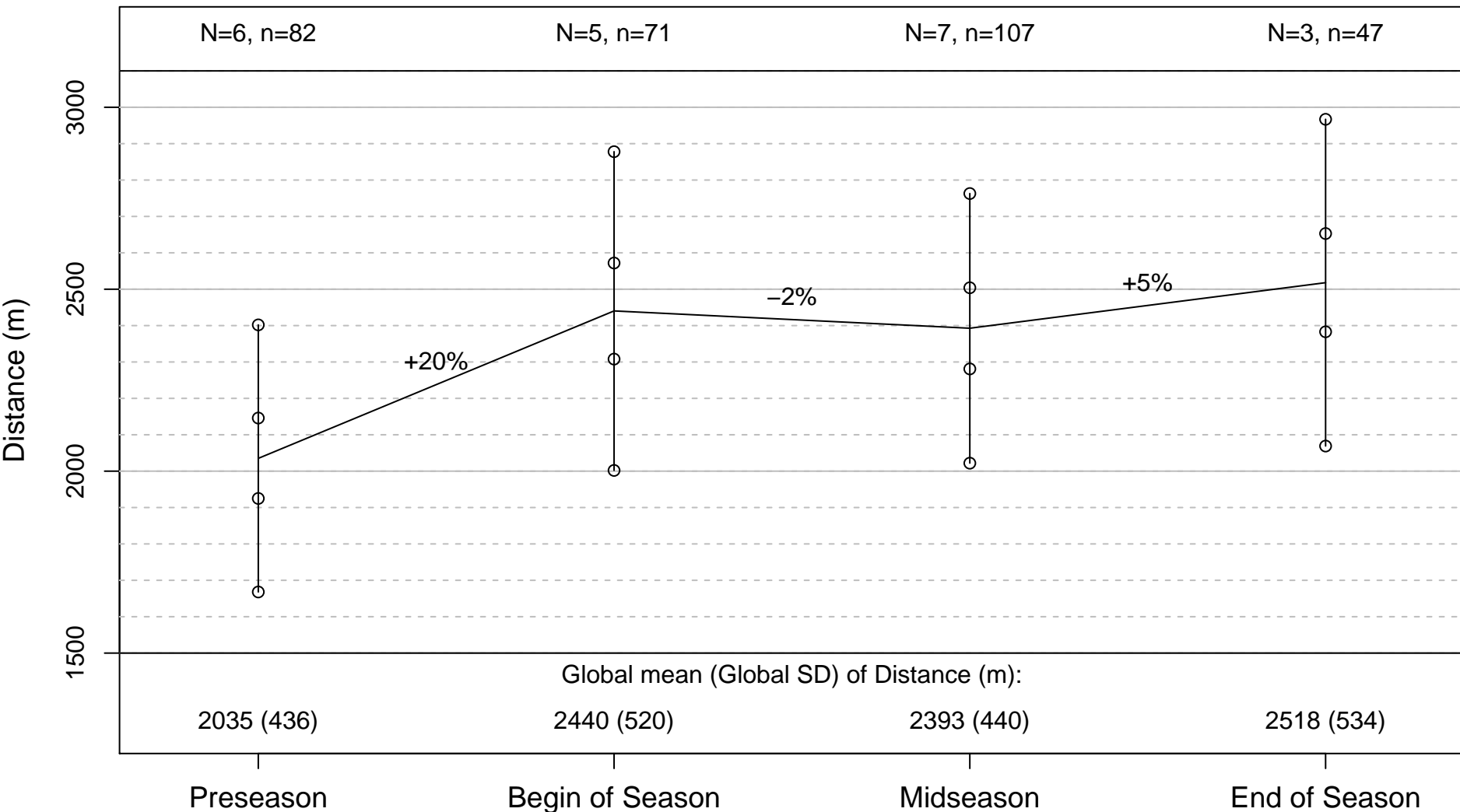

**Supplementary Figure 4:** YYI results by player position and intra-seasonal differences.

Supplement: Supplementary file 7 [file Image_4.PDF]

# YYIR2

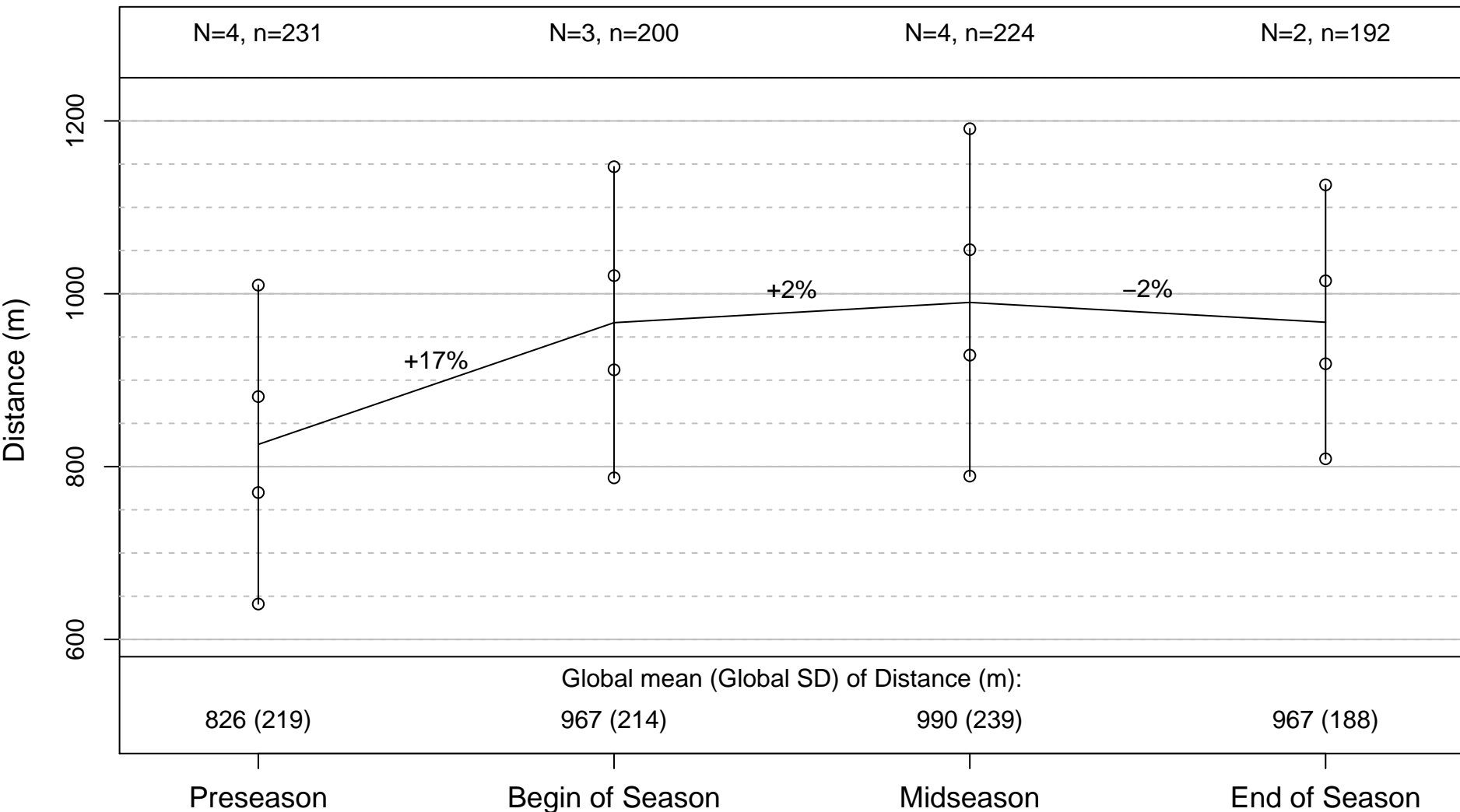

**Supplementary Figure 5:** YYI results by player position and intra-seasonal differences.

Supplement: Supplementary file 8 [file Image_5.PDF]
