# Supplementary material for: The Yo-Yo Intermittent Tests: A Systematic Review and Structured Compendium of Test Results
Source: Front Physiol. 2018 Jul 5;9:870. doi: 10.3389/fphys.2018.00870 (PMC6041409; doi:10.3389/fphys.2018.00870)
Supplement: Supplementary file 3 [file Forestplot_YYIR1_Squash_Male_Elite.pdf › Forestplots/Forestplot_YYIR1_Squash_Male_Elite.pdf]

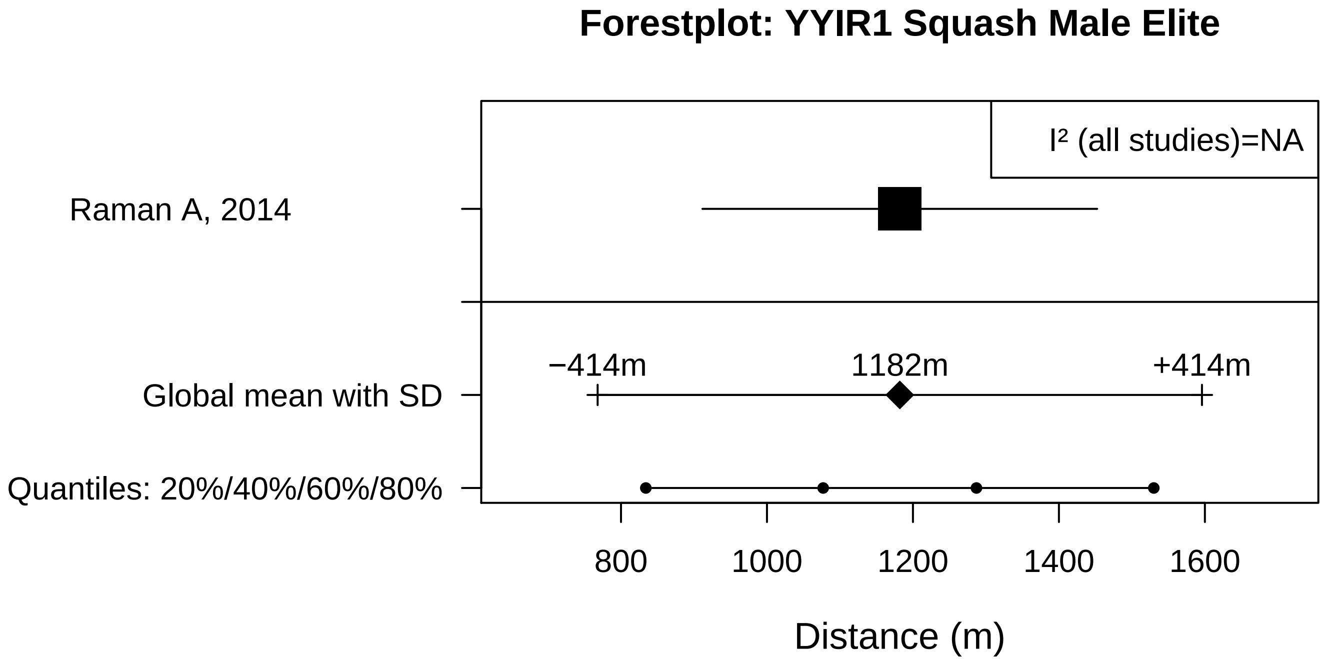
Forestplot: YYIR1 Squash Male Elite
I² (all studies)=NA
Raman A, 2014
−414m
1182m
+414m
Global mean with SD
Quantiles: 20%/40%/60%/80%
800
1000
1200
1400
1600
Distance (m)
